# Minor neuropsychological deficits and stage 2 of Alzheimer's disease

**DOI:** 10.1002/alz.71458

**Published:** 2026-05-13

**Authors:** Melina Stark, Michael Wagner, Elizabeth Kuhn, Sandra Roeske, Holger Amthauer, Claudia Bartels, Henning Boecker, Frederic Brosseron, Ralph Buchert, Katharina Buerger, Marcel Daamen, Alexander Drzezga, Emrah Düzel, Ersin Ersözlü, Markus Essler, Michael Ewers, Klaus Fliessbach, Wenzel Glanz, Julian Hellmann‐Regen, Enise I. Incesoy, Daniel Janowitz, Konstantinia Kafali, Ingo Kilimann, Bernd Joachim Krause, Marie Kronmüller, Christoph Laske, Franziska Maier, Angelika Maurer, Jochen Michely, Robert Perneczky, Oliver Peters, Lukas Preis, Josef Priller, Boris‐Stephan Rauchmann, Matthias Reimold, Axel Rominger, Matthias Schmid, Anja Schneider, Sebastian Sodenkamp, Annika Spottke, Eike Jakob Spruth, Stefan Teipel, Jens Wiltfang, for the Alzheimer´s Disease Neuroimaging Initiative (ADNI), Frank Jessen, Luca Kleineidam

**Affiliations:** ^1^ Department of Old Age Psychiatry and Cognitive Disorders University Hospital Bonn, University Bonn Bonn Germany; ^2^ German Center for Neurodegenerative Diseases (DZNE) Bonn Bonn Germany; ^3^ Department of Nuclear Medicine Campus Virchow‐Klinikum Charité – Universitätsmedizin Berlin Berlin Germany; ^4^ Department of Psychiatry and Psychotherapy University Medical Center Goettingen University of Goettingen Goettingen Germany; ^5^ Clinical Functional Imaging Group Department of Nuclear Medicine University Hospital Bonn Bonn Germany; ^6^ Department of Diagnostic and Interventional Radiology and Nuclear Medicine University Medical Center Hamburg‐Eppendorf Hamburg Germany; ^7^ German Center for Neurodegenerative Diseases (DZNE) Munich Munich Germany; ^8^ Institute for Stroke and Dementia Research (ISD) University Hospital, LMU Munich Munich Germany; ^9^ Department of Nuclear Medicine Faculty of Medicine and University Hospital Cologne University of Cologne Cologne Germany; ^10^ Institute of Neuroscience and Medicine (INM‐2) Molecular Organization of the Brain, Forschungszentrum Jülich Jülich Germany; ^11^ German Center for Neurodegenerative Diseases (DZNE) Magdeburg Magdeburg Germany; ^12^ Institute of Cognitive Neurology and Dementia Research (IKND) Otto‐von‐Guericke University Magdeburg Germany; ^13^ German Center for Neurodegenerative Diseases (DZNE) Berlin Berlin Germany; ^14^ Department of Psychiatry and Psychotherapy Campus Benjamin Franklin Charité – Universitätsmedizin Berlin Berlin Germany; ^15^ ECRC Experimental and Clinical Research Center Charité – Universitätsmedizin Berlin Berlin Germany; ^16^ Department of Nuclear Medicine University Hospital Bonn Bonn Germany; ^17^ Department of Neuropsychiatry Department of Psychiatry and Psychotherapy Campus Charité Mitte Charité – Universitätsmedizin Berlin Berlin Germany; ^18^ German Center for Neurodegenerative Diseases (DZNE) Rostock Rostock Germany; ^19^ Department of Psychosomatic Medicine Rostock University Medical Center Rostock Germany; ^20^ Department of Nuclear Medicine Rostock University Medical Centre Rostock Germany; ^21^ German Center for Neurodegenerative Diseases (DZNE) Tuebingen Tuebingen Germany; ^22^ Section for Dementia Research Hertie Institute for Clinical Brain Research and Department of Psychiatry and Psychotherapy University of Tuebingen Tuebingen Germany; ^23^ Department of Psychiatry Medical Faculty University of Cologne Cologne Germany; ^24^ Department of Psychiatry and Psychotherapy University Hospital LMU Munich Munich Germany; ^25^ Munich Cluster for Systems Neurology (SyNergy) Munich Germany; ^26^ Ageing Epidemiology Research Unit (AGE) School of Public Health Imperial College London London UK; ^27^ Department of Psychiatry and Psychotherapy School of Medicine Technical University of Munich Munich Germany; ^28^ University of Edinburgh and UK DRI Edinburgh UK; ^29^ Department of Neuroradiology University Hospital, LMU Munich Munich Germany; ^30^ Sheffield Institute for Translational Neuroscience (SITraN) University of Sheffield Sheffield UK; ^31^ Department of Nuclear Medicine and Clinical Molecular Imaging, Eberhard‐Karls‐University Tuebingen Germany; ^32^ Department of Nuclear Medicine Ludwig‐Maximilian‐University Munich Munich Germany; ^33^ Department of Nuclear Medicine Inselspital, Bern University Hospital, University of Bern Bern Switzerland; ^34^ Institute for Medical Biometry, Informatics and Epidemiology University Hospital Bonn Bonn Germany; ^35^ Department of Psychiatry and Psychotherapy University of Tuebingen Tuebingen Germany; ^36^ Department of Neurology University of Bonn Medical Center Bonn Germany; ^37^ German Center for Neurodegenerative Diseases (DZNE) Goettingen Goettingen Germany; ^38^ Neurosciences and Signaling Group Institute of Biomedicine (iBiMED) Department of Medical Sciences University of Aveiro Aveiro Portugal; ^39^ Excellence Cluster on Cellular Stress Responses in Aging‐Associated Diseases (CECAD) University of Cologne Cologne Germany

**Keywords:** clinical staging, minor neuropsychological deficits, preclinical Alzheimer's disease, stage 2 of Alzheimer's disease, subjective cognitive concerns, subjective cognitive decline, subtle cognitive decline, transitional cognitive decline

## Abstract

**INTRODUCTION:**

Subtle symptoms, like subjective cognitive decline (SCD) and minor neuropsychological deficits (MNPD), can improve the risk stratification in preclinical Alzheimer´s disease (AD) but their importance is insufficiently elaborated.

**METHODS:**

We pooled data from cognitively normal individuals participating in three longitudinal cohort studies (*N* = 13,192, 8,359[63.3%] female, mean [SD] age 71.0[8.4]).

**RESULTS:**

Compared to participants without SCD and MNPD (SCD‐/MNPD‐), SCD‐/MNPD+, SCD+/MNPD‐, and SCD+/MNPD+ participants had an increased risk for mild cognitive impairment (MCI) and dementia, including in amyloid‐positive individuals. Focusing on SCD+/MNPD+ participants triples the positive predictive value of amyloid biomarker testing for the 5‐year prediction of MCI and reduces the required samples size for trials in preclinical AD to one fourth, compared to considering all cognitively normal participants regardless of subtle symptoms.

**DISCUSSION:**

SCD and MNPD offer a powerful approach for risk stratification in preclinical AD, which can improve clinical trial designs, risk counseling, and future case identifications for early treatment.

## Highlights

- Clinical progression risk is increased in minor neuropsychological deficits (MNPD).
- This risk increase is higher in combination with subjective cognitive decline (SCD).
- SCD and MNPD relate to an increased dementia risk in amyloid‐positive individuals.
- SCD and MNPD improve the prognostic utility of amyloid biomarker testing.
- SCD and MNPD reduce sample requirements for clinical trials by at least a third.

## 1 BACKGROUND

The core proteinopathies underlying Alzheimer's disease (AD) develop over decades before the onset of dementia.^1^ Similarly, symptoms of AD emerge gradually, already before the onset of mild cognitive impairment (MCI).^2^ This continuous decline is recognized in the distinction between asymptomatic individuals (stage 1) and those with subtle symptoms (stage 2) within the preclinical phase of the disease in the Alzheimer's Association's diagnostic criteria.^3^ Cognitively normal individuals (CN) with preclinical AD, comprising AD stage 1 and 2, are at increased risk of cognitive decline compared to CN without AD pathology, but progression rates vary substantially.^4^, ^5^, ^6^

Subtle symptoms can improve risk stratification among CN populations. Subjective cognitive decline (SCD) is a symptom of AD stage 2 and predictor of cognitive decline, with an incremental prognostic value beyond amyloid pathology.^7^, ^8^, ^9^ Additionally, baseline cognitive performance in CN predicts cognitive decline beyond AD biomarkers.^10^, ^11^ To operationalize subtle cross‐sectional cognitive symptoms, we previously proposed criteria for minor neuropsychological deficits (MNPD) and showed their association with clinical progression.^12^ Preliminary evidence suggests that the combined assessment of SCD and MNPD enhances their prognostic power, but research about this topic is limited.^12^, ^13^, ^14^, ^15^

A better understanding of the predictive relevance of these subtle symptoms will support the refinement of clinical staging approaches in AD.^3^, ^16^ Effective clinical staging, grounded in knowledge about differing risks of progression at the early stages of AD, is vital for the design of novel treatment trials aimed at slowing cognitive decline from the preclinical stage onward (NCT05026866, NCT04468659). It can also serve fine‐graded individual risk estimations in dementia prevention initiatives, like the recently proposed Brain Health Services.^17^, ^18^ Finally, it will aid discussions regarding the cost‐benefit‐risk ratios of disease‐modifying treatments in preclinical AD.^16^

In this study, we quantified the independent and combined associations of SCD and MNPD in CN with AD pathology and clinical progression, including in amyloid‐positive individuals. Additionally, we investigated their impact on the short‐term prognostic value of amyloid positivity as well as on sample size estimates for clinical trials in preclinical AD.

**RESEARCH IN CONTEXT**

1. **Systematic review**: A literature review using the PubMed database was conducted. Previous studies showed that minor neuropsychological deficits (MNPD) and subjective cognitive decline (SCD) are associated with Alzheimer's disease (AD) pathology and cognitive decline. Initial results on the diagnostic and predictive utility of the combination of both subtle symptoms are limited and inconsistent.
2. **Interpretation**: In this pooled sample of 13,192 cognitively normal participants from three longitudinal cohort studies, SCD and MNPD were associated with an increased risk of clinical progression, including in amyloid‐positive individuals, improved the prediction of mild cognitive impairment by amyloid pathology, and reduced sample size estimations for clinical trials.
3. **Future directions**: Building on our results showing how subtle symptoms can amplify the efficiency of clinical trials and the information gained from biomarker testing, future research should investigate their combined value with biological AD staging using Tau PET and explore the importance of neuropsychiatric symptoms for clinical staging.

## 2 METHODS

### 2.1 Study design and participants

In this longitudinal cohort study, we analyzed data, collected between June 2005 and May 2024, from up to 13,192 CN recruited from memory clinics or communities. We used data from the German Center for Neurodegenerative Diseases Longitudinal Cognitive Impairment and Dementia study (DELCODE; *N* = 618),^19^ the Alzheimer's Disease Neuroimaging Initiative (ADNI; *N* = 599),^20^ and the National Alzheimer's Coordinating Center database (NACC; *N* = 11,975).^21^ Detailed descriptions of the procedures and sample selection are provided in the supplement (Figures S1–S3). The participants in these datasets were at least 50 years old, did not receive a study diagnosis of MCI or dementia at baseline, and had AD biomarker and/or clinical progression data available. Additionally, they did not meet our operational criterion for MCI (see below) at baseline, to ensure that participants from all cohorts met the same standard for cognitive normality. We followed the Strengthening the Reporting of Observational Studies in Epidemiology (STROBE) guidelines.

### 2.2 Clinical classifications

In accordance with established criteria, SCD was defined by the presence of self‐experienced cognitive decline in individuals without objective cognitive test deficits.^22^ In DELCODE, the study's participant groups indicated the presence of cognitive concerns. This cohort included memory clinic patients, who were cognitively normal and sought medical advice due to self‐experienced decline in any cognitive domain, and SCD‐free control participants recruited from the community. In ADNI‐2 and ADNI‐3, we used data from the Everyday Cognition Questionnaire (ECog), which assesses subjective decline in six cognitive domains, and defined SCD by a value ≥1.35 in the ECog total score. This threshold was derived from DELCODE participants to ensure similar levels of cognitive concerns across these cohorts (Supplementary Methods). In ADNI‐1, the Geriatric Depression Scale (GDS) item “Do you feel you have more problems with memory than most?” was available to indicate SCD. In NACC, SCD was defined by a report of subjectively perceived memory decline to the study physicians.

For the operationalization of MNPD, we built on our previous work, which used a cutoff at z≤‐0.5 in a composite score calculated from the Consortium to Establish a Registry for Alzheimer´s Disease (CERAD) neuropsychological battery.^12^ This approach was not applicable to all cohorts in the present analyses due to differing neuropsychological batteries. Additionally, it was not possible to define MNPD based on composite measures of each cohort's cognitive test battery, since normative data for these scores are not available. We, therefore, chose a criterion depending on the number of low test scores for a test battery‐independent definition of MNPD, which was adapted from an established approach for the diagnosis of MCI.^23^ Analogous to our mean‐based criterion used previously, we required at least half of the tests of a given neuropsychological battery to have a demographically‐adjusted, normative performance of z≤‐0.5—corresponding to a median of z≤‐0.5—to approximate the central tendency of normative performance across each test battery. Previously published age‐, sex‐, and education‐adjusted, regression‐based test norms^24^, ^25^, ^26^, ^27^ were used to derive *z*‐scores for this operationalization of MNPD (Table S1).

Progression to MCI was coded in participants with a clinical diagnosis of cognitive impairment and/or a Clinical Dementia Rating (CDR) global score ≥0.5 at follow‐up. In these individuals, we applied a harmonized operational MCI criterion to ensure a consistent standard of cognitive impairment across all cohorts (Supplementary Methods). This operational criterion was met if at least two tests in the same cognitive domain were classified as impaired, one with a demographically‐adjusted *z*‐score ≤‐1.5 and another with a *z*‐score ≤‐1 (Table S1). In line with our previous work,^12^ this modified adaptation of a widely used MCI criterion, requiring two tests with a *z*‐score ≤‐1 in the same cognitive domain,^28^ was chosen because some domains in our data included more than two tests—which allows for a more comprehensive neuropsychological assessment, but increases the chance of abnormal test scores in healthy individuals.^23^, ^29^, ^30^ In all cohorts, progression to dementia was diagnosed by study physicians based on established diagnostic criteria (Supplementary Methods).^31^

### 2.3 AD biomarkers

Amyloid and tau pathology were operationalized with cerebrospinal fluid (CSF) or positron emission tomography (PET) data using study‐dependent cutoffs. We prioritized PET over CSF if both were available. In DELCODE, CSF biomarkers were measured with Fujirebio Innotest® assays and amyloid PET was measured with [18F]florbetaben (Supplementary Methods).^19^ Cutoff values for the CSF biomarkers were previously derived using mixture‐modelling.^7^ Amyloid pathology was defined by a CSF amyloid‐β42/40 (Aβ42/40) ratio ≤0.08 or a Centiloid score ≥24.4.^32^ Tau pathology was defined by CSF *p*‐tau_181_ ≥24.30 (pg/ml). In ADNI, CSF biomarkers were measured with Roche Elecsys® immunoassays, amyloid PET was measured with [18F]florbetaben or [18F]florbetapir, and tau PET was measured with [18F]flortaucipir (Supplementary Methods).^20^ CSF cutoff values anchored to amyloid and tau PET positivity have previously been published.^33^ Cutoff values for amyloid PET are provided by the ADNI PET Core. Consistent with the methodology of other research groups, we defined tau PET positivity as a meta‐temporal standardized uptake value ratio (SUVR) ≥2SD above the mean of amyloid PET‐negative CN participants.^5^, ^6^ Amyloid pathology was defined as CSF Aβ42 ≤ 981 (pg/ml), a [18F]florbetapir SUVR ≥1.11, or a [18F]florbetaben SUVR ≥1.08. Tau pathology was defined as CSF *p*‐tau_181_ ≥24.30 (pg/ml) or a [18F]flortaucipir SUVR ≥1.30. In NACC, we used data from study physician reports of PET and/or CSF amyloid and tau positivity (Supplementary Methods). These dichotomous records indicated whether biomarker abnormalities had been found based on local assessment procedures and standards for biomarker positivity. NACC CSF reports did not differentiate between total and phosphorylated tau. We used the first visit with available biomarker data as the baseline for biomarker‐related analyses in NACC, since the collection of these data was initiated with a later study phase in 2015.

### 2.4 Statistical analysis

For the statistical analyses, we defined four symptomatic groups based on the absence or presence of SCD and MNPD. Using Cox regressions, we first compared these groups in their risk of progression to MCI and dementia over 10 years. Cox regression models with Firth's penalized maximum likelihood estimation were used to address non‐convergence due to monotone likelihood in models with no events in one analytical group.^34^ Additionally, we compared the groups’ restricted mean survival times (RMST), which reflected the average time to clinical progression over 10 years. These analyses were conducted with pseudo‐value regression modeling implemented with the R packages pseudo and geepack.^35^

Next, we investigated the association between the clinical groups and baseline amyloid (A±) and tau pathology (T±) using logistic regressions and with the combined biomarker profiles (i.e., A‐T‐, A+T‐, A‐T+, A+T+) using multinomial logistic regressions (reference category: A‐T‐).

Then, we examined the association between the clinical groups and clinical progression in amyloid‐positive individuals, again using Cox and RMST analyses.

To obtain estimates for 5‐year symptom progression, we calculated the sensitivity, specificity, and predictive values of SCD and MNPD for the progression to MCI within this timeframe using inverse probability of censoring weighting as implemented in the R package timeROC.^36^ Using the same method and metrics, we investigated the prognostic value of baseline amyloid positivity for the 5‐year progression to MCI in the different clinical groups.

Finally, we simulated the sample size requirements per arm for clinical trials in the different clinical groups. Based on the progression rates in amyloid‐positive participants, we estimated the sample sizes needed to detect a 30% reduction^37^, ^38^ in the risk of progressing to MCI within 4.5 years,^39^ with 80% power and *α* = 0.05, using the R package powerSurvEpi. Based on the rates of amyloid positivity in our sample, we also estimated the number of screenings for amyloid pathology required in each group to reach the necessary sample sizes per arm.

Statistical analyses were conducted with R, version 4.4.1. They included all participants with data in all variables of a given analysis. Analyses were conducted in pooled cross‐cohort samples as well as the separate cohorts. All *p* values were two‐sided and considered significant at *p* < 0.05. All regression models were adjusted for age, sex/gender (0 = male, 1 = female), and cohort. Cox and RMST regressions were additionally adjusted for years of education. In sensitivity analyses in ADNI and NACC, we additionally adjusted for race and Hispanic ethnicity. This data was not available in DELCODE. Moreover, we ran sensitivity analyses that adjusted for the source of biomarker information (PET or CSF) as well as analyses adjusting for apolipoprotein E (APOE) ε4 genotype positivity and vascular risk factors including body mass index (BMI), smoking, hypertension, hypercholesterolemia, and diabetes.

## 3 RESULTS

### 3.1 Associations with clinical progression

The clinical progression analyses included 13,192 participants from three studies (Table 1; Tables S2–S8). Over 10 years, participants with baseline SCD (SCD+) had higher risks for MCI (hazard ratio (HR) [95% confidence interval] = 1.98[1.79^–^2.19], *p *< 0.001) and dementia (HR = 2.12[1.82–2.47], *p *< 0.001) compared to those without SCD (SCD‐). Additionally, participants with MNPD (MNPD+) had higher risks for MCI (HR = 3.16[2.81–3.56], *p *< 0.001) and dementia (HR = 1.97[1.61–2.41], *p *< 0.001) compared to those without MNPD (MNPD‐). These effects were similar in all studies, supporting the cross‐cohort applicability of the MNPD criterion (Table S9).

**TABLE 1:** Participant characteristics.

	SCD‐ / MNPD‐	SCD‐ / MNPD+	SCD+ / MNPD‐	SCD+ / MNPD+
**Participants with progression data**				
N	8360	937	3426	469
Age (years), M (SD)	71.0 (8.4)	71.1 (9.0)	71.0 (8.2)	70.7 (8.8)
Education (years), M (SD)	16.0 (2.6)	16.3 (2.7)	15.9 (2.7)	16.0 (2.8)
Sex (female), N (%)	5409 (64.7%)	558 (59.6%)	2091 (61.0%)	301 (64.2%)
CDR‐SOB, M (SD)	0.0 (0.2)	0.1 (0.2)	0.3 (0.6)	0.5 (0.7)
Follow‐up (years), M (SD)	5.4 (3.3)	4.8 (3.1)	5.1 (3.1)	4.3 (2.8)
Progression to MCI, N (%)^a^	752 (9.3%)	201 (22.2%)	558 (16.9%)	155 (34.0%)
Progression to dementia, N (%)^b^	351 (4.2%)	61 (6.5%)	231 (6.7%)	53 (11.3%)
**Participants with biomarker data**				
N	1608	125	786	97
Age (years), M (SD)	70.6 (7.1)	69.2 (6.8)	71.4 (7.2)	71.2 (7.3)
Education (years), M (SD)	16.4 (2.4)	16.4 (2.5)	16.1 (2.8)	16.3 (2.7)
Sex (female), N (%)	979 (60.9%)	60 (48.0%)	421 (53.6%)	54 (55.7%)
CDR‐SOB, M (SD)	0.1 (0.3)	0.1 (0.4)	0.3 (0.6)	0.5 (0.7)
Amyloid positivity, N (%)^c^	342 (21.3%)	25 (20.0%)	245 (31.2%)	32 (33.3%)
Tau positivity, N (%)^d^	156 (14.8%)	7 (9.9%)	93 (17.0%)	16 (25.8%)
AT biomarker classification^e^				
A‐T‐, N (%)	735 (69.9%)	57 (80.3%)	345 (63.1%)	37 (60.7%)
A+T‐, N (%)	160 (15.2%)	7 (9.9%)	109 (19.9%)	8 (13.1%)
A‐T+, N (%)	84 (8.0%)	2 (2.8%)	33 (6.0%)	3 (4.9%)
A+T+, N (%)	72 (6.9%)	5 (7.0%)	60 (11.0%)	13 (21.3%)
Follow‐up (years), M (SD)	3.1 (2.9)	2.1 (2.5)	3.5 (2.9)	2.9 (2.5)
Progression to MCI, N (%)^f^	70 (6.0%)	14 (17.9%)	98 (16.0%)	31 (44.9%)
Progression to dementia, N (%)^g^	24 (2.0%)	1 (1.2%)	21 (3.3%)	7 (10.0%)

*Note*: Years of follow‐up were truncated at 10 years (maximum follow‐up in regression analyses).

Abbreviations: A, amyloid positivity (present +, absent ‐); CDR‐SOB, Clinical Dementia Rating—Sum of^b^ Boxes; MCI, mild cognitive impairment; MNPD, minor neuropsychological deficits (present +, absent ‐); SCD, subjective cognitive decline (present +, absent ‐); SD, standard deviation; T, tau positivity (present +, absent ‐).

^a^ Data missing in 422 individuals (n = 258 SCD‐/MNPD‐; n = 32 SCD‐/MNPD+; n = 119 SCD+/MNPD‐; n = 13 SCD+/MNPD+)

^b^ Data missing in 2 individuals (n = 2 SCD+/MNPD‐)

^c^ Data missing in 4 individuals (n = 2 SCD‐/MNPD‐; n = 1 SCD+/MNPD‐; n = 1 SCD+/MNPD+)

^d^ Data missing in 882 individuals (n = 555 SCD‐/MNPD‐; n = 54 SCD‐/MNPD+; n = 238 SCD+/MNPD‐; n = 35 SCD+/MNPD+)

^e^ Data missing in 886 individuals (n = 557 SCD‐/MNPD‐; n = 54 SCD‐/MNPD+; n = 239 SCD+/MNPD‐; n = 36 SCD+/MNPD+)

^f^ Data missing in 691 individuals (n = 444 SCD‐/MNPD‐; n = 47 SCD‐/MNPD+; n = 172 SCD+/MNPD‐; n = 28 SCD+/MNPD+)

^g^ Data missing in 593 individuals (n = 382 SCD‐/MNPD‐; n = 43 SCD‐/MNPD+; n = 141 SCD+/MNPD‐; n = 27 SCD+/MNPD+)

**FIGURE 1 alz71458-fig-0001:**
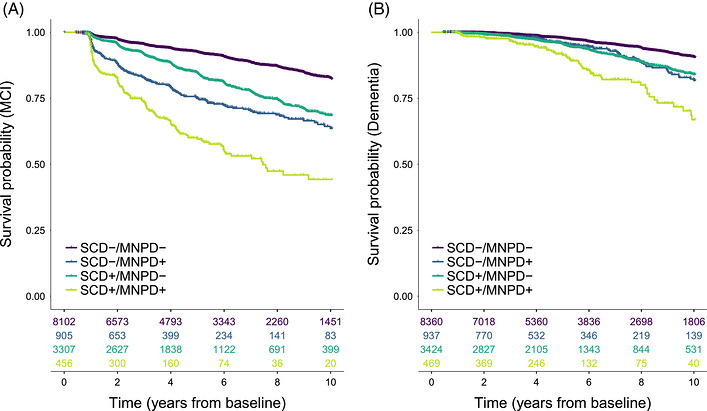
Kaplan–Meier survival curves for the progression to MCI (A) and dementia (B) in all participants with clinical follow‐up data. Risk tables showing the number of participants at risk for MCI (A) or dementia (B) at each time point are displayed below the *x*‐axes. The colored lines in the figure legend indicate which groups correspond to which colors. MCI, mild cognitive impairment; MNPD, minor neuropsychological deficits (present +, absent ‐); SCD, subjective cognitive decline (present +, absent −).

Compared to the SCD‐/MNPD‐reference group, SCD‐/MNPD+ (HR = 3.13[2.68–3.66], *p *< 0.001), SCD+/MNPD‐ (HR = 1.97[1.76–2.20], *p *< 0.001), and SCD+/MNPD+ (HR = 6.23[5.23–7.42], *p *< 0.001) had a higher risk of MCI (Figure 1). Additionally, SCD‐/MNPD+ (HR = 1.76[1.34–2.31], *p *< 0.001), SCD+/MNPD‐ (HR = 2.04[1.72–2.41], *p *< 0.001), and SCD+/MNPD+ (HR = 4.57[3.41–6.10], *p *< 0.001) had a higher risk of dementia than SCD‐/MNPD‐.

Progression to MCI occurred on average 1.39[1.14–1.64], 0.70[0.58–0.82], and 2.55[2.17–2.94] years earlier in SCD‐/MNPD+, SCD+/MNPD‐, and SCD+/MNPD+ compared to SCD‐/MNPD‐ (Table 2). Progression to dementia occurred on average 0.24[0.11–0.37], 0.30[0.22–0.38], and 0.75[0.51–0.99] years earlier in SCD‐/MNPD+, SCD+/MNPD‐, and SCD+/MNPD+ compared to SCD‐/MNPD‐.

**TABLE 2 alz71458-tbl-0002:** Restricted mean survival time (RMST) group differences.

	Unadjusted	Covariate‐adjusted regression		
All participants	RMST [95% CI]	*β* [95% CI]	Wald	*p*‐value
**Outcome: MCI**				
SCD‐ / MNPD‐	9.20 [9.15, 9.26]	NA	NA	NA
SCD‐ / MNPD+	7.80 [7.54, 8.06]	−1.39 [‐1.64, ‐1.14]	119.76	<0.001
SCD+ / MNPD‐	8.45 [8.33, 8.56]	−0.70 [‐0.82, ‐0.58]	121.98	<0.001
SCD+ / MNPD+	6.44 [6.02, 6.86]	−2.55 [‐2.94, ‐2.17]	170.59	<0.001
**Outcome: Dementia**				
SCD‐ / MNPD‐	9.69 [9.65, 9.72]	NA	NA	NA
SCD‐ / MNPD+	9.43 [9.29, 9.57]	−0.24 [‐0.37, ‐0.11]	12.88	<0.001
SCD+ / MNPD‐	9.41 [9.34, 9.49]	−0.30 [‐0.38, ‐0.22]	53.11	<0.001
SCD+ / MNPD+	8.85 [8.56, 9.13]	−0.75 [‐0.99, ‐0.51]	37.37	<0.001

Amyloid‐positive participants	RMST [95% CI]	*β* [95% CI]	Wald	*p*‐value
**Outcome: MCI**				
SCD‐ / MNPD‐	8.47 [7.97, 8.97]	NA	NA	NA
SCD‐ / MNPD+	5.73 [3.50, 7.95]	−2.60 [‐5.05, ‐0.15]	4.34	0.04
SCD+ / MNPD‐	7.01 [6.40, 7.62]	−1.29 [‐2.15, ‐0.43]	8.65	0.003
SCD+ / MNPD+	3.02 [2.29, 3.74]	−5.89 [‐7.63, ‐4.15]	44.02	<0.001
**Outcome: Dementia**				
SCD‐ / MNPD‐	9.29 [8.93, 9.65]	NA	NA	NA
SCD‐ / MNPD+	10 [10.00, 10.00]	0.85 [‐0.16, 1.85]	2.73	0.098
SCD+ / MNPD‐	8.88 [8.39, 9.38]	−0.49 [‐1.12, 0.14]	2.25	0.13
SCD+ / MNPD+	6.20 [5.28, 7.13]	−3.51 [‐5.98, ‐1.04]	7.78	0.005

*Note*: The unadjusted results show the raw RMST—the average time until the progression to MCI/dementia—over 10 years of follow‐up. The covariate adjusted columns show the results of the pseudo‐value regression analyses. These estimated the RMST differences between the SCD‐/MNPD‐ reference group and the SCD‐/MNPD+, SCD+/MNPD‐, and SCD+/MNPD+ groups. The regression analyses were adjusted for baseline age, years of education, sex/gender, and study cohort. No dementia conversions were observed in the amyloid‐positive SCD‐/MNPD+ group.

Abbreviations: CI, confidence interval; MCI, mild cognitive impairment; MNPD, minor neuropsychological deficits (present +, absent ‐); NA, not available; RMST, restricted mean survival time; SCD, subjective cognitive decline (present +, absent ‐).

**FIGURE 2 alz71458-fig-0002:**
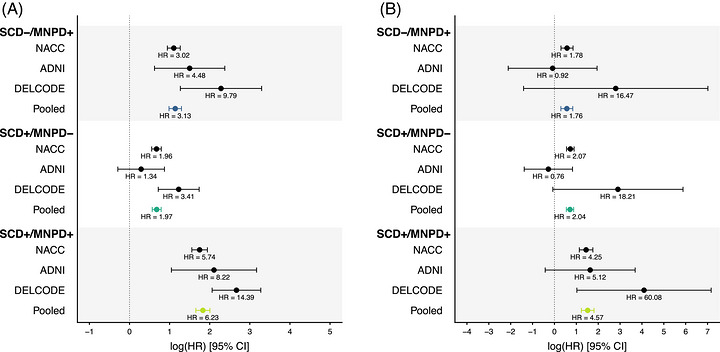
Forest plots displaying age‐, sex‐, and education‐adjusted Cox regression results for the progression to MCI (A) and dementia (B) in all participants with clinical follow‐up data. Models in the pooled analyses were additionally adjusted for the cohorts. Cox regression models with firth's penalized maximum likelihood estimation were used for the dementia progression analyses in DELCODE due to the absence of dementia conversions in the SCD‐/MNPD‐ reference group. ADNI, Alzheimer's Disease Neuroimaging Initiative; DELCODE, German Center for Neurodegenerative Diseases Longitudinal Cognitive Impairment and Dementia study; MCI, mild cognitive impairment; MNPD, minor neuropsychological deficits (present +, absent ‐); NACC, National Alzheimer's Coordinating Center; SCD, subjective cognitive decline (present +, absent −).

Results from the individual studies are listed in the Supplement (Tables S10–S11) and individual Cox regression results are displayed in Figure 2. Associations with the progression to MCI were consistent across the cohorts, although the effect of SCD+/MNPD‐ was smaller in ADNI compared to the other studies. Associations with the progression to dementia differed more widely across cohorts and showed higher statistical uncertainty (Figure 2). Adjustments for race, Hispanic ethnicity, vascular risk factors, and APOE did not change the results (Tables S12–S17).

### 3.2 Associations with AD biomarkers

**FIGURE 3:**
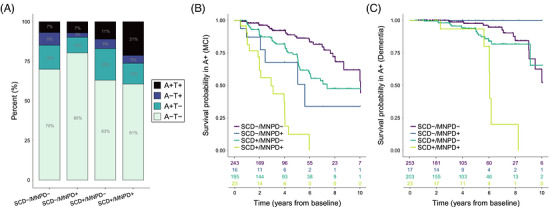
A) Stacked bar plots showing the proportion of the four A/T biomarker profiles in each of the four clinical groups in the sample of participants with amyloid and tau data at baseline. (B, C) Kaplan–Meier survival curves for the progression to MCI (B) and dementia (C) in amyloid‐positive participants with clinical follow‐up data. Risk tables showing the number of participants at risk for MCI (B) or dementia (C) at each time point are displayed below the *x*‐axes. The colored lines in the figure legend indicate which groups correspond to which colors. A, amyloid positivity (present +, absent ‐); MCI, mild cognitive impairment; MNPD, minor neuropsychological deficits (present +, absent ‐); SCD, subjective cognitive decline (present +, absent ‐); T, tau positivity (present +, absent −).

Compared to SCD‐/MNPD‐, SCD+/MNPD‐ (odds ratio (OR) [95% confidence interval] = 1.47[1.20–1.81], *p *< 0.001) and SCD+/MNPD+ (OR = 1.64[1.04–2.59], *p *= 0.03) had increased odds of amyloid[Fig alz71458-fig-0001], [Table alz71458-tbl-0002] pathology (*n* = 2,616; Table 1; Tables S18–S19). The odds of tau pathology were increased in SCD+/MNPD+ (OR = 2.10[1.13–3.90], *p *= 0.02). In the multinomial logistic regressions, SCD+/MNPD‐ (OR = 1.62[1.09–2.39], *p *= 0.02) and SCD+/MNPD+ (OR = 3.19[1.56–6.56], *p *= 0.002) had higher odds for the A+T+ biomarker profile than SCD‐/MNPD‐ (Figure 3). Results from the individual studies are listed in the Supplement (Tables S18–S19). Associations with AD biomarkers tended to be lower in NACC compared to the other cohorts. Adjustments for race, Hispanic ethnicity, type of biomarker data, or vascular risk factors did not change the results (Tables S20–S28). Adjustment for APOE decreased the association between SCD+/MNPD+ and the odds for amyloid and tau pathology as well as the A+/T+ biomarker profile (Tables S26–S28).

### 3.3 Associations with clinical progression in amyloid‐positive participants

In amyloid‐positive individuals (*n* = 497,[Fig alz71458-fig-0002] Figure 3, Tables S10–S11), SCD‐/MNPD+ (HR = 4.99[2.03–12.26], *p* < 0.001), SCD+/MNPD‐ (HR = 2.36[1.47–3.80], *p* < 0.001), and SCD+/MNPD+ (HR = 11.70[5.94–23.04], *p* < 0.001) had a higher risk of MCI than SCD‐/MNPD‐. The risk of dementia was increased in SCD+/MNPD+ (Firth penalized HR = 9.07[2.91–28.29], *p* < 0.001).

Progression to MCI occurred on average 2.60[0.15–5.05], 1.29[0.43–2.15], and 5.89[4.15–7.63] years earlier in SCD‐/MNPD+, SCD+/MNPD‐, and SCD+/MNPD+ compared to SCD‐/MNPD‐ (Table 2). Progression to dementia occurred on average 3.51[1.04–5.98] years earlier in SCD+/MNPD+ compared to SCD‐/MNPD‐.

**FIGURE 4:**
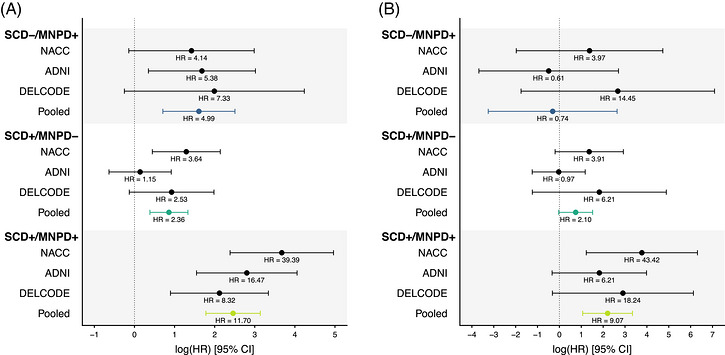
Forest plots displaying age‐, sex‐, and education‐adjusted Cox regression results for the progression to MCI (A) and dementia (B) in amyloid‐positive participants with clinical follow‐up data. Models in the pooled analyses were additionally adjusted for the cohorts. Cox regression models with firth's penalized maximum likelihood estimation were used for the dementia progression analyses due to the absence of dementia conversions in the SCD‐/MNPD+ group and the DELCODE SCD‐/MNPD‐ group. ADNI, Alzheimer's Disease Neuroimaging Initiative; DELCODE, German Center for Neurodegenerative Diseases Longitudinal Cognitive Impairment and Dementia study; MCI, mild cognitive impairment; MNPD, minor neuropsychological deficits (present +, absent ‐); NACC, National Alzheimer's Coordinating Center; SCD, subjective cognitive decline (present +, absent −).

Results from the individual studies are listed in the Supplement (Tables S10–S11) and individual Cox regression results are displayed in Figure 4. Associations with the progression to MCI were consistent across the cohorts, although the effect of SCD+/MNPD‐ was smaller in ADNI compared to the other studies. Associations with the progression to dementia differed more widely across cohorts and showed higher statistical uncertainty (Figure 4). Adjustments for race, Hispanic ethnicity, type of biomarker data, vascular risk factors, and APOE did not change the results (Tables S13–S14, S16–S17, S29–S33).

### 3.4 Prognostic utility for the 5‐year prediction of MCI

**FIGURE 5:**
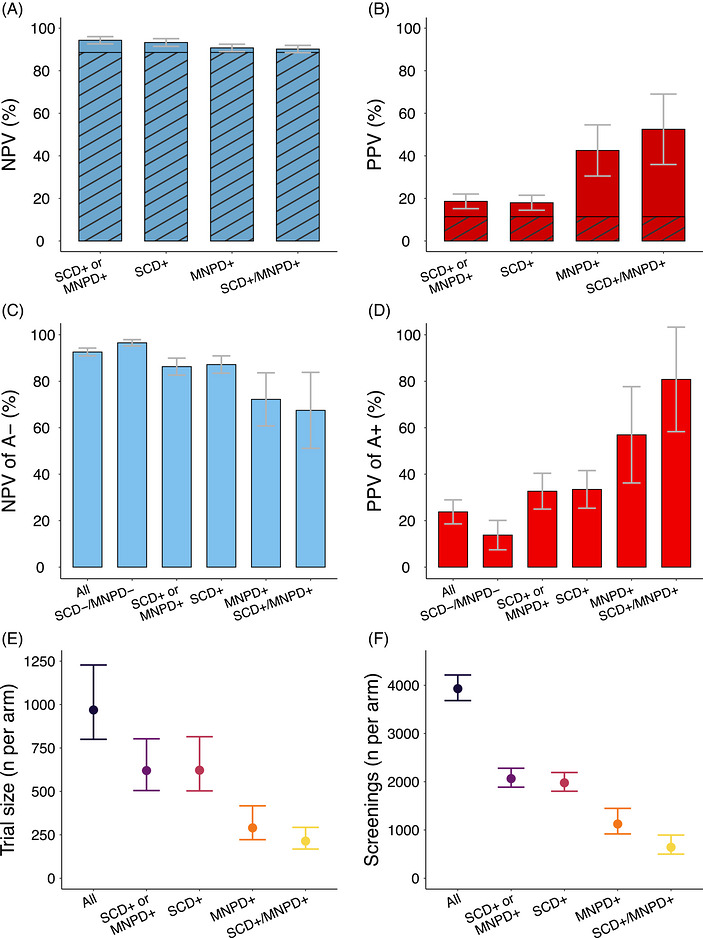
(A, B) Bar plots representing the PPV (A) and NPV (B) of four clinical criteria for the progression to MCI over 5 years of follow‐up. We compared the prognostic value of the presence of any clinical symptom (SCD+ or MNPD+), SCD (SCD+), MNPD (MNPD+), and both SCD and MNPD (SCD+/MNPD+). The hatched patterns show the rate of progression across all clinical groups. (C, D) Bar plots representing the PPV (C) and NPV (D) of amyloid positivity for the progression to MCI over 5 years of follow‐up in the different clinical groups (all clinical groups [All], participants without SCD or MNPD [SCD‐/MNPD‐], participants with SCD or MNPD [SCD+ or MNPD+], participants with SCD [SCD+], participants with MNPD [MNPD+], and participants with SCD and MNPD [SCD+/MNPD+]). (E) Estimated sample sizes per trial arm necessary to detect a 30% reduction in the risk of progression to MCI over 4.5 years of follow‐up across different clinical inclusion criteria (all clinical groups [All], participants with SCD or MNPD [SCD+ or MNPD+], participants with SCD [SCD+], participants with MNPD [MNPD+], and participants with SCD and MNPD [SCD+/MNPD+]). (F) Estimated number of biomarker screenings necessary to reach the sample sizes estimated in Figure 3E. These estimates were calculated by dividing the estimated trial sizes per arm in Figure 3E by the proportion of amyloid‐positive participants in each clinical group. MCI, mild cognitive impairment; NPV, negative predictive value; MNPD, minor neuropsychological deficits (present +, absent ‐); SCD, subjective cognitive decline (present +, absent ‐); PPV, positive predictive value.

Across all participants with baseline amyloid information and clinical progression data (*n* = 1,924), the rate of conversion to MCI within 5 years was 11%. Negative predictive values (NPVs) were high (90%–94%) across all clinical groups (Figure 5 A–D, Table S34, Figures S4–S5). The positive predictive value (PPV) was 19%[15%–22%] for participants with SCD and/or MNPD, 18%[14%–22%] for SCD+ regardless of MNPD, and 43%[31%–55%] for MNPD+ regardless of SCD. SCD+/MNPD+ showed the highest PPV (53%[36%–69%]).

In the complete sample, the sensitivity of amyloid positivity for the progression to MCI within 5 years was 51%[43%–59%], and the specificity was 79%[76%–82%]. These values were similar across all clinical groups (Table S34). Across all participants, the PPV of amyloid positivity was 24%[19%–29%]. It increased to 33%[25%–40%] in participants with SCD and/or MNPD and to 34%[25%–42%] in those with SCD regardless of MNPD. It increased further in participants with MNPD regardless of SCD (57%[63%–78%]) and in SCD+/MNPD+ participants (81%[58%–103%]). Conversely, the NPV of amyloid negativity was 93%[91%–94%] across all clinical groups, 86%[83%–90%] in participants with SCD and/or MNPD, 87%[84%–91%] in SCD+ regardless of MNPD, 72%[61%–84%] in MNDP+ regardless of SCD, and 68%[51%–84%] in SCD+/MNPD+.

### 3.5 Sample size simulation for clinical trials and amyloid screenings

In clinical trials with amyloid‐positive, cognitively unimpaired participants, recruiting participants with SCD and/or MNPD or those with SCD regardless of MNPD reduces the sample size per trial arm from 969[800–1228] across all clinical groups to 620[505–803] and 622[503–815], respectively (Figure 5 E–F; Table S35). Recruiting individuals with MNPD regardless of SCD reduced the estimated sample size to 290[222–417], and recruiting only SCD+/MNPD+ reduced it to 214[168–293].

The number of amyloid measurements required to achieve the estimated sample sizes per arm could be reduced from 3930[3683–4213] without symptomatic screening to 2065[1887–2280] in those with SCD and/or MNPD, to 1978[1803–2192] in those with SCD regardless of MNPD, to 1124[919–1448] in those with MNPD regardless of SCD, and to 642[500–895] in SCD+/MNPD+ (Table S35).

## 4 DISCUSSION

### 4.1 Summary and discussion of key findings

In this large multi‐sample study of CN participants, we showed that individuals with SCD and/or MNPD—especially those with both—have an increased risk of and decreased time until progression to MCI and dementia. SCD+/MNPD‐ and SCD+/MNPD+ participants additionally had higher rates of AD pathology. In amyloid‐positive SCD+/MNPD+ participants, the hazard of MCI and dementia over 10 years compared to amyloid‐positive SCD‐/MNPD‐ participants increased almost 12‐fold and 9‐fold, respectively. Furthermore, the PPV of amyloid pathology for the prediction of MCI more than tripled in SCD+/MNPD+ compared to the entire sample. Additionally, shifting recruitment strategies for clinical trials in preclinical AD from CN to this subgroup could reduce sample size requirements by up to three quarters.

Our results align with previous studies in CN cohorts showing that SCD^7^, ^8^, ^9^, ^40^, ^41^ and MNPD^12^, ^13^, ^14^, ^15^, ^42^, ^43^, defined by varying criteria,^44^ are associated with AD pathology and clinical progression. We provide novel evidence on their combined prognostic significance and practical relevance, including in amyloid‐positive individuals. SCD+/MNPD+ individuals consistently had the highest progression risk and proportion of AD pathology, while SCD‐/MNPD+ and SCD+/MNPD‐ participants had intermediate progression risks. MNPD in the absence of SCD was not associated with AD pathology. These results suggest that SCD and MNPD provide complementary information. The presence of SCD may indicate that MNPD results from longitudinal decline, consistent with early AD pathology, while MNPD by itself may result from other disease pathways or indicate lifelong below‐average cognitive abilities. Conversely, MNPD provides objective support for the subjective concerns of individuals with SCD.

Consistent with this reasoning, MNPD can enhance clinical staging in AD. We found that amyloid‐positive individuals with SCD, that is individuals in stage 2, were much closer in time to the progression to MCI when they had MNPD, with an average progression time of 3.02[2.29–3.74] years compared to 7.01[6.40–7.62] in those with SCD but without MNPD. These data show that the co‐occurrence of SCD and MNPD indicates a particularly high‐risk group within AD stage 2.

This highly elevated risk of clinical progression in SCD+/MNPD+ participants could be observed in each individual cohort, indicating that the high‐risk status of this group persists across context factors such as countries and recruitment settings. For the progression to MCI, results were also largely consistent across the cohorts in the SCD‐/MNPD+ and SCD+/MNPD‐ groups. The effect of SCD+/MNPD‐ was smaller in ADNI compared to the other cohorts, likely in part due to the weaker association between SCD and clinical progression in volunteer‐based recruitment settings.^40^, ^45^ Results for the progression to dementia followed a similar pattern in each cohort but showed more heterogeneity and higher statistical uncertainty in the individual studies due to the low frequency of dementia conversions in cognitively normal individuals during the available follow‐up period. In addition, AD biomarker associations tended to be attenuated in NACC compared to ADNI and DELCODE, possibly due to the lack of a single, standardized biomarker assessment protocol across the different NACC study centers.

### 4.2 Implications for risk prediction and clinical trials

Focusing on AD stage 2 and MNPD could facilitate the translation of early treatments into healthcare. Amyloid‐targeting antibodies^37^, ^38^ will likely not be authorized for broad preventative use among all amyloid‐positive CN in most healthcare systems. In particular, the cost‐benefit‐risk ratio of anti‐amyloid treatments may be insufficient in asymptomatic amyloid‐positive individuals in AD stage 1, due to their low risk of short‐term progression. In our study, the majority of amyloid‐positive CN without MNPD and SCD did not develop MCI within 5 years (14%[8%–20%] PPV of amyloid positivity). However, in patients in AD stage 2, who seek medical help due to their subtle symptoms and ask for medication, these treatments could be justified—especially among high‐risk individuals who also have MNPD.

Accordingly, our power calculations suggest the consideration of subtle symptoms in preclinical AD could mitigate the risk of costly, unsuccessful clinical trials in preclinical AD, caused by a lack of cognitive decline in placebo groups, and reduce amyloid screening failure rates. Based on our data, a clinical trial with amyloid‐positive individuals with SCD would need only 64% of the sample size and 50% of the biomarker screenings required for a trial in preclinical AD without symptomatic selection criteria. A trial with amyloid‐positive SCD+/MNPD+ participants could even reduce these figures to 22% and 16%, respectively, compared to a trial with any amyloid‐positive CN. These findings illustrate how subtle symptoms can enable trials in preclinical AD using the clinically relevant progression to MCI as the outcome measure. Importantly, the inclusion of SCD and MNPD in the recruitment for clinical trials in preclinical AD would require no additional resources, since cognitive assessments are already required to rule out MCI. The recruitment of individuals with MNPD and SCD could be further empowered by remote digital prescreening or cognitive screening in primary care.^46^

Finally, our analyses show that preclinical cognitive phenotyping modifies the prognostic value that can be obtained from amyloid biomarker testing. In asymptomatic individuals, a negative amyloid test result is linked to a very low 5‐year risk of MCI (NPV = 93%[91%–94%]), while a positive result alone does not indicate a high risk for progression (PPV = 14%[8%–20%]). In contrast, in SCD+/MNPD+, a positive test result becomes substantially more informative for the 5‐year risk of MCI (PPV = 81%[58%–103%]). However, the NPV of amyloid testing is lower (68%[51%–84%]), highlighting the need for the assessment of further causes of these symptoms. These substantial discrepancies in prognostic values should be taken into account before the conduction and disclosure of biomarker assessments in CN. These considerations are also highly relevant for the recently proposed Brain Health Service initiative, which develops conceptual and practical approaches for secondary dementia prevention and aims to develop individualized, multi‐variate risk estimates for use in risk counseling.^17^, ^18^ We propose that SCD and MNPD should be incorporated into these risk calculations. These symptoms not only enable the identification of individuals with an increased risk of clinical progression, but do so beyond modifiable risk factors such as BMI, hypertension, hypercholesterolemia, and diabetes.

Importantly, the median‐based MNPD criterion used here does not require specific cognitive tests and showed consistent associations with progression across several studies, fulfilling a key prerequisite for wider implementation in clinical trials and risk counseling. However, varying MNPD criteria have been proposed and may differ in their prognostic utility.^44^ This should be taken into account in the context of any practical implementation of MNPD.

### 4.3 Limitations and future directions

Our study has limitations. The operationalization of some key variables, particularly SCD and AD pathology, varied between the included studies. The assessment of SCD in NACC and ADNI‐1 relied on only one item and was memory‐specific, while the other cohorts assessed concerns in several cognitive domains. This heterogeneity potentially lead to an underestimation of the association between SCD and the study outcomes and limits the generalizability of our findings to self‐perceived changes in non‐memory domains. In NACC, biomarker assessments, relying on local study center procedures, were especially heterogeneous, and CSF tau data records reflected either p‐tau or total tau. However, biomarker results remained consistent after excluding NACC participants (Tables S18–S19). In ADNI, we used CSF Aβ42 to measure amyloid pathology, instead of the Aβ42/40 ratio,^47^ because Aβ40 was not consistently available. Finally, the generalizability of our findings is limited by the insufficient diversity of our sample.

Future studies should examine the additional diagnostic and prognostic value of neurobehavioral symptoms beyond the cognitive symptoms investigated here, since they can also be early manifestations of preclinical AD and are recognized as symptoms of AD stage 2 in current diagnostic guidelines.^3^ Moreover, more research is needed to understand the combined prognostic value of subtle clinical symptoms and AD biomarkers, particularly tau PET‐based AD staging, in CN populations. Finally, future studies should systematically compare different MNPD operationalizations and cognitive performance thresholds to ideally suggest a harmonized assessment standard for the still heterogeneous field of MNPD research.^44^

## 5 CONCLUSIONS

In conclusion, our data highlights the importance of refining the concept of preclinical AD. Not considering subtle symptoms like SCD and MNPD increases the costs and failure risks of clinical trials. These symptoms are also relevant for risk counseling and the decision to conduct biomarker assessments, as well as the interpretation of biomarker test results, particularly in light of the increasing availability of plasma biomarkers. In the future, they may also become important for the decision to prescribe preventative treatments for preclinical AD.

## CONFLICT OF INTEREST STATEMENT

Claudia Bartels has received honoraria as a diagnostic consultant for Boehringer Ingelheim, as a commercial advisory board member for Lilly, and for lectures from Boehringer Ingelheim, Roche, Lilly, and Eisai. Claudia Bartels has received funding from the German Alzheimer Association. Henning Boecker has received honoraria for expert testimony provided to the Dementia Research Switzerland—Synapsis Foundation. Katharina Buerger has received honoraria from Eisai, Lilly, and Roche and travel grants from Lilly and Novo Nordisk. Alexander Drzezga reports the following disclosures. Research Support: Siemens Healthineers, Life Molecular Imaging, GE Healthcare, AVID Radiopharmaceuticals, Sofie, Eisai, Novartis/AAA, Ariceum Therapeutics; Speaker Honoraria / Advisory Boards: Siemens Healthineers, Sanofi, GE Healthcare, Biogen, Novo Nordisk, Invicro, Novartis/AAA, Bayer Vital, Lilly, Peer View Institute for Medical Education, International Atomic Energy Agency; Stock: Siemens Healthineers, Lantheus Holding, Lilly; Patent for 18F‐JK‐PSMA‐7 (Patent No.: EP3765097A1; Date of patent: January 20, 2021); Grants: DFG Grants SFB 1451 C04, DR 445/9‐1. Klaus Fliessbach has received honoraria from the RG Gesellschaft für Information und Organisation mbH. Franziska Maier has received honoraria from Novo Nordisk and a travel grant from Johnson & Johnson. Franziska Maier is an advisory board member for Johnson & Johnson and Lilly. Robert Perneczky has received grants from Roche. Robert Perneczky has received consulting fees and honoraria from Roche, Biogen, GSK, BMS, Schwabe, Tabuk, Johnson & Johnson, Eisai, and Eli Lilly. Robert Perneczky holds stock in Medotrax GmbH and Vistim Labs Ltd. Oliver Peters has received consulting fees from Biogen, Eisai, Grifols, Noselab, Novo Nordisk, Prinnovation, and Roche and honoraria from Eisai, Lilly, and Roche. Oliver Peters is an advisory board member for Eisai, Grifols, Neurimmune, Noselab, Roche, and Novartis. Oliver Peters is a board member of the German Dementia Competence Network and the Hirnliga. Josef Priller reports the following disclosures. Travel Grant: EHDN/CHDI. Patents: EPO variantsSinapps2. Advisory Board: Sinapps2. Board/Society Membership: DGPPN, DGGPP, DGBP, DZNE, UK DRI, and DZPG. Axel Rominger has received grants from Novartis, Siemens Healthineers, and the Swiss National Science Foundation and honoraria from Siemens Healthineers. Anja Schneider has received grants from BMBF, BMG, tALS, and the Schick Foundation and research support from Vectory. Anja Schneider is an advisory board member for Biogen and CELIA and a board/society member of the DGBP. Eike Jakob Spruth has received honoraria from the Alzheimer Gesellschaft Berlin e.V. Stefan Teipe has received honoraria from Thieme, RG Ärztefortbildung GmbH, Lilly, and Eisai. Stefan Teipe reports data monitoring committee membership for the Biogen ENVISION study and advisory board memberships for Roche, Biogen, Grifols, Eisai, Lilly, and GE Healthcare. Jens Wiltfang has received consulting fees from Immungenetics, Noselab, and Roboscreen and honoraria from Beeijing Yibai Science and Technology Ltd., Gloryren, Janssen Cilag, Pfizer, Med Update GmbH, Roche, and Lilly. Jens Wiltfang reports the following patents: PCT/EP 2011 001724, PCT/EP 2015 052945. Jens Wiltfang reports advisory board memberships for Biogen, Abbott, Boehringer Ingelheim, Lilly, MSD Sharp & Dohme, and Roche. Jens Wiltfang reports board/society memberhsips with AGNP, DGLN, DGPPN, Hirnliga, and CSF‐Society. Emrah Düze has received consulting fees and honoraria from Lilly Roche, and Eisai. Emrah Düze is a co‐founder and holds stock options in neotiv GmbH. Frank Jessen has received fees for advice and presentations from AC immune, Biogen, Cogthera, Eisai, Eli Lilly, Grifols, Janssen, Novo Nordisk, and Roche. The remaining authors report no conflicts of interest relevant to this work. Author disclosures are available in the supporting information.

## CONSENT

All participants provided written informed consent.

## Supporting information



Supporting Information

Supporting Information
