# Supplementary material for: Minor neuropsychological deficits and stage 2 of Alzheimer's disease
Source: Alzheimers Dement. 2026 May 13;22(5):e71458. doi: 10.1002/alz.71458 (PMC13171462; doi:10.1002/alz.71458)
Supplement: Supplementary file 2 — Supporting Information [file ALZ-22-e71458-s002.pdf]

# Supplementary Material

**Supplementary Methods**

**Figure S1.** Flow diagram of the sample selection in ADNI

**Figure S2.** Flow diagram of the sample selection in DELCODE

**Figure S3**. Flow diagram of the sample selection in NACC

**Figure S4**. Sensitivity, specificity, positive predictive value curves over five years of follow-up (Predictive value of clinical groups)

**Figure S5**. Sensitivity, specificity, positive predictive value curves over five years of follow-up (Predictive value of amyloid positivity in different clinical groups)

**Table S1**. Neuropsychological assessment batteries

**Table S2**. Baseline characteristics of participants with baseline amyloid and progression data

**Table S3**. Baseline characteristics of participants with progression data and participants with biomarker data (ADNI)

**Table S4**. Baseline characteristics of participants with baseline amyloid and progression data (ADNI)

**Table S5**. Baseline characteristics of participants with progression data and participants with biomarker data (DELCODE)

**Table S6**. Baseline characteristics of participants with baseline amyloid and progression data (DELCODE)

**Table S7**. Baseline characteristics of participants with progression data and participants with biomarker data (NACC)

**Table S8**. Baseline characteristics of participants with baseline amyloid and progression data (NACC)

**Table S9**. Cox regression results for the main effects of SCD and MNPD (Pooled samples and separate cohorts)

**Table S10**. Cox regression results (Pooled samples and separate cohorts)

**Table S11**. Pseudo-value regression results (Pooled samples and separate cohorts)

**Table S12**. Baseline characteristics of participants with progression data (Participants with data on race and ethnicity)

**Table S13**. Cox regression results (without / with adjustment for race and ethnicity)

**Table S14**. Pseudo-value regression results (without / with adjustment for race and ethnicity)

**Table S15**. Baseline characteristics of participants with progression data (Participants with data on vascular risk factors and APOE)

**Table S16**. Cox regression results (without / with adjustment for vascular risk factors and APOE)

**Table S17**. Pseudo-value regression results (without / with adjustment for vascular risk factors and APOE)

**Table S18**. Logistic regression results (Pooled samples and separate cohorts)

**Table S19**. Multinomial logistic regression results (Pooled samples and separate cohorts)

**Table S20**. Baseline characteristics of participants with biomarker data (Participants with data on race and ethnicity)

**Table S21**. Logistic regression results (without / with adjustment for race and ethnicity)

**Table S22**. Multinomial logistic regression results (without / with adjustment for race and ethnicity)

**Table S23**. Baseline characteristics of participants with biomarker data (Including biomarker type)

**Table S24**. Logistic regression results (without / with adjustment for biomarker type)

**Table S25**. Multinomial logistic regression results (without / with adjustment for biomarker type)

**Table S26**. Baseline characteristics of participants with biomarker data (Participants with data on vascular risk factors and APOE)

**Table S27**. Logistic regression results (without / with adjustment for vascular risk factors and APOE)

**Table S28**. Multinomial logistic regression results (without / with adjustment for vascular risk factors and APOE)

**Table S29**. Baseline characteristics of amyloid-positive participants with progression data (Participants with data on race and ethnicity)

**Table S30**. Baseline characteristics of amyloid-positive participants with progression data (Including biomarker type)

**Table S31**. Cox regression results (without / with adjustment for biomarker type)

**Table S32**. Pseudo-value regression results (without / with adjustment for biomarker type)

**Table S33**. Baseline characteristics of amyloid-positive participants with progression data (Participants with data on vascular risk factors and APOE)

**Table S34**. Sensitivity, specificity, and predictive values for the progression to MCI within five years

**Table S35**. Power analysis results

**References**

## Supplementary Methods

## 1 Cohort descriptions

### 1.1 ADNI

The Alzheimer's Disease Neuroimaging Initiative (ADNI) was launched in 2003 as a public-private partnership, led by Principal Investigator Michael W. Weiner, MD. The primary goal of ADNI has been to test whether serial magnetic resonance imaging (MRI), positron emission tomography (PET), other biological markers, and clinical and neuropsychological assessment can be combined to measure the progression of mild cognitive impairment (MCI) and early Alzheimer's disease (AD). The ADNI protocols were reviewed and approved by the Institutional Review Boards (IRBs) of the participating study centers. All participants provided written informed consent prior to inclusion in the study.

The ADNI is an observational, longitudinal cohort study, which is conducted at over 50 sites in the United States of America and Canada. The study was initiated in 2005 and can, currently, be separated into three different study phases – ADNI-1, ADNI-2, and ADNI-3.[1–3] We analyzed ADNI data downloaded in December 2023. All assessment included in the utilized data were conducted between September 7th 2005 and November 14th 2023. Our analyses included cognitively unimpaired participants from 65 study centers and all ADNI phases, including those with subjective cognitive decline (SCD). In ADNI-1 and ADNI-2, cognitively normal participants initially had annual in-clinic study visits and an additional in-clinic assessment 6 months after their baseline. From May 2014, cognitively normal participants had an in-clinic assessment every other year, unless they were classified as diagnostic converters.

Cognitively normal ADNI participants had to meet the following core inclusion criteria: 55-90 years old, fluent in English or Spanish, no memory impairment, defined by education-adjusted cut-offs scores for the Wechlser Memory Scale – Revised (WMS-R) Logical Memory delayed recall subtest, a Mini-Mental Sate Examination (MMSE) score of 24-30, a Clinical Dementia Rating (CDR) global score of 0, and no significant impairment in cognitive functions or activities of daily living. The following exclusion criteria applied: significant neurological disease, evidence of focal lesions on MRI scans, foreign objects in the body (e.g. pacemakers), history of schizophrenia, major depression or bipolar disorder in the past year, psychotic features, agitation, or behavioral problems in the past three months, alcohol or substance abuse or dependence in the past two years, use of psychoactive medications (e.g., neuroleptics, OCD/ADHD medications), use of investigational drugs one month prior to study entry and for the duration of the study, significant systemic illness or unstable medical condition, clinically significant abnormalities of vitamin B12, thyroid function, or rapid plasma reagin tests, residence in a nursing facility, and participation in studies involving cognitive assessments more frequently than annually.

### 1.2 DELCODE

The German Center for Neurodegenerative Diseases (DZNE) Longitudinal Cognitive Impairment and Dementia study (DELCODE) is a longitudinal, observational study conducted at 10 DZNE sites in Germany.[4,5] Participants were enrolled into the study between April 2014 and August 2018. Annual follow-up assessments are ongoing. In this study, we used data from the April 2024 DELCODE data freeze. We analyzed data from assessments conducted between May 8th 2014 and April 12th 2024. The study protocols were reviewed and approved by the IRBs of the participating DZNE sites. The study participants provided written informed consent prior to their inclusion in the study.

We included SCD patients and participants from the DELCODE control group in our analyses. The SCD group consisted of memory clinical patients, who sought medical support due to a self-perceived cognitive decline, but had a normal cognitive profile in their in-clinic neuropsychological assessment. The DELCODE control group was recruited via advertisements and consisted of cognitively normal individuals, who had no subjective cognitive concerns.

Both groups had to meet the following core inclusion criteria: at least 60 years old, fluent in German, and no impairment in the Consortium to Establish a Registry for Alzheimer's Disease (CERAD) neuropsychological assessment battery, defined by a demographically-adjusted performance of z>-1.5 on all CERAD subtests. The following exclusion criteria applied to both groups: conditions interfering with participation in the study (e.g., significant sensory impairment), major depressive episode at baseline, major psychiatric disorders either at baseline or in the past (e.g., psychotic disorder, bipolar, disorder, substance abuse), neurodegenerative disorders, vascular dementia, history of stroke with residual clinical symptoms, history of malignant disease, severe or unstable medical condition, clinically significant abnormalities in vitamin B12, and consumption of prohibited drugs (e.g. chronic use of psychoactive compounds with sedative or anticholinergic effects, use of anti-dementia agents, use of investigational drugs for treatment of cognitive impairment one month prior to study entry and for the duration of the study).

## 1.3 NACC

The National Alzheimer's Coordinating Center (NACC) database consists of longitudinal, observational data collected by Alzheimer's Disease Research Centers (ADRCs) in the United States of America. The assessments and data collection conducted by the ADRCs follow a set of standardized protocols referred to as the Uniform Data Set (UDS). Systematic data collection with the UDS began in 2005. Two revisions of the UDS were later introduced in 2008 and 2015 – the UDS-2 and UDS-3.[6,7]

In this study, we analyzed data from the June 2024 NACC data freeze. All assessments were conducted between June 9th 2005 and May 29th 2024. We included study participants from 46 ADRCs, who were assessed with the UDS-1, UDS-2, or UDS-3 and were classified as cognitively normal or cognitively impaired, but MCI-free, at baseline by study clinicians. The NACC protocols include annual in-clinic or telephone study visits. Only neuropsychological data from in-clinic assessments were used in our analyses.

The study protocols and data sharing procedures were reviewed and approved by the IRBs of the participating ADRCs and the University of Washington IRB. The study participants provided written informed consent prior to their inclusion in the database.

# 2 Clinical and cognitive assessments

## 2.1 ADNI

In ADNI-1, we used the item "Do you feel you have more problems with memory than most?" from the Geriatric Depression Scale (GDS) to measure SCD, because no dedicated SCD questionnaire was included in this study phase. In ADNI-2 and ADNI-3, we used the Everyday Cognition Questionnaire (ECOG) to measure SCD. The cut-off for SCD was set at one SD above the mean ECOG total score of the DELCODE control group, which included cognitively normal individuals without subjective cognitive concerns (M=1.17, SD=0.18; Cut-Off: 1.35).

The core neuropsychological assessment battery remained the same across the three ADNI phases. However, the Boston Naming Test (BNT) was replaced by the Multilingual Naming Test (MINT) in ADNI-3. The neuropsychological tests we analyzed in this study are listed in eTable 1. Raw test scores were converted into age-, sex/gender-, and education-adjusted z-scores based on published norms from the UDS and Mayo normative studies.[8–10] For tests, which were included in both the UDS-1/2 and UDS-3 battery (e.g., Trail Making Test), we used the UDS-2 norms.[8]

For the identification of minor neuropsychological deficits (MNPD), we calculated each participant's median z-score of the nine cognitive measures listed in eTable 1. Incident MCI was identified algorithmically. We assigned the cognitive tests to the domain of Learning and Memory, Language, and Processing Speed and Attention (eTable 1). At least one test with a score of z≤-1.5 and a second test with a score of z≤-1.0 in the same cognitive domain were required to fulfill the criterion for MCI. Only participants, who still met this criterion at their last available follow-up assessment, were classified as MCI converters. Additionally, participants who met these neuropsychological criteria had to receive a clinical diagnosis of MCI or dementia on at least one follow-up visit to be finally classified as a diagnostic converter. Incident dementia was diagnosed by the ADNI study physicians based on established diagnostic criteria.[11]

## 2.2 DELCODE

In DELCODE; the presence of SCD was indicated by the cohort's participant groups, specifically memory clinic patients with SCD and control participants without SCD (see section 1.2). The cognitive tests from the DELCODE test battery, which were included in our analyses, are listed in eTable 1. Raw scores from the CERAD neuropsychological assessment battery, including the Trail Making Test, were converted into age-, sex/gender-, and education-adjusted z-scores based on established norms.[12] Test results from the Free and Cued Selective Reminding Test, Symbol-Digit Substitution Test, and WMS-R Digit Spans were converted into age-, sex/gender-, and education-adjusted z-scores based on unpublished regression-based norms.

For the identification of MNPD, we calculated each participant's median z-score of the 12 cognitive measures listed in eTable 1. Incident MCI was diagnosed in a two-step review process by a consensus panel of neuropsychologists. First, study participants with signs of potential cognitive decline were identified algorithmically. Then these participants were reviewed individually based on their longitudinal cognitive and clinical profiles.[13] Participants had to show signs of longitudinal cognitive decline, have one test score of z≤-1.5 and a second test score of z≤-1 in the same cognitive domain (eTable 1), and still meet this criterion at their last available assessment, to be classified as MCI converters. Incident dementia was diagnosed by the DELCODE study physicians based on established diagnostic criteria.[11,14–19] These diagnoses were additionally checked for inconsistencies by a review panel.

## 2.3 NACC

For the assessment of SCD, the dichotomous item “Does the subject report a decline in memory (relative to previously attained abilities)” was used in all UDS versions. The UDS-1 and UDS-2 neuropsychological assessment batteries included the same tests, while new tests were introduced in UDS-3. The tests from both versions, which we analyzed, are listed in eTable 1. Raw test scores were converted into age-, sex/gender-, and education-adjusted z-scores based on published norms.[8,9] UDS-1/2 norms were used for the UDS-1/2 data and UDS-3 norms for the UDS-3 data.

For the identification of MNPD, we calculated each participant’s median z-score of eight cognitive measures in the UDS-1/2 and of nine cognitive measures in the UDS-3 (eTable 1). Incident MCI was identified algorithmically. We assigned the cognitive tests to the domain of Learning and Memory, Language, Processing Speed and Attention, and Working Memory (eTable 1). At least one test with a score of z≤-1.5 and a second test with a score of z≤-1.0 in the same cognitive domain were required to fulfill the criterion for MCI. Only participants, who still met this criterion at their last available follow-up assessment, were classified as MCI converters. Additionally, participants who met these neuropsychological criteria had to receive a clinical diagnosis of MCI or dementia on at least one follow-up visit to be finally classified as a diagnostic converter. Incident dementia was diagnosed by the ADRC physicians in accordance with standard diagnostic criteria.[11,14–17,19–24]

# 3 Biomarker assessments

## 3.1 ADNI

The acquisition of positron emission tomography (PET) scans followed standardized assessment and quality control procedures. We included [18F]Florbetapir PET data from ADNI-2 as well as [18F]Florbetaben and [18F]Flortaucipir PET data from ADNI-3 in our analyses. We used the standardized uptake value ratio (SUVR) data, which is processed and provided by the ADNI PET Core at the University of California, Berkeley. Amyloid pathology was measured with the cortical summary region SUVR normalized by the whole cerebellum. Tau pathology was measured with the meta-temporal SUVR (entorhinal, amygdala, fusiform, inferior temporal, and middle temporal regions of interest) normalized by the inferior cerebellar gray matter. Based on cut-off values provided by the ADNI PET Core, amyloid pathology was defined as a Florbetapir SUVR≥1.11 or a Florbetaben SUVR≥1.08. In line with work by other research groups,[25,26] tau positivity was defined by a meta-temporal SUVR greater than or equal to 2SD above the mean of amyloid PET-negative and cognitively normal ADNI participants (M=1.17, SD=0.07; Cut-Off: 1.30).

In ADNI-1, ADNI-2, and ADNI -3, Cerebrospinal fluid (CSF) samples were collected, frozen, and shipped to the ADNI Biomarker Core according to standardized procedures. CSF concentrations (pg/ml) of amyloid-β42 (Aβ42) and phosphorylated tau ($p\text{-}tau_{181}$) were measured with fully automated Roche Elecsys® immunoassays by the ADNI Biomarker Core at the University of Pennsylvania. Amyloid pathology was defined as CSF Aβ42≤981 (pg/ml) and tau pathology was defined as CSF $p\text{-}tau_{181}$≥24.30 (pg/ml) based on previously published cut-offs.[27]

## 3.2 DELCODE

[18F]Florbetaben PET scans were conducted in a sub-sample of SCD patients and followed standardized assessment procedures. The raw imaging data were processed and Centiloid values were calculated by the DZNE PET research group. Amyloid pathology was defined by a Centiloid score ≥24.4.[28]

In both the SCD and control groups, CSF samples were collected, frozen, and shipped to the DZNE Biorepository according to standardized procedures. CSF biomarker concentrations were measured centrally in one laboratory. CSF Aβ42/40 was measured on the Mesoscale Diagnostics platform and CSF $p\text{-}tau_{181}$ was measured with the Fujirebio Innotest® assay.[4] Amyloid positivity was defined as CSF Aβ42/40 ≤0.08 and tau pathology was defined as CSF $p\text{-}tau_{181}$ ≥73.65 (pg/ml) based on previously published mixture-modelling cut-offs.[5]

## 3.3 NACC

The standardized collection of biomarker and imaging findings was introduced with the UDS-3 in 2015.[7] If a CSF, PET, or MRI assessment was conducted, ADRC clinicians are asked to indicate whether abnormalities were found, based on local standards for positivity. For the assessment of amyloid pathology, we used the dichotomous yes/no items “Abnormally elevated amyloid on PET” and “Abnormally low amyloid in CSF”. For the assessment of tau pathology, we used the dichotomous items “Tau PET evidence for AD” and “Abnormally elevated CSF tau or ptau”.

## 4 Adjustment for race and Hispanic ethnicity

In NACC, participants self-reported their race(s) during the NACC intake interview and were able to give up to three answers. These were assigned into the following categories along National Institutes of Health (NIH) guidelines: White, Black or African American, American Indian or Alaska Native, Native Hawaiian or Pacific Islander, Asian, and Multiracial. Additionally, participants were reported whether they are of Hispanic/Latino ethnicity (0 = No, 1 = Yes) regardless of race.

In ADNI, participants reported their race(s) along the following categories: White, Black or African American, American Indian or Alaska Native, Native Hawaiian or Pacific Islander, Asian, and more than one race. Additionally, they were asked to report their ethnicity (1 = Hispanic or Latino, 2 = not Hispanic or Latino).

We repeated our core analyses in the pooled ADNI and NACC sample of participants with baseline race and ethnicity data, once without adjustment for race and ethnicity and then with adjustment for these factors. All models were adjusted for the participants´ age and sex/gender, while the Cox and restricted mean survival time (RMST) regression models were additionally adjusted for years of education. Race was coded along the aforementioned categories. Participants identifying as White served as the reference group. Ethnicity was coded as 0 = Not Hispanic/Latino and 1 = Hispanic/Latino.

## 5 Adjustment for vascular risk factors and ApoE

In ADNI, DELCODE, and NACC diagnoses of hypertension, hypercholesterolemia, and diabetes were derived from the self- and informant-reported medical histories of the participants. Past or present smoking was defined by self-reported information. The participants' body mass index (BMI) was calculated from height and weight assessments conducted during in-person study visits. In ADNI and DELCODE apolipoprotein E (APOE) genotyping was conducted in the cohort's central laboratories according to standardized procedures. In NACC, APOE genotyping was conducted either locally in the ADRCs or by the Alzheimer's Disease Generic Consortium (ADGC) and National Cell Repository for Alzheimer's Disease (NCRAD).

The core analyses in the pooled ADNI, DELCODE, and NACC samples were repeated in participants with complete data in these additional covariates. First without adjustment for the additional covariates, then with adjustment for BMI, smoking (0 = never smokers, 1 = past/present smokers), hypertension, hypercholesterolemia, and diabetes (0 = absent, 1 = present), and finally with additional adjustment for APOE genotype (0 = no ε4 allele, 1 = 1/2 ε4 alleles). All models were adjusted for the participants´ age and sex/gender, while the Cox and RMST regression models were additionally adjusted for years of education.

## Figure S1. Flow diagram of the sample selection in ADNI

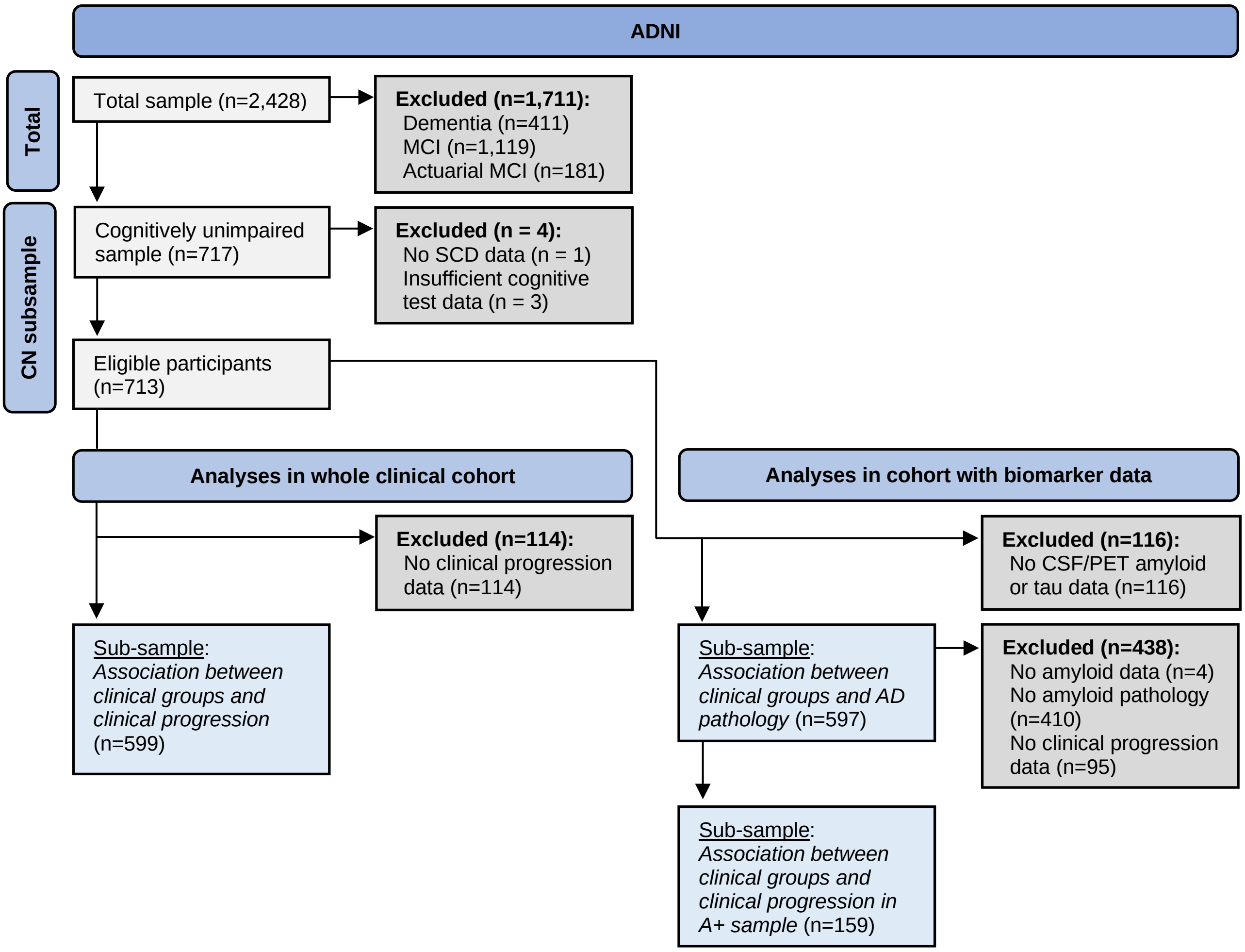


Notes. Selection and size of the analytical samples in ADNI. Participants who were classified as cognitively normal, but met the actuarial MCI criterion, we applied at follow-up (see section 2.1), at baseline were excluded from the analyses. Additionally, participants who did not have sufficient neuropsychological test data to determine the presence/absence of this actuarial definition of MCI or MNPD at baseline were excluded. Abbreviations: AD = Alzheimer´s disease; CSF = cerebrospinal fluid; MCI = mild cognitive impairment; PET = positron emission tomography; SCD = subjective cognitive decline

## Figure S2. Flow diagram of the sample selection in DELCODE

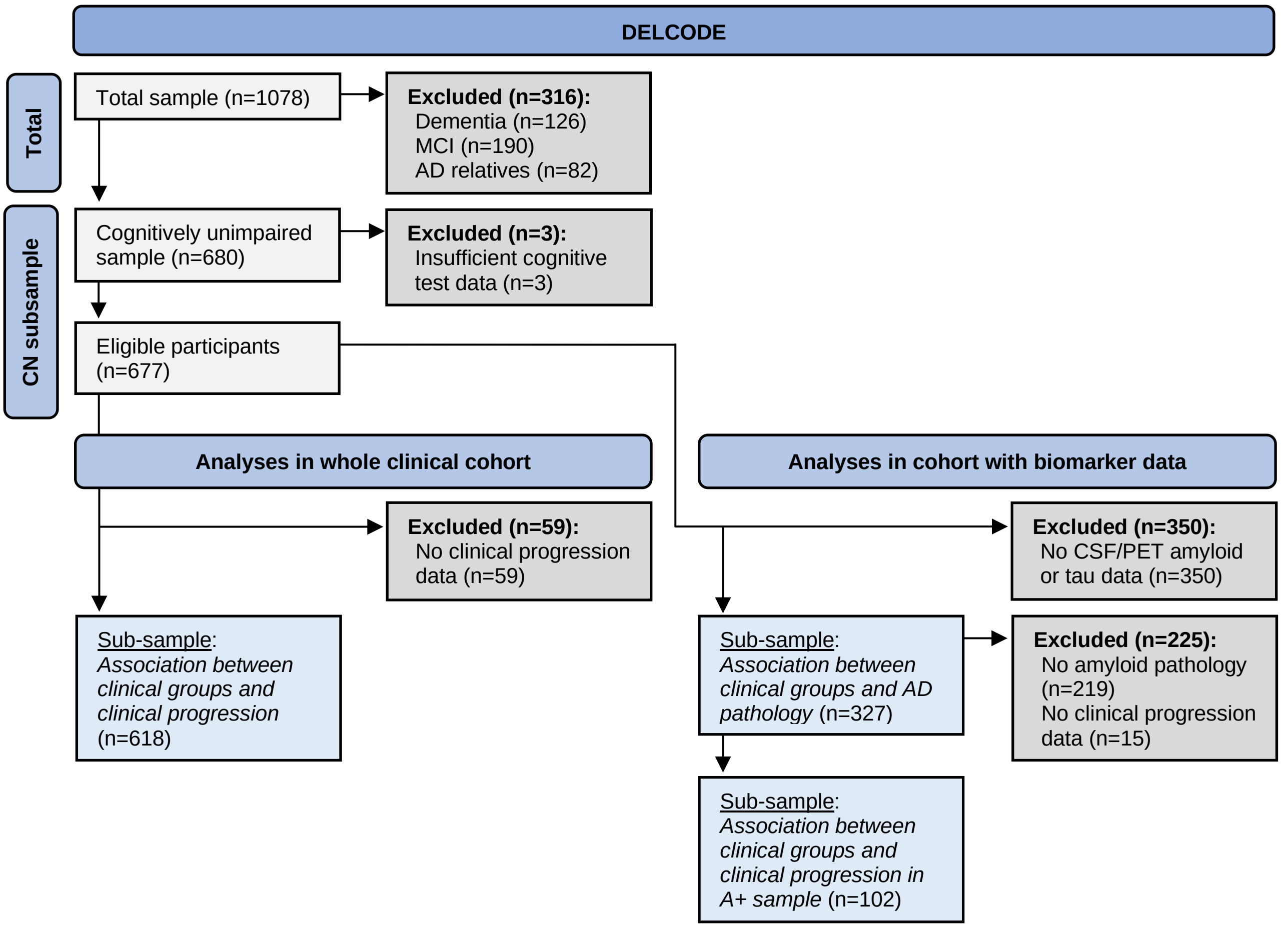


Notes. Selection and size of the analytical samples in DELCODE. Participants who did not have sufficient neuropsychological test data to determine the presence/absence of MCI or MNPD at baseline were excluded. Abbreviations: AD = Alzheimer´s disease; CSF = cerebrospinal fluid; MCI = mild cognitive impairment; PET = positron emission tomography; SCD = subjective cognitive decline

## Figure S3. Flow diagram of the sample selection in NACC

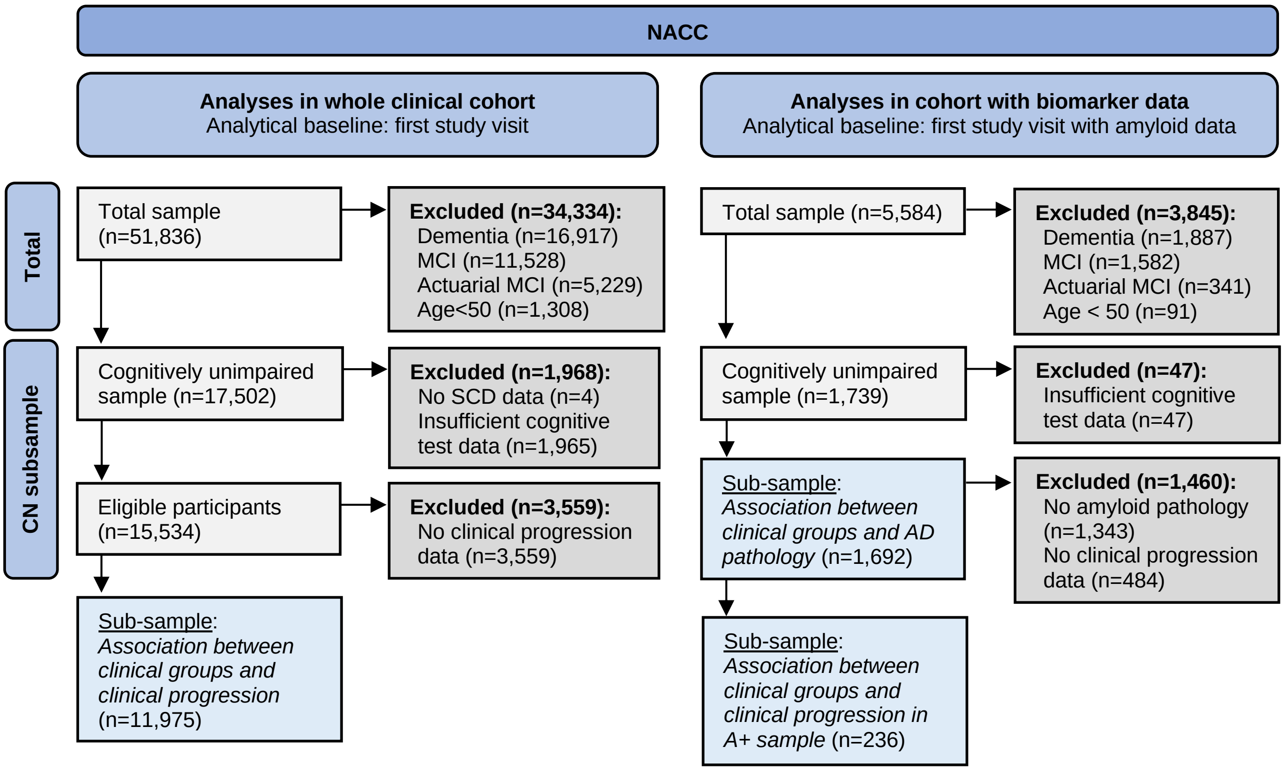


Notes. Selection and size of the analytical samples in NACC. Participants who were classified as cognitively normal, but met the actuarial MCI criterion, we applied at follow-up (see section 2.3), at baseline were excluded from the analyses. Additionally, participants who did not have sufficient neuropsychological test data to determine the presence/absence of this actuarial definition of MCI or MNPD at baseline were excluded. Abbreviations: AD = Alzheimer´s disease; CSF = cerebrospinal fluid; MCI = mild cognitive impairment; PET = positron emission tomography; SCD = subjective cognitive decline

## Figure S4. Sensitivity, specificity, positive predictive value curves over five years of follow-up (Predictive value of clinical groups)

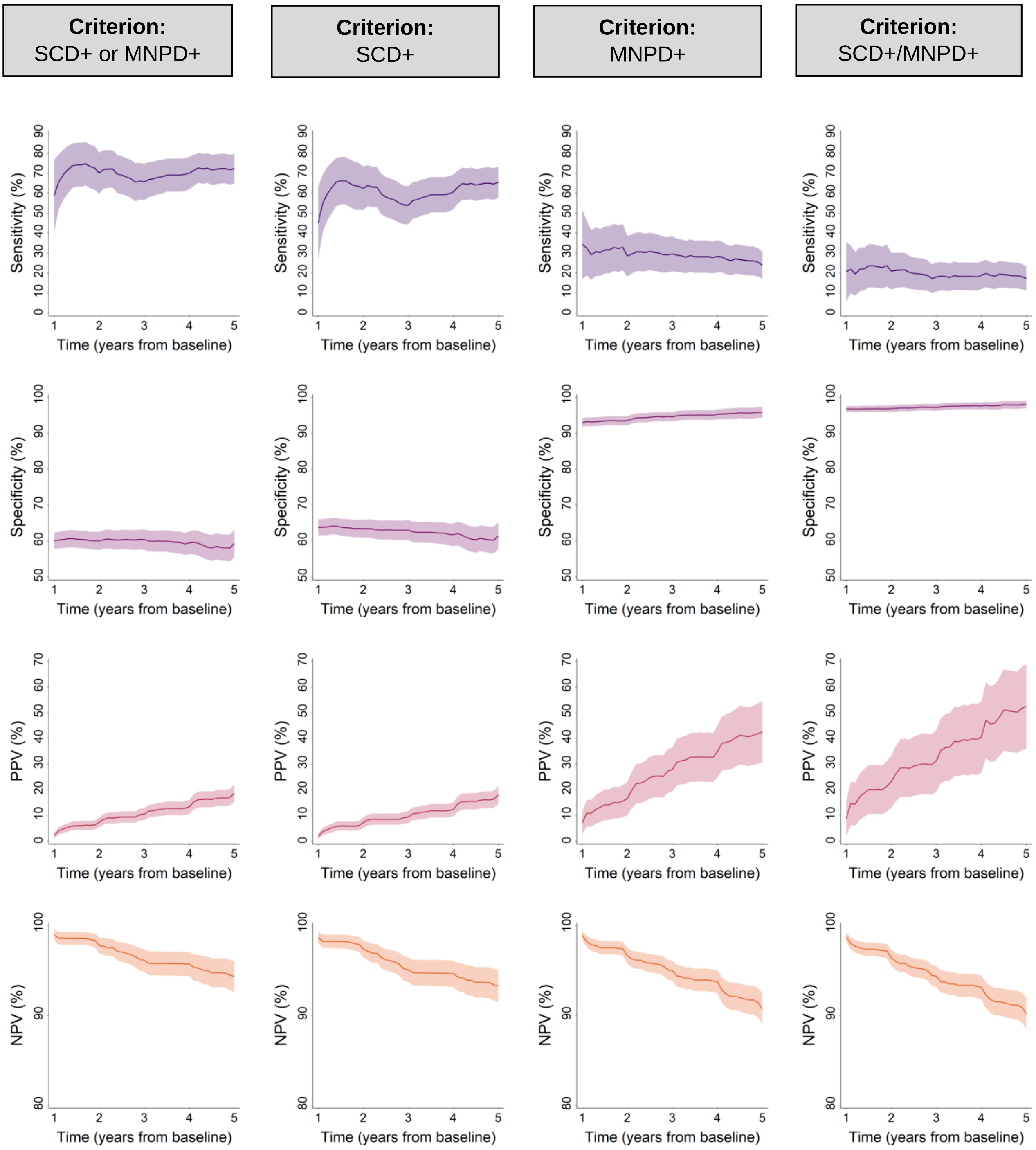


Notes. Time-dependent inverse probability of censoring weighting (IPCW) estimates of sensitivity, specificity, and positive/negative predictive values. Results estimate the prognostic value of different clinical criteria for the progression to MCI over five years of follow-up (in participants with baseline amyloid data). The following clinical criteria were compared: presence of SCD or MNPD [SCD+ or MNPD+], presence of SCD [SCD+], presence of MNPD [MNPD+], and presence of both SCD and MNPD [SCD+/MNPD+]. Abbreviations: MNPD = minor neuropsychological deficits (present +, absent -); NPV = negative predictive value; PPV = positive predictive value; SCD = subjective cognitive decline (present +, absent -).

**Figure S5. Sensitivity, specificity, positive predictive value curves over five years of follow-up (Predictive value of amyloid positivity in different clinical groups)**

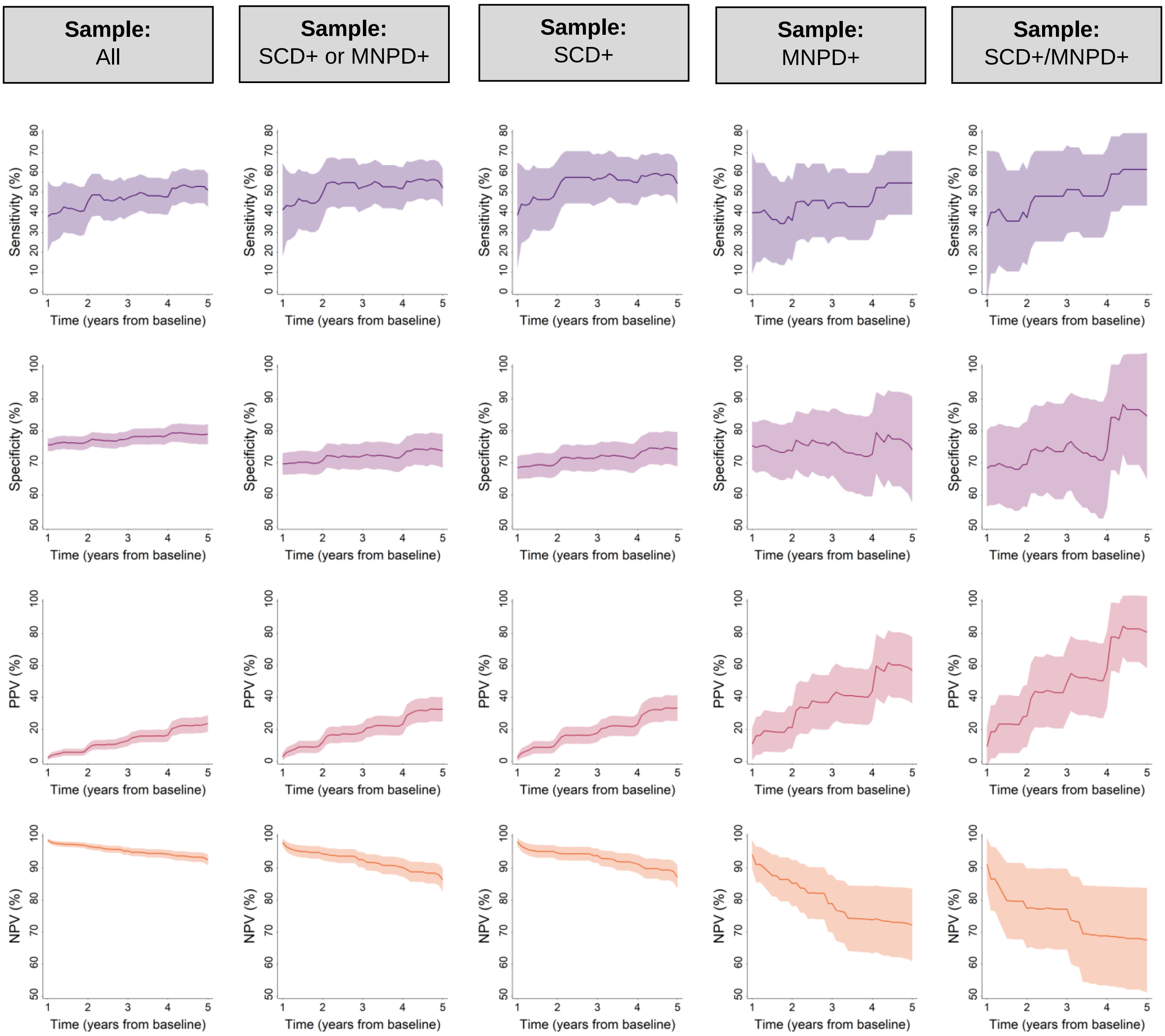


Notes. Time-dependent inverse probability of censoring weighting (IPCW) estimates of sensitivity, specificity, and positive/negative predictive values. Results estimate the prognostic value of amyloid positivity for the progression to MCI over five years of follow-up (in different clinical groups). The clinical groups were compared: all cognitively normal groups [All], participants with SCD or MNPD [SCD+ or MNPD+], participants with SCD [SCD+], participants with MNPD [MNPD+], and participants with SCD and MNPD [SCD+/MNPD+]. Abbreviations: MNPD = minor neuropsychological deficits (present +, absent -); NPV = negative predictive value; PPV = positive predictive value; SCD = subjective cognitive decline (present +, absent -).

## Table S1. Neuropsychological assessment batteries

| Cognitive Domain: Learning and Memory | | | |
|---|---|---|---|
| **ADNI** | **DELCODE** | **NACC UDS-1/2** | **NACC UDS-3** |
| WMS-R<br>Logical Memory<br>Immediate Recall<br><br>WMS-R<br>Logical Memory<br>Delayed Recall<br><br>RAVLT<br>Immediate Recall<br><br>RAVLT<br>Delayed Recall<br><br>RAVLT<br>Recognition | CERAD Word List<br>Immediate Recall<br><br>CERAD Word List<br>Delayed Recall<br><br>CERAD Word List<br>Recognition<br><br>FCSRT<br>Free Recall | WMS-R<br>Logical Memory<br>Immediate Recall<br><br>WMS-R<br>Logical Memory<br>Delayed Recall | Craft Story 21<br>Immediate Recall<br><br>Craft Story 21<br>Delayed Recall<br><br>Benson Figure<br>Delayed Recall |
| **Cognitive Domain: Language** | | | |
| **ADNI** | **DELCODE** | **NACC UDS-1/2** | **NACC UDS-3** |
| BNT (ADNI-1/2) or<br>MINT (ADNI-3)<br><br>Verbal Fluency<br>Animals | BNT (15 Items)<br><br>Verbal Fluency<br>Animals | BNT<br><br>Verbal Fluency<br>Animals | MINT<br><br>Verbal Fluency<br>Animals |
| **Cognitive Domain: Processing Speed and Attention** | | | |
| **ADNI** | **DELCODE** | **NACC UDS-1/2** | **NACC UDS-3** |
| TMT-A<br>TMT-B | TMT-A<br>TMT-B<br>SDMT | TMT-A<br>TMT-B | TMT-A<br>TMT-B |
| **Cognitive Domain: Working Memory** | | | |
| **ADNI** | **DELCODE** | **NACC UDS-1/2** | **NACC UDS-3** |
| | WMS-R<br>Digits Span Forward<br><br>WMS-R<br>Digits Span Backward<br><br>FCSRT<br>Serial Subtractions | WMS-R<br>Digits Span Forward<br><br>WMS-R<br>Digits Span Backward | Number Span<br>Forward<br><br>Number Span<br>Backward |

Abbreviations: BNT = Boston Naming Test; CERAD = Consortium to Establish a Registry for Alzheimer´s Disease – Neuropsychological Assessment Battery; FCSRT = Free and Cued Selective Reminding Test; MINT = Multilingual Naming Test; RAVLT = Rey Auditory Verbal Learning Test; SDMT = Symbol Digit Modalities Test; TMT = Trail Making Test; WMS-R = Wechsler Memory Scale – Revised

## Table S2. Baseline characteristics of participants with baseline amyloid and progression data

| Amyloid-positive participants | SCD-/MNPD- | SCD-/MNPD+ | SCD+/MNPD- | SCD+/MNPD+ |
|---|---|---|---|---|
| n | 253 | 17 | 204 | 23 |
| Age (years), M (SD) | 72.8 (6.8) | 71.9 (6.0) | 73.4 (6.4) | 75.9 (4.7) |
| Education (years), M (SD) | 16.5 (2.6) | 17.1 (2.8) | 15.9 (2.9) | 16.3 (2.9) |
| Sex (female), n (%) | 146 (57.7%) | 8 (47.1%) | 104 (51.0%) | 9 (39.1%) |
| CDR-SOB, M (SD) | 0.1 (0.3) | 0.0 (0.0) | 0.3 (0.6) | 0.6 (0.8) |
| Follow-up (years), M (SD) | 3.9 (2.7) | 3.9 (2.9) | 4.0 (2.5) | 3.9 (2.2) |
| Progression to MCI, n (%)[a] | 30 (12.3%) | 6 (37.5%) | 54 (27.7%) | 17 (73.9%) |
| Progression to dementia, n (%)[b] | 13 (5.1%) | 0 (0%) | 17 (8.4%) | 6 (26.1%) |

Notes. Years of follow-up were truncated at 10 years (maximum follow-up in regression analyses). Abbreviations: CDR-SOB = Clinical Dementia Rating – Sum of Boxes; MCI = mild cognitive impairment; MNPD = minor neuropsychological deficits (present +, absent -); SCD = subjective cognitive decline (present +, absent -).

[a] Data missing in 20 individuals (n=10 SCD-/MNPD-; n=1 SCD-/MNPD+; n=9 SCD+/MNPD-)
[b] Data missing in 1 individual (n=1 SCD+/MNPD-)

## Table S3. Baseline characteristics of participants with progression data and participants with biomarker data (ADNI)

| **Participants with progression data** | **SCD-/MNPD-** | **SCD-/MNPD+** | **SCD+/MNPD-** | **SCD+/MNPD+** |
|---|---|---|---|---|
| n | 406 | 21 | 159 | 13 |
| Age (years), M (SD) | 72.9 (6.3) | 71.8 (4.8) | 72.3 (5.9) | 71.5 (6.2) |
| Education (years), M (SD) | 16.6 (2.6) | 16.4 (2.8) | 16.1 (2.6) | 17.2 (2.7) |
| Sex (female), n (%) | 217 (53.4%) | 13 (61.9%) | 88 (55.3%) | 10 (76.9%) |
| CDR-SOB, M (SD) | 0.0 (0.1) | 0.0 (0.2) | 0.0 (0.2) | 0.1 (0.3) |
| Follow-up (years), M (SD) | 5.4 (3.1) | 5.8 (3.4) | 5.0 (2.8) | 3.8 (2.0) |
| Progression to MCI, n (%)[a] | 40 (10.1%) | 6 (30.0%) | 17 (11.0%) | 4 (33.3%) |
| Progression to dementia, n (%)[b] | 19 (4.7%) | 1 (4.8%) | 4 (2.5%) | 1 (7.7%) |
| **Participants with biomarker data** | **SCD-/MNPD-** | **SCD-/MNPD+** | **SCD+/MNPD-** | **SCD+/MNPD+** |
| n | 378 | 23 | 178 | 18 |
| Age (years), M (SD) | 71.5 (6.7) | 69.5 (4.7) | 71.9 (6.3) | 71.3 (7.6) |
| Education (years), M (SD) | 16.6 (2.4) | 16.0 (3.0) | 16.3 (2.5) | 17.2 (2.5) |
| Sex (female), n (%) | 212 (56.1%) | 12 (52.2%) | 101 (56.7%) | 12 (66.7%) |
| CDR-SOB, M (SD) | 0.0 (0.1) | 0.0 (0.1) | 0.0 (0.2) | 0.1 (0.3) |
| Amyloid positivity, n (%)[c] | 103 (27.4%) | 6 (26.1%) | 68 (38.4%) | 6 (35.3%) |
| Tau positivity, n (%)[d] | 74 (20.6%) | 3 (14.3%) | 34 (20.4%) | 6 (37.5%) |
| AT biomarker classification[e] | | | | |
| A-T-, n (%) | 220 (61.5%) | 15 (71.4%) | 91 (54.8%) | 8 (53.3%) |
| A+T-, n (%) | 64 (17.9%) | 3 (14.3%) | 41 (24.7%) | 1 (6.7%) |
| A-T+, n (%) | 37 (10.3%) | 1 (4.8%) | 11 (6.6%) | 1 (6.7%) |
| A+T+, n (%) | 37 (10.3%) | 2 (9.5%) | 23 (13.9%) | 5 (33.3%) |
| Follow-up (years), M (SD) | 4.4 (3.3) | 3.6 (3.5) | 4.2 (3.1) | 2.8 (2.5) |
| Progression to MCI, n (%)[f] | 26 (8.3%) | 5 (33.3%) | 16 (10.8%) | 4 (33.3%) |
| Progression to dementia, n (%)[g] | 12 (3.8%) | 1 (6.2%) | 4 (2.6%) | 1 (7.7%) |

Notes. Years of follow-up were truncated at 10 years (maximum follow-up in regression analyses). Abbreviations: A = amyloid positivity (present +, absent -); CDR-SOB = Clinical Dementia Rating – Sum of Boxes; MCI = mild cognitive impairment; MNPD = minor neuropsychological deficits (present +, absent -); SCD = subjective cognitive decline (present +, absent -); T = tau positivity (present +, absent -).

[a] Data missing in 15 individuals (n=8 SCD-/MNPD-; n=1 SCD-/MNPD+; n=5 SCD+/MNPD-; n=1 SCD+/MNPD+)
[b] Data missing in 2 individuals (n=2 SCD+/MNPD-)
[c] Data missing in 4 individuals (n=2 SCD-/MNPD-; n=1 SCD+/MNPD-; n=1 SCD+/MNPD+)
[d] Data missing in 33 individuals (n=18 SCD-/MNPD-; n=2 SCD-/MNPD+; n=11 SCD+/MNPD-; n=2 SCD+/MNPD+)
[e] Data missing in 37 individuals (n=20 SCD-/MNPD-; n=2 SCD-/MNPD+; n=12 SCD+/MNPD-; n=3 SCD+/MNPD+)
[f] Data missing in 108 individuals (n=64 SCD-/MNPD-; n=8 SCD-/MNPD+; n=30 SCD+/MNPD-; n=6 SCD+/MNPD+)
[g] Data missing in 97 individuals (n=58 SCD-/MNPD-; n=7 SCD-/MNPD+; n=27 SCD+/MNPD-; n=5 SCD+/MNPD+)

## Table S4. Baseline characteristics of participants with baseline amyloid and progression data (ADNI)

| Amyloid-positive participants | SCD-/MNPD- | SCD-/MNPD+ | SCD+/MNPD- | SCD+/MNPD+ |
|---|---|---|---|---|
| n | 90 | 5 | 59 | 5 |
| Age (years), M (SD) | 73.7 (6.5) | 71.6 (4.0) | 73.9 (6.6) | 77.6 (4.8) |
| Education (years), M (SD) | 16.2 (2.7) | 16.8 (3.3) | 16.3 (2.7) | 17.4 (2.1) |
| Sex (female), n (%) | 55 (61.1%) | 4 (80.0%) | 33 (55.9%) | 3 (60.0%) |
| CDR-SOB, M (SD) | 0.0 (0.1) | 0.0 (0.0) | 0.0 (0.1) | 0.3 (0.4) |
| Follow-up (years), M (SD) | 4.7 (3.0) | 6.2 (2.3) | 4.7 (2.4) | 4.6 (2.3) |
| Progression to MCI, n (%)[a] | 16 (18.4%) | 3 (60.0%) | 11 (19.0%) | 4 (80.0%) |
| Progression to dementia, n (%)[b] | 9 (10.0%) | 0 (0%) | 4 (6.9%) | 1 (20.0%) |

Notes. Years of follow-up were truncated at 10 years (maximum follow-up in regression analyses). Abbreviations: CDR-SOB = Clinical Dementia Rating – Sum of Boxes; MCI = mild cognitive impairment; MNPD = minor neuropsychological deficits (present +, absent -); SCD = subjective cognitive decline (present +, absent -).

[a] Data missing in 4 individuals (n=3 SCD-/MNPD-; n=1 SCD+/MNPD-)
[b] Data missing in 1 individual (n=1 SCD+/MNPD-)

## Table S5. Baseline characteristics of participants with progression data and participants with biomarker data (DELCODE)

| **Participants with progression data** | **SCD-/MNPD-** | **SCD-/MNPD+** | **SCD+/MNPD-** | **SCD+/MNPD+** |
|---|---|---|---|---|
| n | 203 | 9 | 362 | 44 |
| Age (years), M (SD) | 69.1 (5.4) | 67.1 (5.3) | 70.7 (6.0) | 72.0 (5.9) |
| Education (years), M (SD) | 14.7 (2.8) | 17.2 (1.4) | 14.7 (2.9) | 15.9 (2.9) |
| Sex (female), n (%) | 117 (57.6%) | 3 (33.3%) | 173 (47.8%) | 17 (38.6%) |
| CDR-SOB, M (SD) | 0.0 (0.1) | 0.0 (0.0) | 0.3 (0.5) | 0.6 (0.7) |
| Follow-up (years), M (SD) | 6.2 (2.4) | 6.9 (1.8) | 5.5 (2.1) | 4.9 (2.6) |
| Progression to MCI, n (%)[a] | 18 (8.9%) | 5 (55.6%) | 94 (26.1%) | 29 (65.9%) |
| Progression to dementia, n (%) | 0 (0%) | 0 (0%) | 13 (3.6%) | 6 (13.6%) |
| **Participants with biomarker data** | **SCD-/MNPD-** | **SCD-/MNPD+** | **SCD+/MNPD-** | **SCD+/MNPD+** |
| n | 87 | 4 | 208 | 28 |
| Age (years), M (SD) | 68.6 (5.0) | 66.8 (3.6) | 70.8 (5.9) | 72.9 (5.5) |
| Education (years), M (SD) | 14.5 (2.8) | 15.8 (2.2) | 14.9 (3.0) | 16.0 (3.0) |
| Sex (female), n (%) | 47 (54.0%) | 0 (0%) | 87 (41.8%) | 10 (35.7%) |
| CDR-SOB, M (SD) | 0.1 (0.2) | 0.0 (0.0) | 0.4 (0.6) | 0.5 (0.7) |
| Amyloid positivity, n (%) | 22 (25.3%) | 1 (25.0%) | 71 (34.1%) | 14 (50.0%) |
| Tau positivity, n (%)[b] | 6 (6.9%) | 1 (25.0%) | 25 (13.4%) | 7 (28.0%) |
| AT biomarker classification[c] | | | | |
| A-T-, n (%) | 63 (72.4%) | 3 (75.0%) | 117 (62.9%) | 12 (48.0%) |
| A+T-, n (%) | 18 (20.7%) | 0 (0%) | 44 (23.7%) | 6 (24.0%) |
| A-T+, n (%) | 2 (2.3%) | 0 (0%) | 6 (3.2%) | 1 (4.0%) |
| A+T+, n (%) | 4 (4.6%) | 1 (25.0%) | 19 (10.2%) | 6 (24.0%) |
| Follow-up (years), M (SD) | 6.0 (2.6) | 5.1 (3.9) | 5.4 (2.2) | 4.6 (2.3) |
| Progression to MCI, n (%)[d] | 7 (8.5%) | 1 (33.3%) | 54 (26.9%) | 17 (65.4%) |
| Progression to dementia, n (%)[e] | 0 (0%) | 0 (0%) | 8 (4.0%) | 5 (19.2%) |

Notes. Years of follow-up were truncated at 10 years (maximum follow-up in regression analyses). Abbreviations: A = amyloid positivity (present +, absent -); CDR-SOB = Clinical Dementia Rating – Sum of Boxes; MCI = mild cognitive impairment; MNPD = minor neuropsychological deficits (present +, absent -); SCD = subjective cognitive decline (present +, absent -); T = tau positivity (present +, absent -).

[a] Data missing in 3 individuals (n=1 SCD-/MNPD-; n=2 SCD+/MNPD-)
[b] Data missing in 25 individuals (n=22 SCD+/MNPD-; n=3 SCD+/MNPD+)
[c] Data missing in 25 individuals (n=22 SCD+/MNPD-; n=3 SCD+/MNPD+)
[d] Data missing in 15 individuals (n=5 SCD-/MNPD-; n=1 SCD-/MNPD+; n=7 SCD+/MNPD-; n=2 SCD+/MNPD+)
[e] Data missing in 15 individuals (n=5 SCD-/MNPD-; n=1 SCD-/MNPD+; n=7 SCD+/MNPD-; n=2 SCD+/MNPD+)

## Table S6. Baseline characteristics of participants with baseline amyloid and progression data (DELCODE)

| Amyloid-positive participants | SCD-/MNPD- | SCD-/MNPD+ | SCD+/MNPD- | SCD+/MNPD+ |
|---|---|---|---|---|
| n | 20 | 1 | 69 | 12 |
| Age (years), M (SD) | 70.6 (5.2) | 72.0 (/) | 72.6 (5.2) | 75.7 (5.1) |
| Education (years), M (SD) | 14.9 (3.1) | 18.0 (/) | 14.9 (3.0) | 15.6 (3.2) |
| Sex (female), n (%) | 6 (30.0%) | 0 (0%) | 28 (40.6%) | 2 (16.7%) |
| CDR-SOB, M (SD) | 0.1 (0.2) | 0.0 (/) | 0.4 (0.7) | 0.6 (0.9) |
| Follow-up (years), M (SD) | 6.4 (2.0) | 8.3 (/) | 5.2 (2.2) | 4.3 (2.0) |
| Progression to MCI, n (%) | 4 (20.0%) | 1 (100.0%) | 28 (40.6%) | 9 (75.0%) |
| Progression to dementia, n (%) | 0 (0%) | 0 (0%) | 8 (11.6%) | 4 (33.3%) |

Notes. Years of follow-up were truncated at 10 years (maximum follow-up in regression analyses). Abbreviations: CDR-SOB = Clinical Dementia Rating – Sum of Boxes; MCI = mild cognitive impairment; MNPD = minor neuropsychological deficits (present +, absent -); SCD = subjective cognitive decline (present +, absent -).

## Table S7. Baseline characteristics of participants with progression data and participants with biomarker data (NACC)

| Participants with progression data | SCD-/MNPD- | SCD-/MNPD+ | SCD+/MNPD- | SCD+/MNPD+ |
|---|---|---|---|---|
| n | 7751 | 907 | 2905 | 412 |
| Age (years), M (SD) | 71.0 (8.6) | 71.2 (9.1) | 70.9 (8.5) | 70.6 (9.1) |
| Education (years), M (SD) | 16.0 (2.5) | 16.3 (2.7) | 16.1 (2.6) | 16.0 (2.8) |
| Sex (female), n (%) | 5075 (65.5%) | 542 (59.8%) | 1830 (63.0%) | 274 (66.5%) |
| CDR-SOB, M (SD) | 0.0 (0.2) | 0.1 (0.2) | 0.4 (0.7) | 0.5 (0.7) |
| Follow-up (years), M (SD) | 5.4 (3.3) | 4.8 (3.1) | 5.0 (3.3) | 4.3 (2.9) |
| Progression to MCI, n (%)[a] | 694 (9.3%) | 190 (21.7%) | 447 (16.0%) | 122 (30.5%) |
| Progression to dementia, n (%) | 332 (4.3%) | 60 (6.6%) | 214 (7.4%) | 46 (11.2%) |
| **Participants with biomarker data** | **SCD-/MNPD-** | **SCD-/MNPD+** | **SCD+/MNPD-** | **SCD+/MNPD+** |
| n | 1143 | 98 | 400 | 51 |
| Age (years), M (SD) | 70.4 (7.3) | 69.2 (7.4) | 71.6 (8.1) | 70.3 (8.0) |
| Education (years), M (SD) | 16.5 (2.3) | 16.6 (2.4) | 16.6 (2.5) | 16.2 (2.6) |
| Sex (female), n (%) | 720 (63.0%) | 48 (49.0%) | 233 (58.2%) | 32 (62.7%) |
| CDR-SOB, M (SD) | 0.1 (0.3) | 0.2 (0.4) | 0.4 (0.7) | 0.5 (0.7) |
| Amyloid positivity, n (%) | 217 (19.0%) | 18 (18.4%) | 106 (26.5%) | 12 (23.5%) |
| Tau positivity, n (%)[b] | 76 (12.5%) | 3 (6.5%) | 34 (17.4%) | 3 (14.3%) |
| AT biomarker classification[c] | | | | |
| A-T-, n (%) | 452 (74.6%) | 39 (84.8%) | 137 (70.3%) | 17 (81.0%) |
| A+T-, n (%) | 78 (12.9%) | 4 (8.7%) | 24 (12.3%) | 1 (4.8%) |
| A-T+, n (%) | 45 (7.4%) | 1 (2.2%) | 16 (8.2%) | 1 (4.8%) |
| A+T+, n (%) | 31 (5.1%) | 2 (4.3%) | 18 (9.2%) | 2 (9.5%) |
| Follow-up (years), M (SD) | 2.5 (2.5) | 1.7 (1.9) | 2.3 (2.4) | 2.1 (2.3) |
| Progression to MCI, n (%)[d] | 37 (4.8%) | 8 (13.3%) | 28 (10.6%) | 10 (32.3%) |
| Progression to dementia, n (%)[e] | 12 (1.5%) | 0 (0%) | 9 (3.1%) | 1 (3.2%) |

Notes. Years of follow-up were truncated at 10 years (maximum follow-up in regression analyses). Abbreviations: A = amyloid positivity (present +, absent -); CDR-SOB = Clinical Dementia Rating – Sum of Boxes; MCI = mild cognitive impairment; MNPD = minor neuropsychological deficits (present +, absent -); SCD = subjective cognitive decline (present +, absent -); T = tau positivity (present +, absent -).

[a] Data missing in 404 individuals (n=249 SCD-/MNPD-; n=31 SCD-/MNPD+; n=112 SCD+/MNPD-; n=12 SCD+/MNPD+)
[b] Data missing in 824 individuals (n=537 SCD-/MNPD-; n=52 SCD-/MNPD+; n=205 SCD+/MNPD-; n=30 SCD+/MNPD+)
[c] Data missing in 824 individuals (n=537 SCD-/MNPD-; n=52 SCD-/MNPD+; n=205 SCD+/MNPD-; n=30 SCD+/MNPD+)
[d] Data missing in 568 individuals (n=375 SCD-/MNPD-; n=38 SCD-/MNPD+; n=135 SCD+/MNPD-; n=20 SCD+/MNPD+)
[e] Data missing in 481 individuals (n=319 SCD-/MNPD-; n=35 SCD-/MNPD+; n=107 SCD+/MNPD-; n=20 SCD+/MNPD+)

## Table S8. Baseline characteristics of participants with baseline amyloid and progression data (NACC)

| Amyloid-positive participants | SCD-/MNPD- | SCD-/MNPD+ | SCD+/MNPD- | SCD+/MNPD+ |
|---|---|---|---|---|
| n | 143 | 11 | 76 | 6 |
| Age (years), M (SD) | 72.5 (7.2) | 72.1 (7.2) | 73.7 (7.2) | 74.8 (4.3) |
| Education (years), M (SD) | 16.8 (2.4) | 17.2 (2.8) | 16.4 (2.8) | 17.0 (2.8) |
| Sex (female), n (%) | 85 (59.4%) | 4 (36.4%) | 43 (56.6%) | 4 (66.7%) |
| CDR-SOB, M (SD) | 0.1 (0.4) | 0.0 (0.0) | 0.4 (0.7) | 0.8 (0.8) |
| Follow-up (years), M (SD) | 3.0 (2.2) | 2.5 (2.1) | 2.5 (2.0) | 2.5 (2.4) |
| Progression to MCI, n (%)[a] | 10 (7.4%) | 2 (20.0%) | 15 (22.1%) | 4 (66.7%) |
| Progression to dementia, n (%) | 4 (2.8%) | 0 (0%) | 5 (6.6%) | 1 (16.7%) |

Notes. Years of follow-up were truncated at 10 years (maximum follow-up in regression analyses). Abbreviations: CDR-SOB = Clinical Dementia Rating – Sum of Boxes; MCI = mild cognitive impairment; MNPD = minor neuropsychological deficits (present +, absent -); SCD = subjective cognitive decline (present +, absent -).

[a] Data missing in 16 individuals (n=7 SCD-/MNPD-; n=1 SCD-/MNPD+; n=8 SCD+/MNPD-)
[b] Data missing in 71 individuals (n=49 SCD-/MNPD-; n=2 SCD-/MNPD+; n=20 SCD+/MNPD-)

## Table S9. Cox regression results for the main effects of SCD and MNPD (Pooled samples and separate cohorts)

| | Pooled (ADNI, DELCODE, NACC) | | NACC | | Pooled (ADNI, DELCODE) | | ADNI | | DELCODE | |
|---|---|---|---|---|---|---|---|---|---|---|
| **All participants** | **HR [95% CI]** | ***p*** | **HR [95% CI]** | ***p*** | **HR [95% CI]** | ***p*** | **HR [95% CI]** | ***p*** | **HR [95% CI]** | ***p*** |
| **Outcome**: MCI | | | | | | | | | | |
| SCD+ | 1.98 [1.79, 2.19] | <0.001 | 1.95 [1.75, 2.16] | <0.001 | 2.38 [1.74, 3.26] | <0.001 | 1.41 [0.83, 2.39] | 0.204 | 3.26 [2.07, 5.12] | <0.001 |
| MNPD+ | 3.16 [2.81, 3.56] | <0.001 | 2.98 [2.63, 3.38] | <0.001 | 5.41 [3.83, 7.65] | <0.001 | 5.03 [2.52, 10.04] | <0.001 | 5.32 [3.55, 7.97] | <0.001 |
| **Outcome**: Dementia | | | | | | | | | | |
| SCD+ | 2.12 [1.82, 2.47] | <0.001 | 2.12 [1.81, 2.48] | <0.001 | 2.48 [1.28, 4.82] | 0.007 | 0.93 [0.34, 2.53] | 0.881 | 23.43 [1.3, 424.1] | 0.033 |
| MNPD+ | 1.97 [1.61, 2.41] | <0.001 | 1.89 [1.54, 2.33] | <0.001 | 3.24 [1.46, 7.19] | 0.004 | 1.65 [0.38, 7.17] | 0.501 | 3.95 [1.35, 11.60] | 0.012 |

Notes. Main effects of SCD and MNPD (separate models) for the progression the MCI and dementia. Reference group: SCD- for models analyzing the effect of SCD and MNPD- for models analyzing the effect of MNPD. All models were adjusted for the effects of baseline age, years of education, sex/gender, and study cohort (in analyses with pooled samples). A Cox regression model with Firth’s penalized maximum likelihood estimation was used to estimate the effect of SCD on the progression to dementia in DELCODE. Abbreviations: MCI = mild cognitive impairment; MNPD = minor neuropsychological deficits (present +, absent -); SCD = subjective cognitive decline (present +, absent -).

## Table S10. Cox regression results (Pooled samples and separate cohorts)

| | Pooled (ADNI, DELCODE, NACC) | | NACC | | Pooled (ADNI, DELCODE) | | ADNI | | DELCODE | |
|---|---|---|---|---|---|---|---|---|---|---|
| **All participants** | **HR [95% CI]** | ***p*** | **HR [95% CI]** | ***p*** | **HR [95% CI]** | ***p*** | **HR [95% CI]** | ***p*** | **HR [95% CI]** | ***p*** |
| **Outcome**: MCI | | | | | | | | | | |
| SCD-/MNPD+ | 3.13 [2.68, 3.66] | <0.001 | 3.02 [2.57, 3.54] | <0.001 | 6.10 [3.16, 11.78] | <0.001 | 4.48 [1.87, 10.76] | 0.001 | 9.79 [3.55, 26.96] | <0.001 |
| SCD+/MNPD- | 1.97 [1.76, 2.20] | <0.001 | 1.96 [1.74, 2.21] | <0.001 | 2.30 [1.64, 3.24] | <0.001 | 1.34 [0.75, 2.39] | 0.329 | 3.41 [2.05, 5.68] | <0.001 |
| SCD+/MNPD+ | 6.23 [5.23, 7.42] | <0.001 | 5.74 [4.73, 6.97] | <0.001 | 10.49 [6.61, 16.65] | <0.001 | 8.22 [2.84, 23.77] | <0.001 | 14.39 [7.84, 26.42] | <0.001 |
| **Outcome**: Dementia | | | | | | | | | | |
| SCD-/MNPD+ | 1.76 [1.34, 2.31] | <0.001 | 1.78 [1.35, 2.34] | <0.001 | 1.05 [0.14, 8.00] | 0.961 | 0.92 [0.12, 7.01] | 0.937 | 16.47 [0.2, 1114.8] | 0.192 |
| SCD+/MNPD- | 2.04 [1.72, 2.41] | <0.001 | 2.07 [1.74, 2.45] | <0.001 | 2.00 [0.98, 4.09] | 0.058 | 0.76 [0.25, 2.29] | 0.622 | 18.21 [0.9, 357.1] | 0.056 |
| SCD+/MNPD+ | 4.57 [3.41, 6.10] | <0.001 | 4.25 [3.12, 5.79] | <0.001 | 8.27 [3.12, 21.92] | <0.001 | 5.12 [0.65, 39.99] | 0.120 | 60.08 [2.8, 1293.3] | 0.009 |
| **Amyloid-positive participants** | **HR [95% CI]** | ***p*** | **HR [95% CI]** | ***p*** | **HR [95% CI]** | ***p*** | **HR [95% CI]** | ***p*** | **HR [95% CI]** | ***p*** |
| **Outcome**: MCI | | | | | | | | | | |
| SCD-/MNPD+ | 4.99 [2.03, 12.26] | <0.001 | 4.14 [0.87, 19.81] | 0.075 | 5.24 [1.72, 16.03] | 0.004 | 5.38 [1.42, 20.44] | 0.014 | 7.33 [0.78, 69.11] | 0.082 |
| SCD+/MNPD- | 2.36 [1.47, 3.80] | <0.001 | 3.64 [1.56, 8.50] | 0.003 | 1.68 [0.95, 2.98] | 0.074 | 1.15 [0.53, 2.50] | 0.722 | 2.53 [0.88, 7.26] | 0.086 |
| SCD+/MNPD+ | 11.70 [5.94, 23.04] | <0.001 | 39.39 [10.84, 143.13] | <0.001 | 8.17 [3.74, 17.86] | <0.001 | 16.47 [4.70, 57.67] | <0.001 | 8.32 [2.45, 28.22] | 0.001 |
| **Outcome**: Dementia | | | | | | | | | | |
| SCD-/MNPD+ | 0.74 [0.04, 14.00] | 0.838 | 3.97 [0.14, 113.20] | 0.420 | 0.68 [0.03, 14.07] | 0.801 | 0.61 [0.03, 14.92] | 0.764 | 14.45 [0.2, 1203.9] | 0.237 |
| SCD+/MNPD- | 2.10 [0.97, 4.57] | 0.060 | 3.90 [0.82, 18.61] | 0.087 | 1.46 [0.57, 3.73] | 0.429 | 0.97 [0.29, 3.26] | 0.961 | 6.21 [0.29, 132.89] | 0.243 |
| SCD+/MNPD+ | 9.07 [2.91, 28.29] | <0.001 | 43.42 [3.42, 551.49] | 0.004 | 6.61 [1.82, 24.03] | 0.004 | 6.21 [0.72, 62.77] | 0.097 | 18.24 [0.7, 458.4] | 0.078 |

Notes. Reference group: SCD-/MNPD-. All models were adjusted for the effects of baseline age, years of education, sex/gender, and study cohort (in analyses with pooled samples). Cox regression models with Firth's penalized maximum likelihood estimation were used for the dementia progression analyses in DELCODE as well as in amyloid-positive participants from all cohorts. Abbreviations: MCI = mild cognitive impairment; MNPD = minor neuropsychological deficits (present +, absent -); SCD = subjective cognitive decline (present +, absent -).

## Table S11. Pseudo-value regression results (Pooled samples and separate cohorts)

| | Pooled (ADNI, DELCODE, NACC) | | NACC | | Pooled (ADNI, DELCODE) | | ADNI | | DELCODE | |
|---|---|---|---|---|---|---|---|---|---|---|
| **All participants** | **β [95% CI]** | ***p*** | **β [95% CI]** | ***p*** | **β [95% CI]** | ***p*** | **β [95% CI]** | ***p*** | **β [95% CI]** | ***p*** |
| **Outcome**: MCI | | | | | | | | | | |
| SCD-/MNPD+ | -1.39 [-1.64, -1.14] | <0.001 | -1.33 [-1.59, -1.08] | <0.001 | -2.55 [-4.09, -1.00] | 0.001 | -1.96 [-3.94, 0.01] | 0.052 | -3.29 [-5.44, -1.14] | 0.003 |
| SCD+/MNPD- | -0.70 [-0.82, -0.58] | <0.001 | -0.69 [-0.82, -0.55] | <0.001 | -0.91 [-1.25, -0.56] | <0.001 | -0.32 [-0.87, 0.23] | 0.255 | -1.13 [-1.64, -0.61] | <0.001 |
| SCD+/MNPD+ | -2.55 [-2.94, -2.17] | <0.001 | -2.30 [-2.70, -1.89] | <0.001 | -4.20 [-5.26, -3.15] | <0.001 | -2.58 [-4.93, -0.22] | 0.032 | -4.41 [-5.58, -3.24] | <0.001 |
| **Outcome**: Dementia | | | | | | | | | | |
| SCD-/MNPD+ | -0.24 [-0.37, -0.11] | <0.001 | -0.25 [-0.38, -0.12] | <0.001 | 0.05 [-0.48, 0.58] | 0.856 | 0.25 [-0.18, 0.67] | 0.262 | 0.11 [-0.09, 0.31] | 0.296 |
| SCD+/MNPD- | -0.30 [-0.38, -0.22] | <0.001 | -0.31 [-0.40, -0.23] | <0.001 | -0.18 [-0.34, -0.01] | 0.036 | 0.05 [-0.25, 0.35] | 0.737 | -0.35 [-0.51, -0.19] | <0.001 |
| SCD+/MNPD+ | -0.75 [-0.99, -0.51] | <0.001 | -0.71 [-0.97, -0.46] | <0.001 | -0.99 [-1.71, -0.27] | 0.007 | -0.36 [-1.30, 0.58] | 0.456 | -1.07 [-1.83, -0.32] | 0.005 |
| **Amyloid-positive participants** | **β [95% CI]** | ***p*** | **β [95% CI]** | ***p*** | **β [95% CI]** | ***p*** | **β [95% CI]** | ***p*** | **β [95% CI]** | ***p*** |
| **Outcome**: MCI | | | | | | | | | | |
| SCD-/MNPD+ | -2.60 [-5.05, -0.15] | 0.037 | -1.52 [-5.08, 2.04] | 0.403 | -3.79 [-7.46, -0.12] | 0.043 | -3.63 [-8.34, 1.07] | 0.130 | -4.51 [-6.20, -2.82] | <0.001 |
| SCD+/MNPD- | -1.29 [-2.15, -0.43] | 0.003 | -1.84 [-4.10, 0.41] | 0.110 | -0.89 [-1.86, 0.08] | 0.072 | -0.37 [-1.67, 0.93] | 0.573 | -1.13 [-3.10, 0.85] | 0.264 |
| SCD+/MNPD+ | -5.89 [-7.63, -4.15] | <0.001 | -6.88 [-10.29, -3.47] | <0.001 | -4.79 [-6.50, -3.09] | <0.001 | -6.00 [-8.88, -3.12] | <0.001 | -4.09 [-6.43, -1.74] | 0.001 |
| **Outcome**: Dementia | | | | | | | | | | |
| SCD-/MNPD+ | 0.85 [-0.16, 1.85] | 0.098 | 0.46 [-1.48, 2.40] | 0.644 | 1.35 [0.03, 2.67] | 0.045 | 1.27 [-0.02, 2.55] | 0.054 | 0.67 [0.02, 1.32] | 0.043 |
| SCD+/MNPD- | -0.49 [-1.12, 0.14] | 0.134 | -0.61 [-2.59, 1.36] | 0.542 | -0.41 [-1.07, 0.26] | 0.231 | -0.15 [-1.09, 0.79] | 0.760 | -1.05 [-1.70, -0.40] | 0.002 |
| SCD+/MNPD+ | -3.51 [-5.98, -1.04] | 0.005 | -2.38 [-6.21, 1.46] | 0.225 | -2.74 [-4.82, -0.66] | 0.010 | -1.31 [-3.83, 1.22] | 0.310 | -2.97 [-5.01, -0.93] | 0.004 |

Notes. Reference group: SCD-/MNPD-. All models were adjusted for the effects of baseline age, years of education, sex/gender, and study cohort (in analyses with pooled samples). Abbreviations: MCI = mild cognitive impairment; MNPD = minor neuropsychological deficits (present +, absent -); SCD = subjective cognitive decline (present +, absent -).

## Table S12. Baseline characteristics of participants with progression data (Participants with data on race and ethnicity)

| Participants with progression data | SCD-/MNPD- | SCD-/MNPD+ | SCD+/MNPD- | SCD+/MNPD+ |
|---|---|---|---|---|
| n | 8097 | 918 | 3039 | 423 |
| Age (years), M (SD) | 71.1 (8.5) | 71.2 (9.0) | 71.0 (8.4) | 70.6 (9.0) |
| Education (years), M (SD) | 16.0 (2.5) | 16.4 (2.7) | 16.1 (2.6) | 16.0 (2.8) |
| Sex (female), n (%) | 5255 (64.9%) | 547 (59.6%) | 1903 (62.6%) | 282 (66.7%) |
| CDR-SOB, M (SD) | 0.0 (0.2) | 0.1 (0.2) | 0.3 (0.6) | 0.4 (0.7) |
| Race | | | | |
| White | 7000 (86.5%) | 587 (63.9%) | 2644 (87.0%) | 286 (67.6%) |
| Black / African American | 754 (9.3%) | 263 (28.6%) | 248 (8.2%) | 101 (23.9%) |
| American Indian / Alaska Native | 19 (0.2%) | 8 (0.9%) | 9 (0.3%) | 5 (1.2%) |
| Native Hawaiian / Pacific Islander | 5 (0.1%) | 1 (0.1%) | 1 (0.0%) | 1 (0.2%) |
| Asian | 126 (1.6%) | 27 (2.9%) | 59 (1.9%) | 8 (1.9%) |
| Multiracial | 193 (2.4%) | 32 (3.5%) | 78 (2.6%) | 22 (5.2%) |
| Ethnicity (Hispanic), n (%) | 274 (3.4%) | 64 (7.0%) | 144 (4.7%) | 41 (9.7%) |
| Follow-up (years), M (SD) | 5.4 (3.3) | 4.8 (3.1) | 5.0 (3.2) | 4.3 (2.8) |
| Progression to MCI, n (%)[a] | 727 (9.3%) | 196 (22.1%) | 461 (15.8%) | 126 (30.7%) |
| Progression to dementia, n (%)[b] | 348 (4.3%) | 61 (6.6%) | 217 (7.1%) | 47 (11.1%) |

Notes. Years of follow-up were truncated at 10 years (maximum follow-up in regression analyses). Abbreviations: CDR-SOB = Clinical Dementia Rating – Sum of Boxes; MCI = mild cognitive impairment; MNPD = minor neuropsychological deficits (present +, absent -); SCD = subjective cognitive decline (present +, absent -).

[a] Data missing in 414 individuals (n=252 SCD-/MNPD-; n=32 SCD-/MNPD+; n=117 SCD+/MNPD-; n=13 SCD+/MNPD+)
[b] Data missing in 2 individuals (n=2 SCD+/MNPD-)

## Table S13. Cox regression results (without / with adjustment for race and ethnicity)

| | Model 1 (Pooled ADNI, NACC) | | Model 2 (Pooled ADNI, NACC) | |
|---|---|---|---|---|
| **All participants** | **HR [95% CI]** | ***p*** | **HR [95% CI]** | ***p*** |
| **Outcome**: MCI | | | | |
| SCD-/MNPD+ | 3.08 [2.63, 3.61] | <0.001 | 3.05 [2.59, 3.58] | <0.001 |
| SCD+/MNPD- | 1.93 [1.72, 2.17] | <0.001 | 1.92 [1.71, 2.16] | <0.001 |
| SCD+/MNPD+ | 5.79 [4.79, 7.01] | <0.001 | 5.73 [4.72, 6.96] | <0.001 |
| **Outcome**: Dementia | | | | |
| SCD-/MNPD+ | 1.77 [1.35, 2.32] | <0.001 | 1.98 [1.50, 2.61] | <0.001 |
| SCD+/MNPD- | 2.02 [1.70, 2.39] | <0.001 | 2.04 [1.72, 2.42] | <0.001 |
| SCD+/MNPD+ | 4.23 [3.12, 5.74] | <0.001 | 4.84 [3.55, 6.59] | <0.001 |
| **Amyloid-positive participants** | **HR [95% CI]** | ***p*** | **HR [95% CI]** | ***p*** |
| **Outcome**: MCI | | | | |
| SCD-/MNPD+ | 4.76 [1.78, 12.75] | 0.002 | 3.95 [1.45, 10.80] | 0.007 |
| SCD+/MNPD- | 2.05 [1.18, 3.57] | 0.011 | 2.02 [1.16, 3.54] | 0.013 |
| SCD+/MNPD+ | 19.74 [8.31, 46.92] | <0.001 | 17.77 [7.45, 42.40] | <0.001 |
| **Outcome**: Dementia | | | | |
| SCD-/MNPD+ | 0.77 [0.01, 6.20] | 0.853 | 0.73 [0.01, 5.80] | 0.819 |
| SCD+/MNPD- | 1.72 [0.72, 3.97] | 0.218 | 1.68 [0.69, 3.89] | 0.242 |
| SCD+/MNPD+ | 9.22 [1.64, 37.20] | 0.016 | 8.64 [1.53, 34.90] | 0.019 |

Notes. Reference group: SCD-/MNPD-. Model 1: adjustment for the effects of baseline age, years of education, sex/gender, and study cohort. Model 2: additional adjustment of the effects of race and Hispanic ethnicity. Race was coded as 0 = White (reference group), 1 = Black or African American, 3 = American Indian or Alaska Native, 4 = Native Hawaiian or Pacific Islander, 5 = Asian, 6 = Multiracial. Hispanic ethnicity was coded as 0 = No (reference group), 1 = Yes. Cox regression models with Firth's penalized maximum likelihood estimation were used for the dementia progression analyses in amyloid-positive participants. Abbreviations: MCI = mild cognitive impairment; MNPD = minor neuropsychological deficits (present +, absent -); SCD = subjective cognitive decline (present +, absent -).

## Table S14. Pseudo-value regression results (without / with adjustment for race and ethnicity)

| | Model 1 (Pooled ADNI, NACC) | | Model 2 (Pooled ADNI, NACC) | |
|---|---|---|---|---|
| **All participants** | **β [95% CI]** | ***p*** | **β [95% CI]** | ***p*** |
| **Outcome**: MCI | | | | |
| SCD-/MNPD+ | -1.37 [-1.63, -1.12] | <0.001 | -1.36 [-1.62, -1.11] | <0.001 |
| SCD+/MNPD- | -0.67 [-0.80, -0.54] | <0.001 | -0.66 [-0.80, -0.53] | <0.001 |
| SCD+/MNPD+ | -2.32 [-2.72, -1.92] | <0.001 | -2.30 [-2.70, -1.90] | <0.001 |
| **Outcome**: Dementia | | | | |
| SCD-/MNPD+ | -0.24 [-0.38, -0.11] | <0.001 | -0.30 [-0.43, -0.16] | <0.001 |
| SCD+/MNPD- | -0.30 [-0.39, -0.22] | <0.001 | -0.30 [-0.39, -0.22] | <0.001 |
| SCD+/MNPD+ | -0.71 [-0.96, -0.46] | <0.001 | -0.75 [-1.00, -0.50] | <0.001 |
| **Amyloid-positive participants** | **β [95% CI]** | ***p*** | **β [95% CI]** | ***p*** |
| **Outcome**: MCI | | | | |
| SCD-/MNPD+ | -2.30 [-5.13, 0.52] | 0.110 | -2.08 [-4.85, 0.69] | 0.141 |
| SCD+/MNPD- | -1.13 [-2.19, -0.07] | 0.037 | -1.12 [-2.18, -0.07] | 0.037 |
| SCD+/MNPD+ | -6.75 [-9.26, -4.23] | <0.001 | -6.72 [-9.08, -4.36] | <0.001 |
| **Outcome**: Dementia | | | | |
| SCD-/MNPD+ | 0.87 [-0.21, 1.94] | 0.116 | 0.88 [-0.21, 1.97] | 0.113 |
| SCD+/MNPD- | -0.34 [-1.16, 0.49] | 0.422 | -0.34 [-1.17, 0.48] | 0.412 |
| SCD+/MNPD+ | -2.52 [-5.83, 0.79] | 0.136 | -2.76 [-6.07, 0.56] | 0.103 |

Notes. Reference group: SCD-/MNPD-. Model 1: adjustment for the effects of baseline age, years of education, sex/gender, and study cohort. Model 2: additional adjustment of the effects of race and Hispanic ethnicity. Race was coded as 0 = White (reference group), 1 = Black or African American, 3 = American Indian or Alaska Native, 4 = Native Hawaiian or Pacific Islander, 5 = Asian, 6 = Multiracial. Hispanic ethnicity was coded as 0 = No (reference group), 1 = Yes. Abbreviations: MCI = mild cognitive impairment; MNPD = minor neuropsychological deficits (present +, absent -); SCD = subjective cognitive decline (present +, absent -).

## Table S15. Baseline characteristics of participants with progression data (Participants with data on vascular risk factors and APOE)

| Participants with progression data | SCD-/MNPD- | SCD-/MNPD+ | SCD+/MNPD- | SCD+/MNPD+ |
|---|---|---|---|---|
| n | 7350 | 789 | 2941 | 385 |
| Age (years), M (SD) | 71.0 (8.4) | 71.5 (8.9) | 70.9 (8.2) | 70.4 (8.7) |
| Education (years), M (SD) | 16.0 (2.6) | 16.4 (2.7) | 15.9 (2.7) | 16.1 (2.8) |
| Sex (female), n (%) | 4721 (64.2%) | 463 (58.7%) | 1790 (60.9%) | 248 (64.4%) |
| CDR-SOB, M (SD) | 0.0 (0.2) | 0.1 (0.2) | 0.3 (0.6) | 0.5 (0.7) |
| APOE-ε4+, n (%) | 2142 (29.1%) | 264 (33.5%) | 959 (32.6%) | 135 (35.1%) |
| BMI, M (SD) | 27.3 (5.2) | 28.0 (5.7) | 27.0 (5.1) | 27.7 (5.6) |
| Past / Present smoking, n (%) | 3125 (42.5%) | 300 (38.0%) | 1345 (45.7%) | 178 (46.2%) |
| Hypertension, n (%) | 3294 (44.8%) | 459 (58.2%) | 1327 (45.1%) | 215 (55.8%) |
| Hypercholesterolemia, n (%) | 3472 (47.2%) | 407 (51.6%) | 1476 (50.2%) | 194 (50.4%) |
| Diabetes, (%) | 686 (9.3%) | 117 (14.8%) | 303 (10.3%) | 62 (16.1%) |
| Follow-up (years), M (SD) | 5.7 (3.3) | 5.2 (3.1) | 5.4 (3.1) | 4.6 (2.9) |
| Progression to MCI, n (%)[a] | 681 (9.5%) | 181 (23.6%) | 503 (17.6%) | 136 (36.1%) |
| Progression to dementia, n (%)[b] | 324 (4.4%) | 52 (6.6%) | 214 (7.3%) | 51 (13.2%) |

Notes. Years of follow-up were truncated at 10 years (maximum follow-up in regression analyses). Abbreviations: APOE = Apolipoprotein E; BMI = Body Mass Index; CDR-SOB = Clinical Dementia Rating – Sum of Boxes; MCI = mild cognitive impairment; MNPD = minor neuropsychological deficits (present +, absent -); SCD = subjective cognitive decline (present +, absent -).

[a] Data missing in 271 individuals (n=164 SCD-/MNPD-; n=22 SCD-/MNPD+; n=77 SCD+/MNPD-; n=8 SCD+/MNPD+)
[b] Data missing in 2 individuals (n=2 SCD+/MNPD-)

## Table S16. Cox regression results (without / with adjustment for vascular risk factors and APOE)

| | Model 1 (ADNI, DELCODE, NACC) | | Model 2 (ADNI, DELCODE, NACC) | | Model 3 (ADNI, DELCODE, NACC) | |
|---|---|---|---|---|---|---|
| **All participants** | **HR [95% CI]** | ***p*** | **HR [95% CI]** | ***p*** | **HR [95% CI]** | ***p*** |
| **Outcome**: MCI | | | | | | |
| SCD-/MNPD+ | 3.18 [2.70, 3.75] | <0.001 | 3.17 [2.68, 3.73] | <0.001 | 3.10 [2.63, 3.66] | <0.001 |
| SCD+/MNPD- | 1.99 [1.77, 2.24] | <0.001 | 1.97 [1.75, 2.22] | <0.001 | 1.93 [1.71, 2.17] | <0.001 |
| SCD+/MNPD+ | 6.55 [5.43, 7.89] | <0.001 | 6.49 [5.38, 7.84] | <0.001 | 6.34 [5.25, 7.65] | <0.001 |
| **Outcome**: Dementia | | | | | | |
| SCD-/MNPD+ | 1.63 [1.21, 2.18] | 0.001 | 1.65 [1.23, 2.22] | 0.001 | 1.56 [1.16, 2.09] | 0.003 |
| SCD+/MNPD- | 2.07 [1.74, 2.47] | <0.001 | 2.06 [1.73, 2.45] | <0.001 | 1.98 [1.66, 2.36] | <0.001 |
| SCD+/MNPD+ | 5.14 [3.82, 6.92] | <0.001 | 5.28 [3.92, 7.11] | <0.001 | 4.74 [3.52, 6.38] | <0.001 |
| **Amyloid-positive participants** | **HR [95% CI]** | ***p*** | **HR [95% CI]** | ***p*** | **HR [95% CI]** | ***p*** |
| **Outcome**: MCI | | | | | | |
| SCD-/MNPD+ | 5.08 [2.05, 12.58] | <0.001 | 5.99 [2.40, 14.97] | <0.001 | 6.02 [2.40, 15.08] | <0.001 |
| SCD+/MNPD- | 2.26 [1.39, 3.67] | 0.001 | 2.35 [1.44, 3.83] | 0.001 | 2.39 [1.46, 3.91] | 0.001 |
| SCD+/MNPD+ | 10.41 [5.02, 21.57] | <0.001 | 9.93 [4.72, 20.88] | <0.001 | 10.07 [4.77, 21.26] | <0.001 |
| **Outcome**: Dementia | | | | | | |
| SCD-/MNPD+ | 0.67 [0.03, 12.90] | 0.790 | 0.73 [0.04, 13.86] | 0.833 | 0.68 [0.04, 12.97] | 0.798 |
| SCD+/MNPD- | 1.94 [0.87, 4.32] | 0.105 | 2.27 [0.97, 5.28] | 0.057 | 2.32 [1.00, 5.39] | 0.051 |
| SCD+/MNPD+ | 9.14 [2.89, 28.89] | <0.001 | 9.41 [2.83, 31.26] | <0.001 | 9.96 [2.97, 33.41] | <0.001 |

Notes. Reference group: SCD-/MNPD-. Model 1: adjustment for the effects of baseline age, years of education, sex/gender, and study cohort. Model 2: additional adjustment of the effects of BMI, past/present smoking, hypertension, hypercholesterolemia, and diabetes. All vascular factors were coded 0 = absent, 1 = present. Model 3: additional adjustment for APOE-ε4 positivity (coded 0 = no ε4 alleles, 1 = 1/2 ε4 alleles). Cox regression models with Firth's penalized maximum likelihood estimation were used for the dementia progression analyses in amyloid-positive participants. Abbreviations: APOE = Apolipoprotein E; MCI = mild cognitive impairment; MNPD = minor neuropsychological deficits (present +, absent -); SCD = subjective cognitive decline (present +, absent -).

## Table S17. Pseudo-value regression results (without / with adjustment for vascular risk factors and APOE)

| | Model 1 (ADNI, DELCODE, NACC) | | Model 2 (ADNI, DELCODE, NACC) | | Model 3 (ADNI, DELCODE, NACC) | |
|---|---|---|---|---|---|---|
| **All participants** | **β [95% CI]** | ***p*** | **β [95% CI]** | ***p*** | **β [95% CI]** | ***p*** |
| **Outcome**: MCI | | | | | | |
| SCD-/MNPD+ | -1.40 [-1.66, -1.13] | <0.001 | -1.40 [-1.66, -1.13] | <0.001 | -1.37 [-1.64, -1.10] | <0.001 |
| SCD+/MNPD- | -0.71 [-0.84, -0.58] | <0.001 | -0.70 [-0.83, -0.57] | <0.001 | -0.69 [-0.82, -0.56] | <0.001 |
| SCD+/MNPD+ | -2.65 [-3.06, -2.24] | <0.001 | -2.65 [-3.06, -2.24] | <0.001 | -2.61 [-3.02, -2.21] | <0.001 |
| **Outcome**: Dementia | | | | | | |
| SCD-/MNPD+ | -0.19 [-0.32, -0.06] | 0.005 | -0.20 [-0.33, -0.07] | 0.003 | -0.18 [-0.31, -0.05] | 0.007 |
| SCD+/MNPD- | -0.31 [-0.39, -0.22] | <0.001 | -0.31 [-0.39, -0.22] | <0.001 | -0.29 [-0.38, -0.21] | <0.001 |
| SCD+/MNPD+ | -0.86 [-1.12, -0.59] | <0.001 | -0.87 [-1.13, -0.60] | <0.001 | -0.85 [-1.11, -0.58] | <0.001 |
| **Amyloid-positive participants** | **β [95% CI]** | ***p*** | **β [95% CI]** | ***p*** | **β [95% CI]** | ***p*** |
| **Outcome**: MCI | | | | | | |
| SCD-/MNPD+ | -2.70 [-5.20, -0.20] | 0.034 | -2.72 [-5.15, -0.30] | 0.028 | -2.71 [-5.17, -0.25] | 0.031 |
| SCD+/MNPD- | -1.23 [-2.10, -0.36] | 0.005 | -1.19 [-2.05, -0.33] | 0.007 | -1.23 [-2.10, -0.36] | 0.005 |
| SCD+/MNPD+ | -5.79 [-7.66, -3.92] | <0.001 | -5.62 [-7.50, -3.74] | <0.001 | -5.71 [-7.63, -3.79] | <0.001 |
| **Outcome**: Dementia | | | | | | |
| SCD-/MNPD+ | 0.88 [-0.11, 1.87] | 0.083 | 0.93 [-0.08, 1.94] | 0.072 | 0.93 [-0.09, 1.94] | 0.074 |
| SCD+/MNPD- | -0.42 [-1.06, 0.21] | 0.192 | -0.40 [-1.04, 0.23] | 0.215 | -0.41 [-1.06, 0.23] | 0.211 |
| SCD+/MNPD+ | -3.98 [-6.69, -1.27] | 0.004 | -3.83 [-6.58, -1.08] | 0.006 | -3.86 [-6.61, -1.11] | 0.006 |

Notes. Reference group: SCD-/MNPD-. Model 1: adjustment for the effects of baseline age, years of education, sex/gender, and study cohort. Model 2: additional adjustment of the effects of BMI, past/present smoking, hypertension, hypercholesterolemia, and diabetes. All vascular factors were coded 0 = absent, 1 = present. Model 3: additional adjustment for APOE-ε4 positivity (coded 0 = no ε4 alleles, 1 = 1/2 ε4 alleles). Abbreviations: APOE = Apolipoprotein E; MCI = mild cognitive impairment; MNPD = minor neuropsychological deficits (present +, absent -); SCD = subjective cognitive decline (present +, absent -).

## Table S18. Logistic regression results (Pooled samples and separate cohorts)

| | Pooled (ADNI, DELCODE, NACC) | | NACC | | Pooled (ADNI, DELCODE) | | ADNI | | DELCODE | |
|---|---|---|---|---|---|---|---|---|---|---|
| | **OR [95% CI]** | ***p*** | **OR [95% CI]** | ***p*** | **OR [95% CI]** | ***p*** | **OR [95% CI]** | ***p*** | **OR [95% CI]** | ***p*** |
| **Outcome**: Amyloid positivity | | | | | | | | | | |
| SCD-/MNPD+ | 1.04 [0.66, 1.66] | 0.853 | 1.01 [0.59, 1.73] | 0.979 | 1.14 [0.46, 2.81] | 0.771 | 1.11 [0.42, 2.96] | 0.827 | 1.08 [0.10, 11.42] | 0.947 |
| SCD+/MNPD- | 1.47 [1.20, 1.81] | <0.001 | 1.45 [1.11, 1.91] | 0.007 | 1.52 [1.10, 2.10] | 0.011 | 1.66 [1.13, 2.44] | 0.011 | 1.22 [0.68, 2.19] | 0.507 |
| SCD+/MNPD+ | 1.64 [1.04, 2.59] | 0.034 | 1.32 [0.68, 2.59] | 0.414 | 1.97 [1.03, 3.79] | 0.042 | 1.36 [0.48, 3.83] | 0.560 | 2.01 [0.80, 5.08] | 0.138 |
| **Outcome**: Tau positivity | | | | | | | | | | |
| SCD-/MNPD+ | 0.71 [0.31, 1.58] | 0.398 | 0.48 [0.14, 1.60] | 0.234 | 1.07 [0.35, 3.26] | 0.912 | 0.80 [0.23, 2.83] | 0.729 | 5.55 [0.47, 65.90] | 0.175 |
| SCD+/MNPD- | 1.20 [0.89, 1.62] | 0.239 | 1.40 [0.90, 2.19] | 0.135 | 1.11 [0.74, 1.66] | 0.621 | 1.00 [0.62, 1.60] | 0.997 | 1.72 [0.66, 4.44] | 0.266 |
| SCD+/MNPD+ | 2.10 [1.13, 3.90] | 0.020 | 1.07 [0.30, 3.75] | 0.917 | 2.73 [1.28, 5.81] | 0.009 | 2.68 [0.90, 8.00] | 0.077 | 3.82 [1.10, 13.22] | 0.034 |

Notes. Reference group: SCD-/MNPD-. All models were adjusted for the effects of baseline age, sex/gender, and study cohort (in analyses with pooled samples). Abbreviations: MNPD = minor neuropsychological deficits (present +, absent -); SCD = subjective cognitive decline (present +, absent -).

## Table S19. Multinomial logistic regression results (Pooled samples and separate cohorts)

| | Pooled (ADNI, DELCODE, NACC) | | NACC | | Pooled (ADNI, DELCODE) | | ADNI | | DELCODE | |
|---|---|---|---|---|---|---|---|---|---|---|
| | **OR [95% CI]** | ***p*** | **OR [95% CI]** | ***p*** | **OR [95% CI]** | ***p*** | **OR [95% CI]** | ***p*** | **OR [95% CI]** | ***p*** |
| **Outcome**: A+T- | | | | | | | | | | |
| SCD-/MNPD+ | 0.65 [0.29, 1.47] | 0.303 | 0.66 [0.23, 1.93] | 0.452 | 0.64 [0.18, 2.25] | 0.486 | 0.74 [0.21, 2.68] | 0.650 | – | – |
| SCD+/MNPD- | 1.23 [0.92, 1.66] | 0.166 | 0.99 [0.60, 1.62] | 0.953 | 1.41 [0.96, 2.06] | 0.076 | 1.57 [0.99, 2.51] | 0.057 | 1.12 [0.58, 2.13] | 0.740 |
| SCD+/MNPD+ | 0.81 [0.36, 1.82] | 0.616 | 0.32 [0.04, 2.49] | 0.279 | 1.16 [0.46, 2.90] | 0.755 | 0.42 [0.05, 3.42] | 0.416 | 1.33 [0.42, 4.15] | 0.627 |
| **Outcome**: A-T+ | | | | | | | | | | |
| SCD-/MNPD+ | 0.33 [0.08, 1.38] | 0.129 | 0.27 [0.04, 2.01] | 0.200 | 0.45 [0.06, 3.51] | 0.447 | 0.46 [0.06, 3.65] | 0.465 | – | – |
| SCD+/MNPD- | 0.94 [0.61, 1.46] | 0.782 | 1.14 [0.62, 2.09] | 0.668 | 0.79 [0.42, 1.50] | 0.476 | 0.73 [0.35, 1.50] | 0.385 | 1.47 [0.28, 7.68] | 0.645 |
| SCD+/MNPD+ | 0.84 [0.25, 2.83] | 0.779 | 0.56 [0.07, 4.35] | 0.581 | 1.06 [0.23, 4.90] | 0.945 | 0.78 [0.09, 6.51] | 0.816 | 2.24 [0.18, 27.69] | 0.530 |
| **Outcome**: A+T+ | | | | | | | | | | |
| SCD-/MNPD+ | 1.09 [0.42, 2.86] | 0.855 | 0.73 [0.17, 3.20] | 0.673 | 1.55 [0.42, 5.68] | 0.508 | 1.07 [0.23, 5.02] | 0.933 | 6.94 [0.52, 91.87] | 0.142 |
| SCD+/MNPD- | 1.62 [1.09, 2.39] | 0.017 | 1.77 [0.95, 3.30] | 0.070 | 1.63 [0.98, 2.73] | 0.062 | 1.59 [0.87, 2.89] | 0.129 | 1.92 [0.61, 6.05] | 0.268 |
| SCD+/MNPD+ | 3.19 [1.56, 6.56] | 0.002 | 1.50 [0.33, 6.91] | 0.603 | 4.43 [1.84, 10.63] | 0.001 | 4.06 [1.19, 13.82] | 0.025 | 5.04 [1.17, 21.69] | 0.030 |

Notes. Reference group (clinical groups): SCD-/MNPD-. Reference category (multiple logistic regression): A-T-. All models were adjusted for the effects of baseline age, sex/gender, and study cohort (in analyses with pooled samples). Abbreviations: A = amyloid positivity (present +, absent -); MNPD = minor neuropsychological deficits (present +, absent -); SCD = subjective cognitive decline (present +, absent -); T = tau positivity (present +, absent -).

## Table S20. Baseline characteristics of participants with biomarker data (Participants with data on race and ethnicity)

| Participants with biomarker data | SCD-/MNPD- | SCD-/MNPD+ | SCD+/MNPD- | SCD+/MNPD+ |
|---|---|---|---|---|
| n | 1508 | 118 | 574 | 69 |
| Age (years), M (SD) | 70.7 (7.2) | 69.3 (7.0) | 71.6 (7.6) | 70.6 (7.9) |
| Education (years), M (SD) | 16.5 (2.3) | 16.5 (2.5) | 16.5 (2.5) | 16.4 (2.6) |
| Sex (female), n (%) | 925 (61.3%) | 59 (50.0%) | 330 (57.5%) | 44 (63.8%) |
| CDR-SOB, M (SD) | 0.1 (0.3) | 0.1 (0.4) | 0.3 (0.6) | 0.4 (0.6) |
| Race | | | | |
| White | 1317 (87.3%) | 86 (72.9%) | 492 (85.7%) | 53 (76.8%) |
| Black / African American | 127 (8.4%) | 21 (17.8%) | 49 (8.5%) | 13 (18.8%) |
| American Indian / Alaska Native | 9 (0.6%) | 3 (2.5%) | 2 (0.3%) | 0 (0%) |
| Native Hawaiian / Pacific Islander | 1 (0.1%) | 0 (0%) | 0 (0%) | 0 (0%) |
| Asian | 22 (1.5%) | 3 (2.5%) | 16 (2.8%) | 0 (0%) |
| Multiracial | 32 (2.1%) | 5 (4.2%) | 15 (2.6%) | 3 (4.3%) |
| Ethnicity (Hispanic), n (%) | 55 (3.6%) | 15 (12.7%) | 24 (4.2%) | 11 (15.9%) |
| Amyloid positivity, n (%)[a] | 316 (21.0%) | 24 (20.3%) | 174 (30.4%) | 18 (26.5%) |
| Tau positivity, n (%)[b] | 149 (15.6%) | 6 (9.2%) | 67 (18.6%) | 9 (24.3%) |
| AT biomarker classification[c] | | | | |
| A-T-, n (%) | 667 (69.8%) | 52 (80.0%) | 227 (63.2%) | 25 (69.4%) |
| A+T-, n (%) | 140 (14.6%) | 7 (10.8%) | 65 (18.1%) | 2 (5.6%) |
| A-T+, n (%) | 82 (8.6%) | 2 (3.1%) | 26 (7.2%) | 2 (5.6%) |
| A+T+, n (%) | 67 (7.0%) | 4 (6.2%) | 41 (11.4%) | 7 (19.4%) |
| Follow-up (years), M (SD) | 2.9 (2.9) | 2.1 (2.4) | 2.9 (2.8) | 2.2 (2.3) |
| Progression to MCI, n (%)[d] | 62 (5.8%) | 13 (17.6%) | 44 (10.8%) | 14 (32.6%) |
| Progression to dementia, n (%)[e] | 24 (2.1%) | 1 (1.3%) | 13 (3.0%) | 2 (4.5%) |

Notes. Years of follow-up were truncated at 10 years (maximum follow-up in regression analyses). Abbreviations: A = amyloid positivity (present +, absent -); CDR-SOB = Clinical Dementia Rating – Sum of Boxes; MCI = mild cognitive impairment; MNPD = minor neuropsychological deficits (present +, absent -); SCD = subjective cognitive decline (present +, absent -); T = tau positivity (present +, absent -).

[a] Data missing in 4 individuals (n=2 SCD-/MNPD-; n=1 SCD+/MNPD-; n=1 SCD+/MNPD+)
[b] Data missing in 849 individuals (n=550 SCD-/MNPD-; n=53 SCD-/MNPD+; n=214 SCD+/MNPD-; n=32 SCD+/MNPD+)
[c] Data missing in 853 individuals (n=552 SCD-/MNPD-; n=53 SCD-/MNPD+; n=215 SCD+/MNPD-; n=33 SCD+/MNPD+)
[d] Data missing in 670 individuals (n=435 SCD-/MNPD-; n=44 SCD-/MNPD+; n=165 SCD+/MNPD-; n=26 SCD+/MNPD+)
[e] Data missing in 572 individuals (n=373 SCD-/MNPD-; n=40 SCD-/MNPD+; n=134 SCD+/MNPD-; n=25 SCD+/MNPD+)

## Table S21. Logistic regression results (without / with adjustment for race and ethnicity)

| | Model 1 (Pooled ADNI, NACC) | | Model 2 (Pooled ADNI, NACC) | |
|---|---|---|---|---|
| | **OR [95% CI]** | ***p*** | **OR [95% CI]** | ***p*** |
| **Outcome**: Amyloid positivity | | | | |
| SCD-/MNPD+ | 1.08 [0.67, 1.72] | 0.763 | 1.19 [0.74, 1.92] | 0.477 |
| SCD+/MNPD- | 1.55 [1.24, 1.93] | 0.000 | 1.57 [1.25, 1.96] | 0.000 |
| SCD+/MNPD+ | 1.35 [0.77, 2.38] | 0.294 | 1.44 [0.81, 2.56] | 0.211 |
| **Outcome**: Tau positivity | | | | |
| SCD-/MNPD+ | 0.62 [0.26, 1.47] | 0.278 | 0.63 [0.26, 1.51] | 0.301 |
| SCD+/MNPD- | 1.15 [0.83, 1.59] | 0.402 | 1.15 [0.83, 1.60] | 0.394 |
| SCD+/MNPD+ | 1.69 [0.77, 3.71] | 0.195 | 1.69 [0.76, 3.75] | 0.200 |

Notes. Reference group: SCD-/MNPD-. Model 1: adjustment for the effects of baseline age, sex/gender, and study cohort. Model 2: additional adjustment of the effects of race and Hispanic ethnicity. Race was coded as 0 = White (reference group), 1 = Black or African American, 3 = American Indian or Alaska Native, 4 = Native Hawaiian or Pacific Islander, 5 = Asian, 6 = Multiracial. Hispanic ethnicity was coded as 0 = No (reference group), 1 = Yes. Abbreviations: MNPD = minor neuropsychological deficits (present +, absent -); SCD = subjective cognitive decline (present +, absent -).

## Table S22. Multinomial logistic regression results (without / with adjustment for race and ethnicity)

| | Model 1 (Pooled ADNI, NACC) | | Model 2 (Pooled ADNI, NACC) | |
|---|---|---|---|---|
| | **OR [95% CI]** | ***p*** | **OR [95% CI]** | ***p*** |
| **Outcome**: A+T- | | | | |
| SCD-/MNPD+ | 0.73 [0.32, 1.65] | 0.449 | 0.79 [0.35, 1.80] | 0.574 |
| SCD+/MNPD- | 1.29 [0.92, 1.80] | 0.142 | 1.28 [0.91, 1.79] | 0.158 |
| SCD+/MNPD+ | 0.36 [0.08, 1.56] | 0.173 | 0.37 [0.08, 1.58] | 0.179 |
| **Outcome**: A-T+ | | | | |
| SCD-/MNPD+ | 0.34 [0.08, 1.43] | 0.141 | 0.36 [0.09, 1.53] | 0.167 |
| SCD+/MNPD- | 0.88 [0.55, 1.41] | 0.592 | 0.87 [0.55, 1.40] | 0.576 |
| SCD+/MNPD+ | 0.62 [0.14, 2.70] | 0.526 | 0.65 [0.15, 2.82] | 0.563 |
| **Outcome**: A+T+ | | | | |
| SCD-/MNPD+ | 0.92 [0.32, 2.68] | 0.886 | 0.93 [0.32, 2.72] | 0.894 |
| SCD+/MNPD- | 1.63 [1.06, 2.49] | 0.026 | 1.65 [1.07, 2.53] | 0.022 |
| SCD+/MNPD+ | 2.56 [1.03, 6.36] | 0.043 | 2.48 [0.99, 6.19] | 0.052 |

Notes. Reference group (clinical groups): SCD-/MNPD-. Reference category (multiple logistic regression): A-T-. Model 1: adjustment for the effects of baseline age, sex/gender, and study cohort. Model 2: additional adjustment of the effects of race and Hispanic ethnicity. Race was coded as 0 = White (reference group), 1 = Black or African American, 3 = American Indian or Alaska Native, 4 = Native Hawaiian or Pacific Islander, 5 = Asian, 6 = Multiracial. Hispanic ethnicity was coded as 0 = No (reference group), 1 = Yes. Abbreviations: A = amyloid positivity (present +, absent -); MNPD = minor neuropsychological deficits (present +, absent -); SCD = subjective cognitive decline (present +, absent -); T = tau positivity (present +, absent -).

## Table S23. Baseline characteristics of participants with biomarker data (Including biomarker type)

| Participants with biomarker data | SCD-/MNPD- | SCD-/MNPD+ | SCD+/MNPD- | SCD+/MNPD+ |
|---|---|---|---|---|
| n | 1608 | 125 | 786 | 97 |
| Age (years), M (SD) | 70.6 (7.1) | 69.2 (6.8) | 71.4 (7.2) | 71.2 (7.3) |
| Education (years), M (SD) | 16.4 (2.4) | 16.4 (2.5) | 16.1 (2.8) | 16.3 (2.7) |
| Sex (female), n (%) | 979 (60.9%) | 60 (48.0%) | 421 (53.6%) | 54 (55.7%) |
| CDR-SOB, M (SD) | 0.1 (0.3) | 0.1 (0.4) | 0.3 (0.6) | 0.5 (0.7) |
| Amyloid positivity, n (%)[a] | 342 (21.3%) | 25 (20.0%) | 245 (31.2%) | 32 (33.3%) |
| Tau positivity, n (%)[b] | 156 (14.8%) | 7 (9.9%) | 93 (17.0%) | 16 (25.8%) |
| Amyloid biomarker type (CSF), n (%) | 464 (28.9%) | 30 (24.0%) | 264 (33.6%) | 26 (27.1%) |
| Tau biomarker type (CSF), n (%) | 606 (57.5%) | 39 (54.9%) | 383 (69.9%) | 43 (69.4%) |
| AT biomarker classification[c] | | | | |
| A-T-, n (%) | 735 (69.9%) | 57 (80.3%) | 345 (63.1%) | 37 (60.7%) |
| A+T-, n (%) | 160 (15.2%) | 7 (9.9%) | 109 (19.9%) | 8 (13.1%) |
| A-T+, n (%) | 84 (8.0%) | 2 (2.8%) | 33 (6.0%) | 3 (4.9%) |
| A+T+, n (%) | 72 (6.9%) | 5 (7.0%) | 60 (11.0%) | 13 (21.3%) |
| Follow-up (years), M (SD) | 3.1 (2.9) | 2.1 (2.5) | 3.5 (2.9) | 2.9 (2.5) |
| Progression to MCI, n (%)[d] | 70 (6.0%) | 14 (17.9%) | 98 (16.0%) | 31 (44.9%) |
| Progression to dementia, n (%)[e] | 24 (2.0%) | 1 (1.2%) | 21 (3.3%) | 7 (10.0%) |

Notes. Years of follow-up were truncated at 10 years (maximum follow-up in regression analyses). Amyloid and tau biomarker types were coded as 0=PET and 1=CSF. Abbreviations: A = amyloid positivity (present +, absent -); CDR-SOB = Clinical Dementia Rating – Sum of Boxes; CSF = cerebrospinal fluid; MCI = mild cognitive impairment; MNPD = minor neuropsychological deficits (present +, absent -); PET = positron emission tomography; SCD = subjective cognitive decline (present +, absent -); T = tau positivity (present +, absent -).

[a] Data missing in 4 individuals (n=2 SCD-/MNPD-; n=1 SCD+/MNPD-; n=1 SCD+/MNPD+)
[b] Data missing in 882 individuals (n=555 SCD-/MNPD-; n=54 SCD-/MNPD+; n=238 SCD+/MNPD-; n=35 SCD+/MNPD+)
[c] Data missing in 886 individuals (n=557 SCD-/MNPD-; n=54 SCD-/MNPD+; n=239 SCD+/MNPD-; n=36 SCD+/MNPD+)
[d] Data missing in 691 individuals (n=444 SCD-/MNPD-; n=47 SCD-/MNPD+; n=172 SCD+/MNPD-; n=28 SCD+/MNPD+)
[e] Data missing in 593 individuals (n=382 SCD-/MNPD-; n=43 SCD-/MNPD+; n=141 SCD+/MNPD-; n=27 SCD+/MNPD+)

## Table S24. Logistic regression results (without / with adjustment for biomarker type)

| | Model 1 (ADNI, DELCODE, NACC) | | Model 2 (ADNI, DELCODE, NACC) | |
|---|---|---|---|---|
| | OR [95% CI] | *p* | OR [95% CI] | *p* |
| **Outcome**: Amyloid positivity | | | | |
| SCD-/MNPD+ | 1.04 [0.66, 1.66] | 0.853 | 1.05 [0.66-1.67] | 0.826 |
| SCD+/MNPD- | 1.47 [1.20, 1.81] | <0.001 | 1.49 [1.21-1.84] | <0.001 |
| SCD+/MNPD+ | 1.64 [1.04, 2.59] | 0.034 | 1.69 [1.07-2.68] | 0.025 |
| **Outcome**: Tau positivity | | | | |
| SCD-/MNPD+ | 0.71 [0.31, 1.58] | 0.398 | 0.73 [0.32-1.66] | 0.451 |
| SCD+/MNPD- | 1.20 [0.89, 1.62] | 0.239 | 1.22 [0.90-1.66] | 0.207 |
| SCD+/MNPD+ | 2.10 [1.13, 3.90] | 0.020 | 2.30 [1.21-4.36] | 0.011 |

Notes. Reference group: SCD-/MNPD-. Model 1: adjustment for the effects of baseline age, sex/gender, and study cohort. Model 2: additional adjustment of the effect of amyloid (model with amyloid positivity as outcome) or tau (model with tau positivity as outcome) biomarker type. Biomarker type was coded as 0 = PET (reference group), 1 = CSF. Abbreviations: CSF = cerebrospinal fluid; MNPD = minor neuropsychological deficits (present +, absent -); PET = positron emission tomography; SCD = subjective cognitive decline (present +, absent -).

## Table S25. Multinomial logistic regression results (without / with adjustment for biomarker type)

| | Model 1 (ADNI, DELCODE, NACC) | | Model 2 (ADNI, DELCODE, NACC) | |
|---|---|---|---|---|
| | **OR [95% CI]** | ***p*** | **OR [95% CI]** | ***p*** |
| **Outcome**: A+T- | | | | |
| SCD-/MNPD+ | 0.65 [0.29, 1.47] | 0.303 | 0.64 [0.29-1.45] | 0.290 |
| SCD+/MNPD- | 1.23 [0.92, 1.66] | 0.166 | 1.27 [0.94-1.71] | 0.119 |
| SCD+/MNPD+ | 0.81 [0.36, 1.82] | 0.616 | 0.86 [0.38-1.92] | 0.706 |
| **Outcome**: A-T+ | | | | |
| SCD-/MNPD+ | 0.33 [0.08, 1.38] | 0.129 | 0.34 [0.08-1.44] | 0.143 |
| SCD+/MNPD- | 0.94 [0.61, 1.46] | 0.782 | 0.91 [0.58-1.44] | 0.697 |
| SCD+/MNPD+ | 0.84 [0.25, 2.83] | 0.779 | 0.88 [0.25-3.04] | 0.839 |
| **Outcome**: A+T+ | | | | |
| SCD-/MNPD+ | 1.09 [0.42, 2.86] | 0.855 | 1.13 [0.43-2.98] | 0.804 |
| SCD+/MNPD- | 1.62 [1.09, 2.39] | 0.017 | 1.62 [1.09-2.42] | 0.017 |
| SCD+/MNPD+ | 3.19 [1.56, 6.56] | 0.002 | 3.33 [1.60-6.92] | 0.001 |

Notes. Reference group (clinical groups): SCD-/MNPD-. Reference category (multiple logistic regression): A-T-. Model 1: adjustment for the effects of baseline age, sex/gender, and study cohort. Model 2: additional adjustment of the effect of amyloid and tau biomarker type. Biomarker types were coded as 0 = PET (reference group), 1 = CSF. Abbreviations: A = amyloid positivity (present +, absent -); CSF = cerebrospinal fluid; MNPD = minor neuropsychological deficits (present +, absent -); PET = positron emission tomography; SCD = subjective cognitive decline (present +, absent -); T = tau positivity (present +, absent -).

**Table S26. Baseline characteristics of participants with biomarker data (Participants with data on vascular risk factors and APOE)**

| Participants with biomarker data | SCD-/MNPD- | SCD-/MNPD+ | SCD+/MNPD- | SCD+/MNPD+ |
|---|---|---|---|---|
| n | 1386 | 102 | 696 | 86 |
| Age (years), M (SD) | 70.8 (7.1) | 69.7 (6.5) | 71.8 (7.0) | 71.3 (7.5) |
| Education (years), M (SD) | 16.4 (2.4) | 16.3 (2.6) | 16.0 (2.8) | 16.5 (2.7) |
| Sex (female), n (%) | 837 (60.4%) | 51 (50.0%) | 369 (53.0%) | 47 (54.7%) |
| CDR-SOB, M (SD) | 0.1 (0.2) | 0.1 (0.4) | 0.3 (0.5) | 0.4 (0.6) |
| APOE-ε4+, n (%) | 464 (33.5%) | 30 (29.4%) | 231 (33.2%) | 38 (44.2%) |
| BMI, M (SD) | 27.4 (5.2) | 28.8 (5.5) | 27.0 (4.9) | 27.2 (5.2) |
| Past / Present smoking, n (%) | 473 (34.1%) | 38 (37.3%) | 262 (37.6%) | 35 (40.7%) |
| Hypertension, n (%) | 569 (41.1%) | 57 (55.9%) | 320 (46.0%) | 46 (53.5%) |
| Hypercholesterolemia, n (%) | 617 (44.5%) | 54 (52.9%) | 298 (42.8%) | 36 (41.9%) |
| Diabetes, (%) | 124 (8.9%) | 18 (17.6%) | 77 (11.1%) | 12 (14.0%) |
| Amyloid positivity, n (%)[a] | 295 (21.3%) | 21 (20.6%) | 225 (32.3%) | 27 (31.8%) |
| Tau positivity, n (%)[b] | 148 (15.7%) | 6 (10.0%) | 86 (17.3%) | 12 (21.4%) |
| AT biomarker classification[c] | | | | |
| A-T-, n (%) | 651 (69.3%) | 48 (80.0%) | 310 (62.2%) | 35 (63.6%) |
| A+T-, n (%) | 141 (15.0%) | 6 (10.0%) | 102 (20.5%) | 8 (14.5%) |
| A-T+, n (%) | 80 (8.5%) | 2 (3.3%) | 31 (6.2%) | 2 (3.6%) |
| A+T+, n (%) | 68 (7.2%) | 4 (6.7%) | 55 (11.0%) | 10 (18.2%) |
| Follow-up (years), M (SD) | 3.4 (2.9) | 2.4 (2.6) | 3.8 (2.8) | 3.2 (2.5) |
| Progression to MCI, n (%)[d] | 67 (6.2%) | 13 (19.4%) | 92 (16.1%) | 28 (43.8%) |
| Progression to dementia, n (%)[e] | 24 (2.1%) | 1 (1.4%) | 19 (3.2%) | 7 (10.8%) |

Notes. Years of follow-up were truncated at 10 years (maximum follow-up in regression analyses). Abbreviations: A = amyloid positivity (present +, absent -); APOE = Apolipoprotein E; BMI = Body Mass Index; CDR-SOB = Clinical Dementia Rating – Sum of Boxes; MCI = mild cognitive impairment; MNPD = minor neuropsychological deficits (present +, absent -); SCD = subjective cognitive decline (present +, absent -); T = tau positivity (present +, absent -).

[a] Data missing in 3 individuals (n=2 SCD-/MNPD-; n=1 SCD+/MNPD+)
[b] Data missing in 714 individuals (n=444 SCD-/MNPD-; n=42 SCD-/MNPD+; n=198 SCD+/MNPD-; n=30 SCD+/MNPD+)
[c] Data missing in 717 individuals (n=446 SCD-/MNPD-; n=42 SCD-/MNPD+; n=198 SCD+/MNPD-; n=31 SCD+/MNPD+)
[d] Data missing in 491 individuals (n=309 SCD-/MNPD-; n=35 SCD-/MNPD+; n=125 SCD+/MNPD-; n=22 SCD+/MNPD+)
[e] Data missing in 415 individuals (n=260 SCD-/MNPD-; n=32 SCD-/MNPD+; n=102 SCD+/MNPD-; n=21 SCD+/MNPD+)

## Table S27. Logistic regression results (without / with adjustment for vascular risk factors and APOE)

| | Model 1 (ADNI, DELCODE, NACC) | | Model 2 (ADNI, DELCODE, NACC) | | Model 3 (ADNI, DELCODE, NACC) | |
|---|---|---|---|---|---|---|
| **All participants** | **OR [95% CI]** | ***p*** | **OR [95% CI]** | ***p*** | **OR [95% CI]** | ***p*** |
| **Outcome**: Amyloid positivity | | | | | | |
| SCD-/MNPD+ | 1.07 [0.65, 1.78] | 0.782 | 1.08 [0.65, 1.80] | 0.759 | 1.19 [0.69, 2.02] | 0.533 |
| SCD+/MNPD- | 1.52 [1.22, 1.89] | <0.001 | 1.52 [1.22, 1.90] | <0.001 | 1.54 [1.22, 1.94] | <0.001 |
| SCD+/MNPD+ | 1.52 [0.93, 2.48] | 0.098 | 1.53 [0.93, 2.51] | 0.093 | 1.24 [0.73, 2.13] | 0.427 |
| **Outcome**: Tau positivity | | | | | | |
| SCD-/MNPD+ | 0.66 [0.28, 1.58] | 0.354 | 0.67 [0.28, 1.61] | 0.374 | 0.73 [0.30, 1.76] | 0.482 |
| SCD+/MNPD- | 1.14 [0.83, 1.55] | 0.413 | 1.14 [0.83, 1.56] | 0.410 | 1.14 [0.83, 1.57] | 0.412 |
| SCD+/MNPD+ | 1.53 [0.77, 3.03] | 0.229 | 1.53 [0.76, 3.06] | 0.229 | 1.42 [0.70, 2.87] | 0.335 |

Notes. Reference group: SCD-/MNPD-. Model 1: adjustment for the effects of baseline age, years of education, sex/gender, and study cohort. Model 2: additional adjustment of the effects of BMI, past/present smoking, hypertension, hypercholesterolemia, and diabetes. All vascular factors were coded 0 = absent, 1 = present. Model 3: additional adjustment for APOE-ε4 positivity (coded 0 = no ε4 alleles, 1 = 1/2 ε4 alleles). Abbreviations: APOE = Apolipoprotein E; MNPD = minor neuropsychological deficits (present +, absent -); SCD = subjective cognitive decline (present +, absent -).

## Table S28. Multinomial logistic regression results (without / with adjustment for vascular risk factors and APOE)

| | Model 1 (ADNI, DELCODE, NACC) | | Model 2 (ADNI, DELCODE, NACC) | | Model 3 (ADNI, DELCODE, NACC) | |
|---|---|---|---|---|---|---|
| **All participants** | **OR [95% CI]** | ***p*** | **OR [95% CI]** | ***p*** | **OR [95% CI]** | ***p*** |
| **Outcome**: A+T- | | | | | | |
| SCD-/MNPD+ | 0.65 [0.27, 1.57] | 0.340 | 0.61 [0.25, 1.48] | 0.273 | 0.72 [0.29, 1.79] | 0.476 |
| SCD+/MNPD- | 1.25 [0.92, 1.71] | 0.159 | 1.23 [0.90, 1.68] | 0.195 | 1.24 [0.89, 1.72] | 0.204 |
| SCD+/MNPD+ | 0.85 [0.38, 1.90] | 0.685 | 0.83 [0.37, 1.86] | 0.645 | 0.69 [0.29, 1.65] | 0.410 |
| **Outcome**: A-T+ | | | | | | |
| SCD-/MNPD+ | 0.36 [0.09, 1.53] | 0.167 | 0.35 [0.08, 1.46] | 0.149 | 0.36 [0.08, 1.53] | 0.165 |
| SCD+/MNPD- | 0.90 [0.57, 1.42] | 0.656 | 0.89 [0.57, 1.41] | 0.620 | 0.89 [0.57, 1.41] | 0.622 |
| SCD+/MNPD+ | 0.53 [0.12, 2.30] | 0.400 | 0.51 [0.12, 2.23] | 0.373 | 0.51 [0.12, 2.20] | 0.363 |
| **Outcome**: A+T+ | | | | | | |
| SCD-/MNPD+ | 0.96 [0.33, 2.78] | 0.934 | 1.00 [0.34, 2.91] | 0.994 | 1.19 [0.40, 3.58] | 0.756 |
| SCD+/MNPD- | 1.53 [1.02, 2.30] | 0.041 | 1.53 [1.01, 2.31] | 0.042 | 1.57 [1.03, 2.41] | 0.037 |
| SCD+/MNPD+ | 2.42 [1.10, 5.33] | 0.028 | 2.45 [1.10, 5.44] | 0.028 | 2.02 [0.86, 4.78] | 0.108 |

Notes. Reference group (clinical groups): SCD-/MNPD-. Reference category (multiple logistic regression): A-T-. Model 1: adjustment for the effects of baseline age, years of education, sex/gender, and study cohort. Model 2: additional adjustment of the effects of BMI, past/present smoking, hypertension, hypercholesterolemia, and diabetes. All vascular factors were coded 0 = absent, 1 = present. Model 3: additional adjustment APOE-ε4 positivity (coded 0 = no ε4 alleles, 1 = 1/2 ε4 alleles). Abbreviations: A = amyloid positivity (present +, absent -); APOE = Apolipoprotein E; MNPD = minor neuropsychological deficits (present +, absent -); SCD = subjective cognitive decline (present +, absent -); T = tau positivity (present +, absent -).

## Table S29. Baseline characteristics of amyloid-positive participants with progression data (Participants with data on race and ethnicity)

| Amyloid-positive participants | SCD-/MNPD- | SCD-/MNPD+ | SCD+/MNPD- | SCD+/MNPD+ |
|---|---|---|---|---|
| n | 231 | 16 | 135 | 11 |
| Age (years), M (SD) | 73.0 (7.0) | 71.9 (6.2) | 73.8 (6.9) | 76.1 (4.5) |
| Education (years), M (SD) | 16.6 (2.5) | 17.1 (2.8) | 16.3 (2.8) | 17.2 (2.4) |
| Sex (female), n (%) | 140 (60.6%) | 8 (50.0%) | 76 (56.3%) | 7 (63.6%) |
| CDR-SOB, M (SD) | 0.1 (0.3) | 0.0 (0.0) | 0.2 (0.6) | 0.5 (0.7) |
| Race | | | | |
| White | 212 (91.8%) | 16 (100.0%) | 123 (91.1%) | 9 (81.8%) |
| Black / African American | 11 (4.8%) | 0 (0%) | 7 (5.2%) | 2 (18.2%) |
| American Indian / Alaska Native | 0 (0%) | 0 (0%) | 0 (0%) | 0 (0%) |
| Native Hawaiian / Pacific Islander | 0 (0%) | 0 (0%) | 0 (0%) | 0 (0%) |
| Asian | 3 (1.3%) | 0 (0%) | 2 (1.5%) | 0 (0%) |
| Multiracial | 5 (2.2%) | 0 (0%) | 3 (2.2%) | 0 (0%) |
| Ethnicity (Hispanic), n (%) | 5 (2.2%) | 1 (6.2%) | 3 (2.2%) | 1 (9.1%) |
| Follow-up (years), M (SD) | 3.7 (2.7) | 3.7 (2.7) | 3.4 (2.5) | 3.5 (2.5) |
| Progression to MCI, n (%)[a] | 26 (11.8%) | 5 (33.3%) | 26 (20.6%) | 8 (72.7%) |
| Progression to dementia, n (%)[b] | 13 (5.6%) | 0 (0%) | 9 (6.7%) | 2 (18.2%) |

Notes. Years of follow-up were truncated at 10 years (maximum follow-up in regression analyses). Abbreviations: CDR-SOB = Clinical Dementia Rating – Sum of Boxes; MCI = mild cognitive impairment; MNPD = minor neuropsychological deficits (present +, absent -); SCD = subjective cognitive decline (present +, absent -).

[a] Data missing in 20 individuals (n=10 SCD-/MNPD-; n=1 SCD-/MNPD+; n=9 SCD+/MNPD-)
[b] Data missing in 1 individual (n=1 SCD+/MNPD-)

## Table S30. Baseline characteristics of amyloid-positive participants with progression data (Including biomarker type)

| Amyloid-positive participants | SCD-/MNPD- | SCD-/MNPD+ | SCD+/MNPD- | SCD+/MNPD+ |
|---|---|---|---|---|
| n | 253 | 17 | 204 | 23 |
| Age (years), M (SD) | 72.8 (6.8) | 71.9 (6.0) | 73.4 (6.4) | 75.9 (4.7) |
| Education (years), M (SD) | 16.5 (2.6) | 17.1 (2.8) | 15.9 (2.9) | 16.3 (2.9) |
| Sex (female), n (%) | 146 (57.7%) | 8 (47.1%) | 104 (51.0%) | 9 (39.1%) |
| CDR-SOB, M (SD) | 0.1 (0.3) | 0.0 (0.0) | 0.3 (0.6) | 0.6 (0.8) |
| Amyloid biomarker type (CSF), n (%) | 87 (34.4%) | 5 (29.4%) | 72 (35.3%) | 9 (39.1%) |
| Follow-up (years), M (SD) | 3.9 (2.7) | 3.9 (2.9) | 4.0 (2.5) | 3.9 (2.2) |
| Progression to MCI, n (%)[a] | 30 (12.3%) | 6 (37.5%) | 54 (27.7%) | 17 (73.9%) |
| Progression to dementia, n (%)[b] | 13 (5.1%) | 0 (0%) | 17 (8.4%) | 6 (26.1%) |

Notes. Years of follow-up were truncated at 10 years (maximum follow-up in regression analyses). Amyloid biomarker types were coded as 0=PET and 1=CSF. Abbreviations: CDR-SOB = Clinical Dementia Rating – Sum of Boxes; CSF = cerebrospinal fluid; MCI = mild cognitive impairment; MNPD = minor neuropsychological deficits (present +, absent -); PET = positron emission tomography; SCD = subjective cognitive decline (present +, absent -).

[a] Data missing in 20 individuals (n=10 SCD-/MNPD-; n=1 SCD-/MNPD+; n=9 SCD+/MNPD-)
[b] Data missing in 1 individual (n=1 SCD+/MNPD-)

## Table S31. Cox regression results (without / with adjustment for biomarker type)

| | Model 1 (ADNI, DELCODE, NACC) | | Model 2 (ADNI, DELCODE, NACC) | |
|---|---|---|---|---|
| **Amyloid-positive participants** | **HR [95% CI]** | ***p*** | **HR [95% CI]** | ***p*** |
| **Outcome**: MCI | | | | |
| SCD-/MNPD+ | 4.99 [2.03, 12.26] | <0.001 | 4.97 [2.01, 12.28] | 0.001 |
| SCD+/MNPD- | 2.36 [1.47, 3.80] | <0.001 | 2.62 [1.61, 4.26] | <0.001 |
| SCD+/MNPD+ | 11.70 [5.94, 23.04] | <0.001 | 13.63 [6.85, 27.13] | <0.001 |
| **Outcome**: Dementia | | | | |
| SCD-/MNPD+ | 0.74 [0.04, 14.00] | 0.838 | 0.67 [0.03, 12.97] | 0.789 |
| SCD+/MNPD- | 2.10 [0.97, 4.57] | 0.060 | 2.59 [1.15, 5.81] | 0.021 |
| SCD+/MNPD+ | 9.07 [2.91, 28.29] | <0.001 | 11.90 [3.79, 37.40] | <0.001 |

Notes. Reference group: SCD-/MNPD-. Model 1: adjustment for the effects of baseline age, years of education, sex/gender, and study cohort. Model 2: additional adjustment of the effect of amyloid biomarker type. Biomarker type was coded as 0 = PET (reference group), 1 = CSF. Cox regression models with Firth's penalized maximum likelihood estimation were used for the dementia progression analyses. Abbreviations: CSF = cerebrospinal fluid; MCI = mild cognitive impairment; MNPD = minor neuropsychological deficits (present +, absent -); PET = positron emission tomography; SCD = subjective cognitive decline (present +, absent -).

## Table S32. Pseudo-value regression results (without / with adjustment for biomarker type)

| | Model 1 (ADNI, DELCODE, NACC) | | Model 2 (ADNI, DELCODE, NACC) | |
|---|---|---|---|---|
| **Amyloid-positive participants** | **β [95% CI]** | ***p*** | **β [95% CI]** | ***p*** |
| **Outcome**: MCI | | | | |
| SCD-/MNPD+ | -2.60 [-5.05, -0.15] | 0.037 | -2.62 [-5.10, -0.14] | 0.038 |
| SCD+/MNPD- | -1.29 [-2.15, -0.43] | 0.003 | -1.43 [-2.29, -0.58] | 0.001 |
| SCD+/MNPD+ | -5.89 [-7.63, -4.15] | <0.001 | -6.10 [-7.78, -4.41] | <0.001 |
| **Outcome**: Dementia | | | | |
| SCD-/MNPD+ | 0.85 [-0.16, 1.85] | 0.098 | 0.82 [-0.24, 1.88] | 0.129 |
| SCD+/MNPD- | -0.49 [-1.12, 0.14] | 0.134 | -0.58 [-1.20, 0.04] | 0.067 |
| SCD+/MNPD+ | -3.51 [-5.98, -1.04] | 0.005 | -3.65 [-6.11, -1.19] | 0.004 |

Notes. Reference group: SCD-/MNPD-. Model 1: adjustment for the effects of baseline age, years of education, sex/gender, and study cohort. Model 2: additional adjustment of the effect of amyloid biomarker type. Biomarker type was coded as 0 = PET (reference group), 1 = CSF. Abbreviations: CSF = cerebrospinal fluid; MCI = mild cognitive impairment; MNPD = minor neuropsychological deficits (present +, absent -); PET = positron emission tomography; SCD = subjective cognitive decline (present +, absent -).

## Table S33. Baseline characteristics of amyloid-positive participants with progression data (Participants with data on vascular risk factors and APOE)

| Amyloid-positive participants | SCD-/MNPD- | SCD-/MNPD+ | SCD+/MNPD- | SCD+/MNPD+ |
|---|---|---|---|---|
| n | 232 | 16 | 191 | 19 |
| Age (years), M (SD) | 73.0 (6.9) | 72.2 (6.1) | 73.4 (6.4) | 76.1 (4.7) |
| Education (years), M (SD) | 16.4 (2.6) | 17.1 (2.8) | 15.9 (2.9) | 16.6 (2.6) |
| Sex (female), n (%) | 136 (58.6%) | 7 (43.8%) | 94 (49.2%) | 6 (31.6%) |
| CDR-SOB, M (SD) | 0.1 (0.3) | 0.0 (0.0) | 0.3 (0.6) | 0.6 (0.8) |
| APOE-ε4+, n (%) | 118 (50.9%) | 9 (56.2%) | 110 (57.6%) | 13 (68.4%) |
| BMI, M (SD) | 27.3 (5.5) | 27.2 (4.1) | 26.2 (4.6) | 25.9 (3.6) |
| Past / Present smoking, n (%) | 75 (32.3%) | 4 (25.0%) | 63 (33.0%) | 11 (57.9%) |
| Hypertension, n (%) | 112 (48.3%) | 10 (62.5%) | 87 (45.5%) | 12 (63.2%) |
| Hypercholesterolemia, n (%) | 100 (43.1%) | 10 (62.5%) | 77 (40.3%) | 10 (52.6%) |
| Diabetes, (%) | 18 (7.8%) | 1 (6.2%) | 17 (8.9%) | 2 (10.5%) |
| Follow-up (years), M (SD) | 4.0 (2.8) | 4.1 (2.9) | 4.2 (2.5) | 4.4 (2.2) |
| Progression to MCI, n (%)[a] | 29 (13.1%) | 6 (40.0%) | 51 (27.7%) | 14 (73.7%) |
| Progression to dementia, n (%)[b] | 13 (5.6%) | 0 (0%) | 15 (7.9%) | 6 (31.6%) |

Notes. Years of follow-up were truncated at 10 years (maximum follow-up in regression analyses). Abbreviations: APOE = Apolipoprotein E; BMI = Body Mass Index; CDR-SOB = Clinical Dementia Rating – Sum of Boxes; MCI = mild cognitive impairment; MNPD = minor neuropsychological deficits (present +, absent -); SCD = subjective cognitive decline (present +, absent -).

[a] Data missing in 18 individuals (n=10 SCD-/MNPD-; n=1 SCD-/MNPD+; n=7 SCD+/MNPD-)
[b] Data missing in 1 individual (n=1 SCD+/MNPD-)

## Table S34. Sensitivity, specificity, and predictive values for the progression to MCI within five years

| Participants with amyloid data | Cases | Survivors | Censored | Positive | Negative | Sensitivity [95% CI] | Specificity [95% CI] | PPV [95% CI] | NPV [95% CI] |
|---|---|---|---|---|---|---|---|---|---|
| **Predictor:** Clinical group | | | | | | | | | |
| Any symptom (SCD+ or MNPD+) | 153 | 647 | 1124 | 761 | 1163 | 72.2 [64.9, 79.6] | 59.4 [55.6, 63.1] | 18.6 [15.2, 22.1] | 94.3 [92.6, 96.0] |
| SCD+ | 153 | 647 | 1124 | 683 | 1241 | 65.5 [57.7, 73.2] | 61.5 [57.8, 65.3] | 18.0 [14.4, 21.5] | 93.3 [91.5, 95.1] |
| MNPD+ | 153 | 647 | 1124 | 147 | 1777 | 24.0 [17.2, 30.8] | 95.8 [94.3, 97.4] | 42.5 [30.5, 54.6] | 90.7 [89.0, 92.5] |
| SCD+/MNPD+ | 153 | 647 | 1124 | 69 | 1855 | 17.2 [11.2, 23.3] | 98.0 [96.9, 99.1] | 52.5 [35.9, 69.0] | 90.2 [88.5, 91.9] |
| **Predictor**: Amyloid pathology | **The groups listed in these rows refer to the population in which the grouping criterion (amyloid positivity) is evaluated** | | | | | | | | |
| All groups | 153 | 647 | 1124 | 477 | 1447 | 50.9 [42.6, 59.2] | 79.0 [75.8, 82.1] | 23.8 [18.6, 28.9] | 92.6 [90.9, 94.3] |
| SCD-/MNPD- | 44 | 384 | 735 | 243 | 920 | 48.6 [33.0, 64.2] | 82.6 [78.8, 86.3] | 13.8 [7.5, 20.1] | 96.5 [95.2, 97.9] |
| Any symptom (SCD+ or MNPD+) | 109 | 263 | 389 | 234 | 527 | 52.1 [42.4, 61.8] | 73.8 [68.4, 79.1] | 32.7 [25.0, 40.4] | 86.3 [82.6, 90.0] |
| SCD+ | 97 | 249 | 337 | 218 | 465 | 54.2 [44.0, 64.5] | 74.3 [68.9, 79.7] | 33.5 [25.4, 41.6] | 87.2 [83.5, 90.9] |
| MNPD+ | 40 | 27 | 80 | 39 | 108 | 54.6 [38.7, 70.6] | 74.1 [57.5, 90.7] | 57.0 [36.2, 77.7] | 72.2 [60.8, 83.6]1 |
| SCD+/MNPD+ | 28 | 13 | 28 | 23 | 46 | 61.4 [43.2, 79.5] | 84.6 [64.9, 104.4] | 80.8 [58.3, 103.3] | 67.5 [51.2, 83.8] |

Notes. Time-dependent inverse probability of censoring weighting (IPCW) estimates of sensitivity, specificity, and positive/negative predictive values.[29] Results estimate the prognostic value of different clinical criteria or of amyloid positivity in different clinical groups for the progression to MCI within five years. Abbreviations: MNPD = minor neuropsychological deficits (present +, absent -); NPV = negative predictive value; PPV = positive predictive value; SCD = subjective cognitive decline (present +, absent -).

## Table S35. Power analysis results

| | Estimated Trial Size [95% CI] | Estimated Biomarker Screenings [95% CI] |
|---|---|---|
| All groups | 969 [800, 1228] | 3930 [3683, 4213] |
| Any symptom (SCD+ or MNPD+) | 620 [505, 803] | 2065 [1887, 2280] |
| SCD+ | 622 [503, 815] | 1978 [1803, 2192] |
| MNPD+ | 290 [222, 417] | 1124 [919, 1448] |
| SCD+/MNPD+ | 214 [168, 293] | 642 [500, 895] |

Notes. The left column shows the estimated sample size per arm necessary to detect a 30% reduction in the risk of progression to MCI over 4.5 years of follow-up across different clinical inclusion criteria (all clinical groups, participants with SCD or MNPD, participants with SCD, participants with MNPD, and participants with SCD and MNPD). The right column shows the estimated number of biomarker screenings necessary to reach the estimated sample sizes. These estimates were calculated by dividing the estimated trial sizes per arm by the proportion of amyloid-positive participants in each clinical group. Abbreviations: MCI = mild cognitive impairment; MNPD = minor neuropsychological deficits (present +); SCD = subjective cognitive decline (present +).

## References


1. Mueller SG, Weiner MW, Thal LJ, et al. The Alzheimer's disease neuroimaging initiative. *Neuroimaging Clin N Am*. 2005;15(4):869-877, xi. doi:10.1016/j.nic.2005.09.008
2. Aisen PS, Petersen RC, Donohue M, Weiner MW, Alzheimer's Disease Neuroimaging Initiative. Alzheimer's Disease Neuroimaging Initiative 2 Clinical Core: Progress and plans. *Alzheimers Dement*. 2015;11(7):734-739. doi:10.1016/j.jalz.2015.05.005
3. Weiner MW, Veitch DP, Aisen PS, et al. The Alzheimer's Disease Neuroimaging Initiative 3: Continued innovation for clinical trial improvement. *Alzheimers Dement*. 2017;13(5):561-571. doi:10.1016/j.jalz.2016.10.006
4. Jessen F, Spottke A, Boecker H, et al. Design and first baseline data of the DZNE multicenter observational study on predementia Alzheimer's disease (DELCODE). *Alzheimers Res Ther*. 2018;10(1):15. doi:10.1186/s13195-017-0314-2
5. Jessen F, Wolfsgruber S, Kleineindam L, et al. Subjective cognitive decline and stage 2 of Alzheimer disease in patients from memory centers. *Alzheimers Dement*. 2023;19(2):487-497. doi:10.1002/alz.12674
6. Beekly DL, Ramos EM, Lee WW, et al. The National Alzheimer's Coordinating Center (NACC) database: the Uniform Data Set. *Alzheimer Dis Assoc Disord*. 2007;21(3):249-258. doi:10.1097/WAD.0b013e318142774e
7. Besser L, Kukull W, Knopman DS, et al. Version 3 of the National Alzheimer's Coordinating Center's Uniform Data Set. *Alzheimer Dis Assoc Disord*. 2018;32(4):351-358. doi:10.1097/WAD.0000000000000279
8. Shirk SD, Mitchell MB, Shaughnessy LW, et al. A web-based normative calculator for the uniform data set (UDS) neuropsychological test battery. *Alzheimers Res Ther*. 2011;3(6):32. doi:10.1186/alzrt94
9. Weintraub S, Besser L, Dodge HH, et al. Version 3 of the Alzheimer Disease Centers' neuropsychological test battery in the Uniform Data Set (UDS). *Alzheimer Dis Assoc Disord*. 2018;32(1):10-17. doi:10.1097/WAD.0000000000000223
10. Stricker NH, Christianson TJ, Lundt ES, et al. Mayo Normative Studies: Regression-based normative data for the Auditory Verbal Learning Test for ages 30-91 years and the importance of adjusting for sex. *J Int Neuropsychol Soc*. 2021;27(3):211-226. doi:10.1017/S1355617720000752
11. McKhann G, Drachman D, Folstein M, Katzman R, Price D, Stadlan EM. Clinical diagnosis of Alzheimer's disease: report of the NINCDS-ADRDA Work Group under the auspices of Department of Health and Human Services Task Force on Alzheimer's Disease. *Neurology*. 1984;34(7):939-944. doi:10.1212/wnl.34.7.939
12. Berres M, Monsch AU, Bernasconi F, Thalmann B, Stähelin HB. Normal ranges of neuropsychological tests for the diagnosis of Alzheimer's disease. *Stud Health Technol Inform*. 2000;77:195-199.
13. Stark M, Wolfsgruber S, Kleineidam L, et al. Relevance of minor neuropsychological deficits in patients with subjective cognitive decline. *Neurology*. 2023;101(21):e2185-e2196. doi:10.1212/WNL.0000000000207844
14. Román GC, Tatemichi TK, Erkinjuntti T, et al. Vascular dementia: Diagnostic criteria for research studies. *Neurology*. 1993;43:250-260.
15. Rascovsky K, Hodges JR, Knopman D, et al. Sensitivity of revised diagnostic criteria for the behavioural variant of frontotemporal dementia. *Brain*. 2011;134(Pt 9):2456-2477. doi:10.1093/brain/awr179
16. Mesulam MM. Primary progressive aphasia. *Ann Neurol*. 2001;49(4):425-432. doi:10.1002/ana.91
17. Gorno-Tempini ML, Hillis AE, Weintraub S, et al. Classification of primary progressive aphasia and its variants. *Neurology*. 2011;76(11):1006-1014. doi:10.1212/WNL.0b013e31821103e6
18. Ludolph A, Drory V, Hardiman O, et al. A revision of the El Escorial criteria - 2015. *Amyotroph Lateral Scler Frontotemporal Degener*. 2015;16(5-6):291-292. doi:10.3109/21678421.2015.1049183
19. McKeith IG, Dickson DW, Lowe J, et al. Diagnosis and management of dementia with Lewy bodies: third report of the DLB Consortium. *Neurology*. 2005;65(12):1863-1872. doi:10.1212/01.wnl.0000187889.17253.b1
20. McKhann GM, Knopman DS, Chertkow H, et al. The diagnosis of dementia due to Alzheimer's disease: Recommendations from the National Institute on Aging-Alzheimer's Association workgroups on diagnostic guidelines for Alzheimer's disease. *Alzheimers Dement*. 2011;7(3):263-269. doi:10.1016/j.jalz.2011.03.005
21. McKeith IG, Boeve BF, Dickson DW, et al. Diagnosis and management of dementia with Lewy bodies: Fourth consensus report of the DLB Consortium. *Neurology*. 2017;89(1):88-100. doi:10.1212/WNL.0000000000004058
22. Neary D, Snowden JS, Gustafson L, et al. Frontotemporal lobar degeneration: a consensus on clinical diagnostic criteria. *Neurology*. 1998;51(6):1546-1554. doi:10.1212/wnl.51.6.1546
23. Mesulam M-M. Primary progressive aphasia--a language-based dementia. *N Engl J Med*. 2003;349(16):1535-1542. doi:10.1056/NEJMra022435
24. Litvan I, Bhatia KP, Burn DJ, et al. Movement Disorders Society Scientific Issues Committee report: SIC Task Force appraisal of clinical diagnostic criteria for Parkinsonian disorders. *Mov Disord*. 2003;18(5):467-486. doi:10.1002/mds.10459

25. Ossenkoppele R, Pichet Binette A, Groot C, et al. Amyloid and tau PET-positive cognitively unimpaired individuals are at high risk for future cognitive decline. *Nat Med*. 2022;28(11):2381-2387. doi:10.1038/s41591-022-02049-x

26. Strikwerda-Brown C, Hobbs DA, Gonneaud J, et al. Association of Elevated Amyloid and Tau Positron Emission Tomography Signal With Near-Term Development of Alzheimer Disease Symptoms in Older Adults Without Cognitive Impairment. *JAMA Neurol*. 2022;79(10):975-985. doi:10.1001/jamaneurol.2022.2379

27. Dumurgier J, Sabia S, Zetterberg H, et al. A Pragmatic, Data-Driven Method to Determine Cutoffs for CSF Biomarkers of Alzheimer Disease Based on Validation Against PET Imaging. *Neurology*. 2022;99(7):e669-e678. doi:10.1212/WNL.0000000000200735

28. La Joie R, Ayakta N, Seeley WW, et al. Multisite study of the relationships between antemortem [11C]PIB-PET Centiloid values and postmortem measures of Alzheimer's disease neuropathology. *Alzheimers Dement*. 2019;15(2):205-216. doi:10.1016/j.jalz.2018.09.001

29. Blanche P, Dartigues J-F, Jacqmin-Gadda H. Estimating and comparing time-dependent areas under receiver operating characteristic curves for censored event times with competing risks. *Stat Med*. 2013;32(30):5381-5397. doi:10.1002/sim.5958
